# A systematic review of ultrasound-mediated drug delivery to the eye and critical insights to facilitate a timely path to the clinic

**DOI:** 10.7150/thno.82884

**Published:** 2023-06-19

**Authors:** Isaac J Rad, Luke Chapman, Karnaker Reddy Tupally, Martin Veidt, Hussain Al-Sadiq, Robert Sullivan, Harendra S Parekh

**Affiliations:** 1 The University of Queensland, School of Pharmacy, Brisbane, Queensland, Australia.; 2 The University of Queensland, Faculty of Medicine, Brisbane, Queensland, Australia.; 3 The University of Queensland, School of Mechanical and Mining Engineering, Brisbane, Queensland, Australia.; 4 Al-Asala University, Department of Industrial Engineering, Dammam, Saudi Arabia.; 5 The University of Queensland, Queensland Brain Institute, Brisbane, Queensland, Australia.

**Keywords:** acoustofluidics, ultrasound, ocular drug delivery, clinical translation, guideline

## Abstract

Ultrasound has long been identified as a promising, non-invasive modality for improving ocular drug delivery across a range of indications. Yet, with 20 years of learnings behind us, clinical translation remains limited. To help address this, and in accordance with PRISMA guidelines, the various mechanisms of ultrasound-mediated ocular drug delivery have been appraised, ranging from first principles to emergent applications spanning both *ex vivo* and *in vivo* models. The heterogeneity of study methods precluded meta-analysis, however an extensive characterisation of the included studies allowed for semi-quantitative and qualitative assessments.

**Methods:** In this review, we reflected on study quality of reporting, and risk of bias (RoB) using the latest Animal Research: Reporting of *In Vivo* Experiments (ARRIVE 2.0) guidelines, alongside the Systematic Review Centre for Laboratory animal Experimentation (SYRCLE) RoB tools. Literature studies from 2002 to 2022 were initially characterised according to methods of ultrasound application, ultrasound parameters applied, animal models employed, as well as safety and efficacy assessments. This exercise contributed to developing a comprehensive understanding of the current state of play within ultrasound-mediated ocular drug delivery. The results were then synthesised and processed into a guide to aid future study design, with the goal of improving the reliability of data, and to support efficient and timely translation to the clinic.

**Results:** Key attributes identified as hindering translation included: poor reporting quality and high RoB, skewed use of animals unrepresentative of the human eye, and the over reliance of reductionist safety assessments. E*x vivo* modelling studies were often unable to have comprehensive safety assessments performed on them, which are imperative to determining treatment safety, and represent a pre-requisite for clinical translation.

**Conclusion:** With the use of our synthesised guide, and a thorough understanding of the underlying physicochemical interactions between ultrasound and ocular biology provided herein, this review offers a firm foundation on which future studies should ideally be built, such that ultrasound-mediated ocular drug delivery can be translated from concept to the coalface where it can provide immense clinical benefit.

## 1. Introduction

### 1.1. Rationale: Current therapeutic and financial challenges in treating ocular disease

Populations are ageing in all regions of the world; median worldwide age has increased from 21.5 in 1980 to 30.2 in 2022 and current projections suggest one in six people in the world will be aged ≥ 65 years by 2050, an increase from the one in ten of 2022 1. Consequently, a rise in the prevalence of age-related disease is expected, further increasing the economic and social burden of disease associated with rising cost of healthcare and loss of productivity. In this context, sight-threatening diseases constitute a major contributor to reducing quality of life. Globally, blindness and vision loss due to common sight-threatening diseases, such as cataracts, glaucoma, age-related macular degeneration (AMD) and refraction disorders contributed a total of 22.6 million disability-adjusted life years in 2019 2. In addition, moderate and severe vision loss caused by retinopathies such as AMD and diabetic retinopathy (DRE) have increased in prevalence by 93.7% and 80.5%, respectively between 2000 and 2020, signalling an alarming need for improved treatment modalities 3, 4. Effective and sustained drug delivery to the eye is challenged by the presence of various biological barriers. The eye is an immune-privileged organ, isolated from the surrounding tissue and circulation by the blood-retinal barrier (BRB), making oral drug delivery wholly inefficient, if not implausible 5. From a trans-topical delivery perspective, the external surfaces of the eye are composed of static barriers such as the epithelium, stroma and endothelium of the cornea and sclera, as well as dynamic barriers, including blinking and continuous tear turnover. These barriers limit the residence time and ingress of topically applied therapeutics toward intraocular sites of action 6. Sight-threatening diseases originating within the globe of the eye, such as AMD and DRE often require intravitreal injections to hold/slow disease progression 7. This approach relies primarily on passive diffusive mechanisms through the vitreous for retinal drug delivery, and necessitates localised, pars plana injection of drugs at high concentrations, with the assumption that therapeutically relevant concentrations in the retina will in time be attained. Consequently, the time to effect is prolonged, and the substantial amounts of therapeutic delivered increases both off-target drug effects, and cost to the patient/public purse. Traditional regular intravitreal injection also comes with additional risks associated with piercing the ocular cavity, including: endophthalmitis, retinal and retinal pigment epithelial (RPE) detachment, retinal pigment tears, increased intraocular pressure, intraocular haemorrhage and anterior chamber inflammation 8. To mitigate these serious complications, in recent years there has been a significant push to develop novel methods of drug delivery into the eye. One such method that effectively overcomes the various internal and external barriers of the eye to aid in drug delivery relies on therapeutic ultrasound.

Ultrasound is referred to as any acoustic energy wave >20 kHz. It is typically used in medicine, both for diagnostic and therapeutic uses, facilitating non-invasive visualisation of internal tissues or tissue thermotherapy 9. Advantages associated with ultrasonic applications include its replicability, relative safety, low cost and selective targetability of tissue at different depths. Therapeutic ultrasound is a diverse field where applications range from gentle warming to manage soft tissue injuries, through to heat ablation applied in cancer therapy. Ultrasound has also been trialled as a non-invasive drug delivery method in dermal 10, brain 11, and ocular tissues 12-14. When considering drug delivery to the eye in-particular, ultrasound can improve the delivery of macromolecules across various ocular barriers. It has been used to permeabilise the BRB to improve intravenously administered viral delivery to the retina 15, disrupt corneal epithelium to enhance steroid transport into the anterior chamber 16, and deliver nanoparticles past the inner limiting membrane and into the retina after intravitreal injection 17.

Despite years-to-decades of *ex vivo* and* in vivo* research studies, ultrasound-mediated ocular drug delivery has never progressed beyond the 'concept phase.' The tool remains in its infancy, and literary reviews on the various methods of ultrasound-mediated ocular drug delivery have repeatedly highlighted the potential benefit this technology could provide in the areas of retinal gene therapy, needle-free transscleral drug delivery and improved oral delivery of therapeutics 12-14. The current scope of research appears targeted towards achieving both improved drug delivery and/or treatment outcomes; however, no single technology has yet been translated at the time of writing. A comprehensive systematic review investigating the research and development of ultrasound-mediated ocular drug delivery would provide a strong platform from which new, targeted research with the intention of near-term clinical translation could be borne.

To-date, to the best of our knowledge, there have been five published reviews examining the use of ultrasound to facilitate the delivery of drug to ocular structures. The first review in 2011 highlighted the need for further demonstration of sufficient and rapid drug delivery to target tissues at biologically relevant concentrations 9. Latter reviews focused on either ultrasound-mediated ocular delivery of specific agents, including gene delivery 13, or investigated specific drug delivery methods for which ultrasound is used, such as trans-topical delivery 12, and trans-BRB delivery 14. Most recently, Yang *et al*. (2022) published a review characterising some of the therapeutic benefits and safety concerns of different methods of ultrasound-mediated drug delivery 18. These reviews highlight both the potential for ultrasound-mediated drug delivery to improve therapeutics and the need for additional safety and tolerability data supporting translation to the clinic. However, despite these reviews exploring the modality, an assessment of the rigour of relevant studies, including reporting quality and risk of bias (RoB), was notably absent. Additionally, ultrasound - and the applied parameters thereof - for various applications have not been summarised and a defined roadmap of the efficacy and safety of ultrasound-mediated drug delivery required to move this technology to the clinic has never been synthesised. We have conducted a systematic review to consolidate the previous reviews, including additional studies up to December 2022. All published methods of ultrasound-mediated ocular drug delivery have been integrated, particularly in the context of their efficacy and safety characteristics. Study quality and RoB has been appraised using standardised tools, and a guide compiled highlighting the minimum study characteristics necessary to generate findings capable of aiding efficient clinical translation.

### 1.2. Objectives

A systematic review was conducted to map the application of ultrasound in ocular drug delivery. Key elements of the research questions expressed in terms of Population, Concept and Context (PCC) are described in Table 1. Additional focus was attributed to synthesising the results in the context of the parameters used, the impact on localised tissues, the current gaps in knowledge and potential future research avenues. The following primary research question was also formulated:

How is ultrasound used in ocular tissues to facilitate drug delivery, and what parameters and methods result in optimal outcomes in the context of improved safety, targetability and therapy?

Secondary research objectives included:

1. Are historically identified gaps in current knowledge being addressed over time?

2. What identified gaps in current knowledge still exist?

3. What are the current challenges in translation that are yet to be addressed?

4. How should future studies be designed to improve probability of translation between the lab bench and clinical application occurring?

## 2. Methods

### 2.1. Protocol and registration

The reporting of this review was guided by the Preferred Reporting Items for Systematic Reviews and Meta-Analyses (PRISMA) 19, and the protocol was registered on PROSPERO (CRD42022336854).

### 2.2. Eligibility criteria

Studies must relate to drug delivery intended for the eye, further, they should focus on ultrasound-mediated molecule delivery to ocular structures (*ex vivo* or *in vivo*). Peer-reviewed articles were included if published between 2002-2022 (inclusive) and were accessible in English.

Studies were excluded if they investigated drug discovery, or extra-ocular xenograft models of ocular diseases. In addition, studies assessing ultrasound-mediated molecule delivery only in *in vitro* cell lines were excluded. Reviews were excluded from the study analysis; however, they were used to inform advances in the field since publishing.

### 2.3. Information sources and search strategy

An initial review of the literature was conducted on 04/06/2022 to establish a baseline knowledge of ultrasound-mediated drug delivery to the eye. Information sources assessed included reviews published in 'Pharmaceutics' 14, and 'Expert Opinion on Drug Delivery' journals 12. The examined reviews were then used later to improve the search strategy using a “golden egg” approach, whereby the search strategy was refined until these reviews were included in the search results.

A second review of the literature was conducted on 09/06/2022 to identify and characterise suitable articles for the systematic review. The PCC framework (Table 1) was used to characterise the research question in a database search strategy and the final strategy used for each database is listed in Table S1. Importantly, the exclusion criteria “NOT *in vitro”* was not used in any database search, as this would remove eligible studies which assessed both *in vitro* and *in vivo/ex vivo* models, this exclusion criteria was therefore applied during screening. The following databases fwere searched: PubMed, EMBASE, CINAHL and SCOPUS. The search strategy was initially developed using PubMed and was later altered to suit the EMBASE, CINAHL and SCOPUS search engine parameters; all databases were searched on the same day. The search strategies were drafted by the primary author and further refined through team discussion. The final search results were exported into Endnote, then studies published before 2002 and duplicates were removed. The electronic database search was supplemented by examining grey literature, including the clinical trial database, clinicaltrials.gov. Directly prior to journal submission, the search strategy was repeated, and the review updated to include screening of an additional 34 unique papers, published between 09/06/2022 and 06/12/2022.

### 2.4. Selection of Sources of Evidence

Sources of evidence were selected from the identified literature based on their relevance to the inclusion criteria. A reviewer (IJR) sequentially screened each study title, then abstract, then full text for inclusion or exclusion. Where uncertainty was present, consensus was reached by consulting a second (HSP), or third (KRT) reviewer. Reasons for exclusion were recorded at each screening step and in the case of missing full-text articles, both the authors of the relevant papers were contacted, and the University of Queensland Document Delivery Service was used to attempt to source the missing full-texts.

### 2.5. Data Collection Process

A data-charting form was developed by first considering the studies based on their relevance to ocular delivery of drug. The data chart was reviewed by members of the team and subsequently updated in an iterative process. Two reviewers (IJR and LC) collected study characteristics and conducted bias and study quality assessments independently and in duplicate for all included studies. The resulting datasets were then compared and discrepancies in data collection and semi-quantitative analysis were highlighted and resolved after consensus was reached by the two reviewers, or by a third reviewer (HSP) if necessary.

### 2.6. Data items

Data was extracted from article characteristics including standard information (such as journal, authors, year published, type of publication, study objectives, outcomes and comparators, animal details and quantity). In addition, further characteristics pertaining to drug delivery including route of administration, *in vivo/ex vivo*, measured outcomes (method of uptake assessment, efficacy, agent administered), ultrasound parameters (transducer details, frequency, power or pressure, sonication duration, targeted location, duty cycle, pulse repetition frequency, pulse duration, number of sonications, inter-sonication interval, number of sonication treatments and inter-treatment interval), delivered microbubble details (route, brand, dose) and finally, safety assessment methods (techniques used and time of assessment post-treatment). Final study data characteristics are available in Table S2.

### 2.7. Risk of bias assessment

RoB was assessed using the Systematic Review Centre for Laboratory animal Experimentation (SYRCLE) RoB tool 20. The SYRCLE RoB assessment tool contains 10 domains, relating to 6 types of bias: selection, performance, detection, attrition, reporting and 'other' biases. The tool is functionalised through a set of pre-prepared questions designed by the tool authors. When using the tool, to assess low, high, or unclear risk of bias, it was indicated as “Yes,” “No,” or “Unclear,” respectively when answering these questions for each study. To assess the trends in bias of studies in this field, the proportion of “Yes,” “No,” and “Unclear” reporting items were aggregated, and the individual bias assessment of each study has been provided (Table S2).

### 2.8. Study reporting quality assessment

The quality of study reporting was assessed using the updated Animal Research: Reporting of *In Vivo* Experiments (ARRIVE 2.0) guidelines 21. These guidelines are made up of 21 reporting criteria, split into one group of 10, the essential set, and one group of 11, the recommended set. The essential set describes the basic minimum items that must be included in any animal research, without which readers and reviewers cannot assess the reliability of reported findings. Comparatively, the recommended set complements the essential 10 and adds important context to the described study. In this review, the ARRIVE 2.0 items were functionalised by the authors into a set of 38 questions: 22 questions describing the essential 10 items, and 16 questions encompassing the recommended set (Table S3). Each study was assessed either as “Yes” “No” or “N/A” for each question. To efficiently report the study quality assessment, a method of summarisation was adapted from a prior published systematic review 22. Briefly, each “Yes” response was ascribed 1 point, each “No” response was given 0 points, and each “N/A” response reduced the denominator by 1 point. A reporting quality co-efficient was defined (≥0.81 Excellent, 0.61-0.8 Average, 0.41-0.6 Poor and ≤0.4 insufficient) and given to each study by summing the points from each study and dividing by the denominator (total number of possible points minus the number of “N/A” responses). In addition, the proportion of “Yes”, “No”, and “N/A” items were calculated across each reporting criteria to assess trends in study reporting in this field. The reporting quality coefficient ranges were determined before study characterisation and not altered afterward in light of the results.

Both the RoB and study reporting quality of each study was assessed independently and in duplicate by two reviewers (IJR and LC). Inconsistencies in the results were resolved after reaching consensus through discussion, or by consulting a third reviewer (HSP). In addition to the summarised study coefficients and proportional reporting trends, the results of each study assessment were presented in their entirety as per PRISMA guidelines (Table S2).

### 2.9. Synthesis methods

Ultrasound may be used in a diverse array of applications to facilitate drug delivery to ocular structures. These methods rely on distinct, and sometimes overlapping, fundamental mechanisms to facilitate drug delivery, which may be impacted by both the animal model used and intended target of the administered drug. As such, studies were discussed based on the method of ultrasound delivery, the intended destination, and the animal models used. The intended destination sub-categories included: trans-topical delivery, blood-retinal barrier delivery and vitreal/vitreoretinal delivery. The method of ultrasound delivery subcategories included: exogenous microbubble barrier permeation (MBP_EXO_), acoustic streaming (AS), and endogenous microbubble barrier permeation (MBP_ENDO_). The animal model subcategories were differentiated based on the relative size of the treated eyes and included: small rodents (mice and rats), rabbits, and large mammals (pigs, cows, sheep, and goats). Quantitative meta-analysis could not be undertaken due to the significant heterogeneity of the study designs, outcomes measures, and interventions.

## 3. Results

### 3.1. Study selection and characteristics

**Figure 1:**
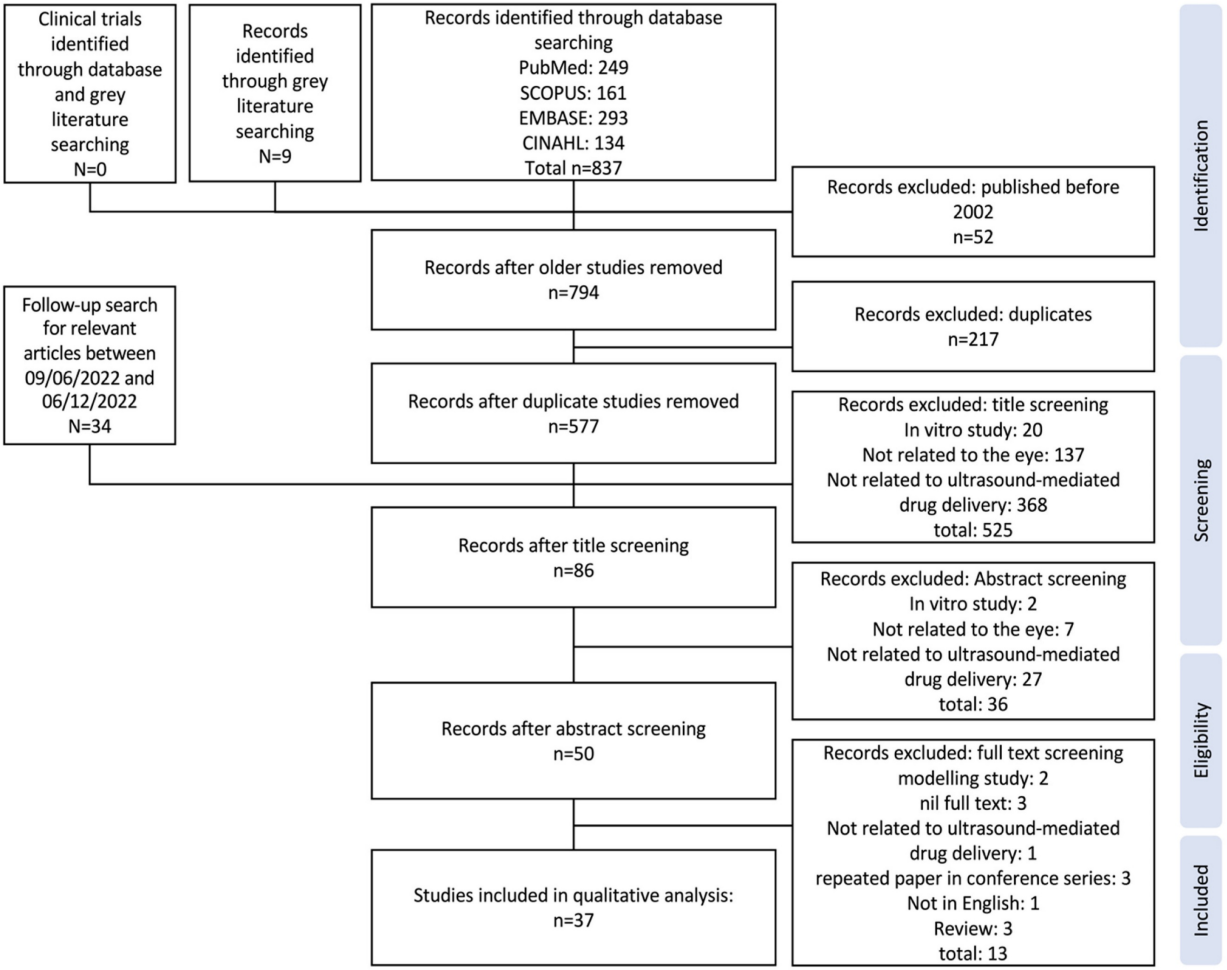
PRISMA flow chart describing the process of study selection from the applied database search strategy through to final studies included in qualitative analysis of review.

A total of 837 studies were identified from databases in the initial search on 09/06/2022, and a further 9 studies from grey literature searching. After removing older studies and duplicates, the titles of 577 studies were screened first, then the abstracts of the remaining 86 studies were assessed for eligibility. Finally, the full text of 50 seemingly eligible studies were examined, with thirteen studies subsequently removed, for reasons provided in Figure 1. Two modelling studies were removed due to a lack of *in vivo* or *ex vivo* data 23, 24, the full text of one study was not published in English 25, three papers were conference abstracts for which a full study was later published and included in this review 26-28, the full-text of three studies were not available 29-31, one study used an extra-ocular xenograft model of ocular disease 32, and finally, three identified publications were reviews 12-14. No clinical trials investigating any application of ultrasound-mediated ocular drug delivery were identified. An additional 34 studies were identified in the follow-up search for relevant articles published between 09/06/2022 and 06/12/2022, however all were removed during title screening. The study characteristics table comprising the study populations, parameters, results, bias, and reporting quality assessment can be found in Table S2.

### 3.2. Risk of bias: SYRCLE tool

In our analyses, all the studies displayed a significant or unclear RoB in four items: sequence generation, allocation concealment, blinding, and baseline characteristics. The median “high” or “unclear” RoB across all included studies was 7 out of the 10 criteria. The highest 'low' RoB score for any study was 4, with only 30% of the 37 included studies achieving this.

**Figure 2:**
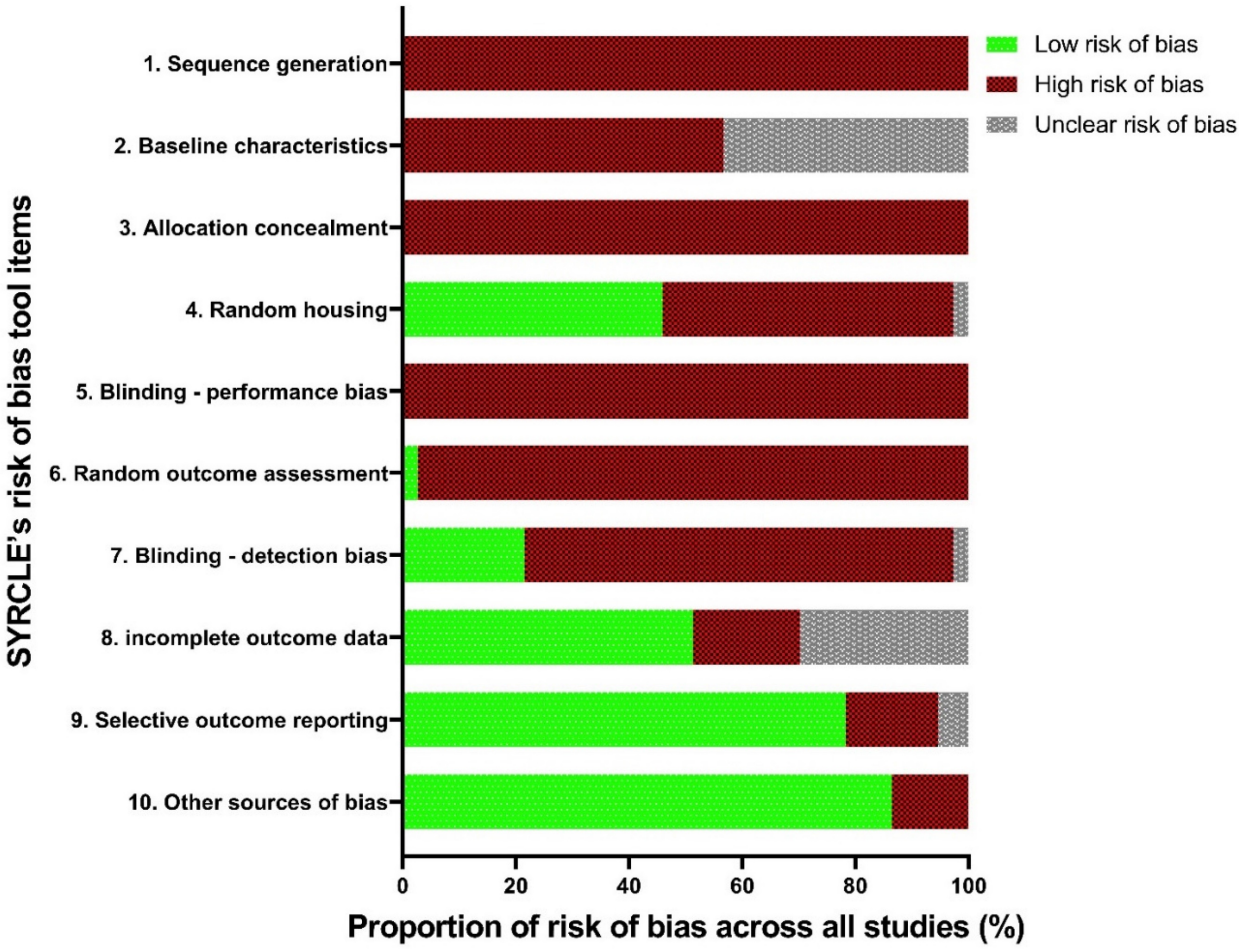
RoB assessment trends in the population of included studies, across SYRCLE's RoB tool items.

No study suitably randomised treatment and control groups or attempted to describe the characteristics of the treatment and control groups at baseline to ensure similarity between groups, significantly increasing the risk of selection bias (Figure 2). Only three studies randomised the order of outcome assessment, although these studies did not specify the method of randomisation 33-35. In addition, at no point were investigators blinded from knowing which intervention each animal received when allocating treatments or controls, or during the experiment, contributing to the risk of both selection bias and performance bias. Only one study reduced the risk of detection bias by randomising the order for assessing treatment or control outcomes 34. Whilst 46% of studies demonstrated a low risk of performance bias due to randomised housing, of these low risk studies, 88% achieved this by using *ex vivo* models, for which housing was irrelevant (Figure 2). A substantial proportion of studies (78%) consistently aligned their results to the described methods; however, nil published a protocol, therefore the viability of this factor cannot be confirmed; it is likely the published results were subject to survivorship bias, whereby only the successful results were published.

### 3.3. Study quality assessment: ARRIVE 2.0 tool

**Figure 3:**
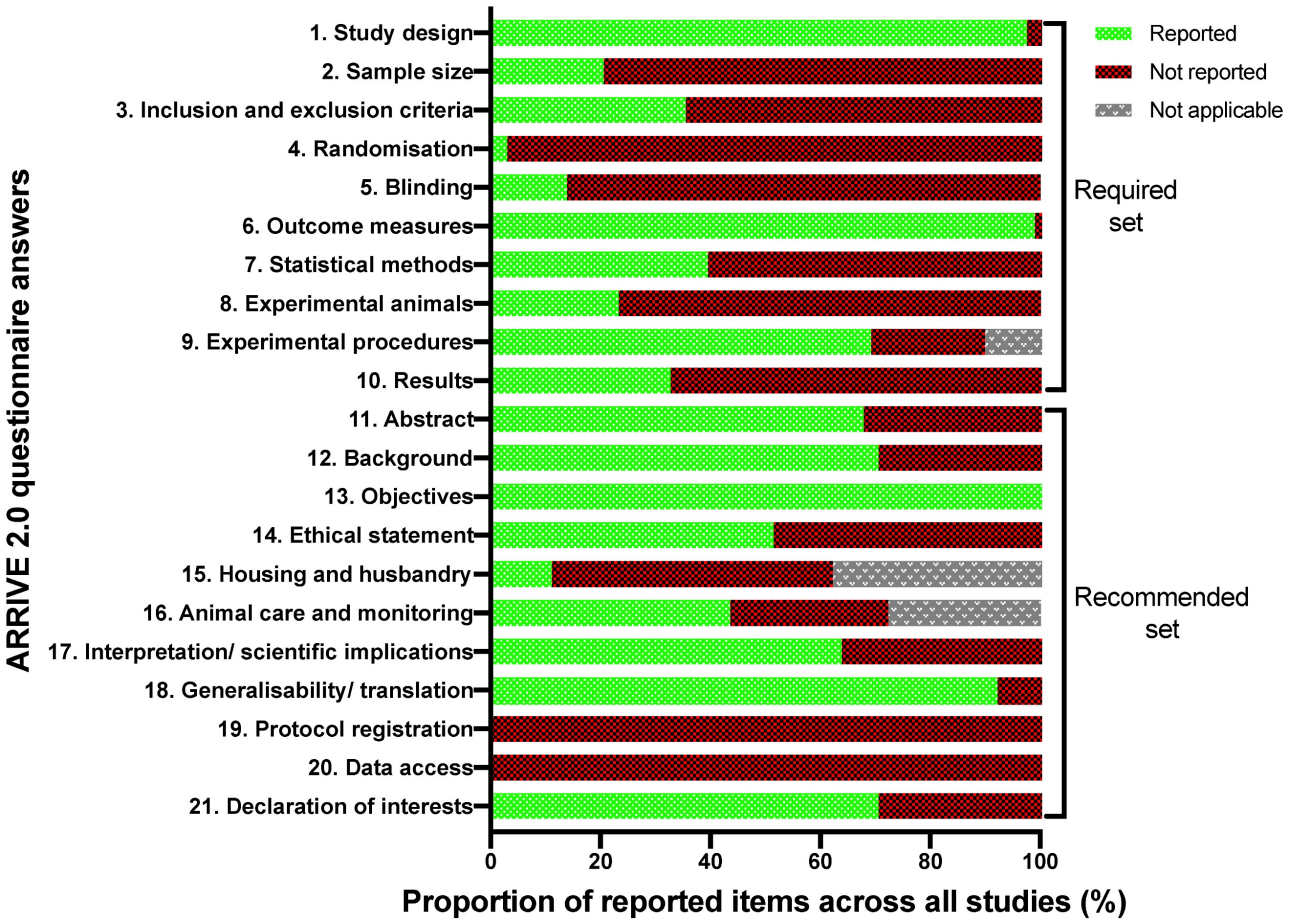
Study reporting quality assessment trends across the included studies mapped to the ARRIVE 2.0 reporting domains.

The summarised study reporting quality coefficients are shown in Table 2, with trends of each criterion appearing in Figure 3. The most prevalent areas of reporting failure in the required set of criteria included details relating to the total sample size and a justification for the sample sizes used.

No studies used inferential statistics to determine the minimum sample size required to have statistical power, and no studies justified the lack thereof. In addition, studies rarely used randomisation to determine treatment and control groups, and no studies described a reliable randomisation method, but rather claimed they 'randomised the allocation.' Only 5 studies reported awareness of the group allocation at various stages of the experiment, and all these studies only masked outcome observers, not the allocation nor delivery of treatments 28, 33, 36-43. In terms of the rigour of statistical methods, there were two aspects; inclusion of details of the statistical methods used and if methods were used to assess whether the data met the underlying assumptions of the statistical approach. In total, 28 studies included details around the statistical methods used, however only 6 of those studies used a secondary posteriori test to ensure the testing method used was appropriate 43, 44.

When considering the recommended set, four studies gave details pertaining to the housing and husbandry conditions 15, 45, 46. Availability of study data was stated in only one study, wherein the data was to be available upon request, however, throughout the review writing period the requested data was not supplied; as such, this study's data was considered not available 45. No studies registered their protocols prior to publishing. When considering the overall quality of the included studies, no studies showed excellent reporting quality, eight studies were of “average” quality, whilst twenty-four and five studies demonstrated 'poor' and 'insufficient reporting quality, respectively (Table 2).

### 3.4. Applications of ultrasound-mediated drug delivery in ophthalmic disease

The eye is an immune-privileged organ with structural features homologous to similarly privileged organs, including the brain and testes. The globe is also partially exposed to the external environment, which, whilst enabling topical drug administration, presents barriers to efficient trans-topical drug delivery. Traditional methods of drug delivery to the eye may be broadly divided into trans-topical application, intravitreal injection (vitreal/vitreoretinal), and systemic administration (trans-BRB), and the respective barriers relevant to efficient drug delivery will be discussed through the lenses of these delivery methods. The various barriers to delivery may be broadly categorised into static and dynamic types, each with their own specific biological and physiological challenges. These barriers impact the available methods by which ultrasound may be utilised for drug delivery to different eye regions. Whilst these methods are investigated extensively in latter chapters of this review, a brief introduction to the mechanisms by which ultrasound overcomes these barriers is presented here. In addition, the relevant diseases applicable to ultrasound-mediated ocular drug delivery are identified in the context of what has been experimentally attempted, and what has so far only been theorised.

#### 3.4.1. Barriers to trans-topical drug delivery

Trans-topical delivery involves the instillation of drops or ointments to the ocular surface. Using this route, therapeutics may be delivered to varying depths within the eye ranging from the superficial surface, in the case of keratoconjunctivitis, to the posterior segment in the adjunct treatment of posterior segment uveitis 47. As the therapeutic target progresses deeper into the eye, it becomes increasingly difficult to identify drugs capable of overcoming the static and dynamic barriers within the eye to reach this target. It is estimated that < 5% of the dose of most topically applied drugs are capable of reaching the anterior chamber and, in the case of posterior segment delivery, less than 1/10^8^ of topically administered protein drug reaches retinal therapeutic targets 48, 49.

Dynamic barriers located on the ocular surface include tear turnover and blinking, which act to minimise residence time of instilled formulations by either diluting instilled drug or mechanically clearing them from the ocular surface. The rate of tear film turnover, normally between 0.5 and 2.2 µL/min under normal conditions, increases after topical instillation. In addition, the eye reflexively blinks in response to the sudden increase in tear volume 48.

Static barriers are present at every stage of trans-topical ocular drug delivery. To start, the minimally exposed ocular surface acts to limit the usable instillation volume to ~20 µL. In addition, the presence of an oily layer, aqueous layer and negatively charged mucin layer within the tear film all inhibit interaction between instilled drug and the outermost cells of the eye 6, 48. Once the drug diffuses to the apical cell surface, tight junctions located between epithelial cells on the cornea and conjunctiva (covering the sclera) inhibit the paracellular transport of hydrophilic drugs. Inside the scleral stroma both the glycosaminoglycan matrix embedded with collagen and the high (80%) water content act as a barrier to lipophilic drugs 6, 50, 51.

After diffusion through the cornea delivered agents will be subject to accelerated clearance due to aqueous humour turnover. Whilst the anterior chamber provides useful targets for treating diseases such as glaucoma, drug residence time is stymied by continuous turnover of the aqueous humour. At a rate of 1.0-3.0 µL/min (slower at night), the entire volume of the aqueous humour is replaced within 2 hours 52, leaving little opportunity for continuous drug exposure to anterior segment tissues without inconvenient dosing intervals. Aqueous humour is produced in ciliary bodies of the posterior chamber, and following a pressure gradient migrates in a posteroanterior motion to the anterior chamber where it is primarily removed through uveoscleral outflow and the trabecular meshwork. Despite this, some minor proportion of the aqueous humour moves posteriorly into the posterior segment, over the inner limiting membrane (ILM), allowing minuscule amounts of topically delivered drugs to penetrate the posterior segment via the trans-corneal route 53.

In the context of trans-scleral drug movement, where the drug penetrates all layers of the sclera and reaches the choroid, an additional dynamic barrier is presented. Choroidal blood flow is considerable; comparable only to, and in some cases exceeding, blood flow through the kidneys 54. In common pharmacokinetic drug models for topical drug delivery, the choroid is usually regarded as a sink condition, in which the drug concentration is assumed to be zero 55. Thus, drug delivery to structures of the posterior segment, such as the retina and uvea often must rely on alternate and more invasive delivery methods.

Beyond increasing formulation concentration, brief punctal occlusion post instillation, and repeated dosing; common methods used to overcome barriers to topical delivery involve altered dosage form design and are targeted at improving retention time on the ocular surface, delaying clearance due to blinking, tear production and nasolacrimal drainage, and by improving penetration rates. These methods include the use of thicker eye drop bases such as ointments, in-conjunction with drug encapsulation within nanoparticles, which has been extensively reviewed elsewhere 48.

#### 3.4.2. Barriers to intravitreal drug delivery

Intravitreal drug delivery involves directly injecting drug into the posterior segment of the eye, normally at a site 3.5 mm posterior to the pars plana and into the mid-vitreous cavity. Highly concentrated drug bolus is relied upon to deliver therapeutics to retinal cell populations, driven by a concentration gradient. Whilst topical barriers to delivery are overcome, piercing the globe retains significant risk of adverse events, including vitreous haemorrhage, anterior chamber inflammation and retinal detachment 8. As such, these treatments are often reserved for sight-threatening diseases including AMD and ocular cancers.

Injected particles must diffuse through the hydrated cross-linked meshwork of collagen, proteoglycans and anionic hyaluronic acid that form the vitreous humour 56. Drug diffusion through this hydrogel is inhibited according to increasingly positive charge numbers, particle size and viscosity of the media, but is improved by increasing the initial bolus concentration and through saccadic motion of the eyes 57. Further complicating reliable delivery, as the eye ages, pockets within the vitreous progressively liquefy, and there is non-uniform hardening of the remaining gel matrix 56-58. Thus, sufficient drug delivery to the diseased portion of the retina may become less reliable depending on the location and extent of this liquefaction and hardening.

Drug will be cleared from the vitreous either anteriorly through the anterior chamber via the routes surrounding, but not through, the lens, or posteriorly, through the retina. Larger and more hydrophilic drugs are predisposed toward anterior clearance routes, as the continuous aqueous turnover forms a sustained sink condition. Hydrophobic drugs may also be cleared anteriorly through the generated concentration gradient; examples of drugs primarily cleared in this manner include the large and hydrophilic biologics, rituximab and bevacizumab, and the hydrophobic steroid, triamcinolone 58.

Once drug reaches the para-retinal space, it must then traverse the ILM, a collagen and glycosaminoglycan membrane that acts as a mechanical and electrostatic barrier, with pore sizes of approximately 10 nm and a net negative charge 7. Whilst biologics do not appear to be impeded by this membrane, larger and/or cationic moieties may be completely blocked by an intact ILM 7.

Overcoming these barriers to intravitreal delivery currently primarily relies on the delivery of regular, highly concentrated boluses, even for incredibly potent biologics such as aflibercept, which is a factor increasing the cost of biologic sight-saving therapy and may hinder access to treatment.

#### 3.4.3. Barriers to systemic drug delivery

Systemic drug delivery, primarily administered via the enteral route in the case of ocular drug delivery, is hindered by the presence of static barriers between the systemic circulation and the ocular parenchyma. A primary characteristic of the eyes facilitating their immune-privileged status is the presence of the BRB, which, in homology with the BBB, contains an inner endothelial cell lining to the microvasculature (BRB_i_), but, in contrast to the BBB, also contains an outer cell layer composed of the RPE (BRB_o_), both of which contain intercellular tight junctions 5. Tight junctions are a population of complex integral (e.g., Zonula occludens-1, -2, -3 (ZO-1, -2, -3) and cingulin) and peripheral (e.g., occludins, junctional adhesion molecules (JAM) and claudins) membrane proteins. The integral proteins anchor the transmembrane proteins, which extend into the paracellular space, binding adjacent cells together, and creating the seal against paracellular macromolecule movement characteristic of the tight junction 5. The BRB_i_ limits systemic drug delivery to the eye and maintains a diffusional barrier from the retinal-capillary blood supply. Based on a suite of studies reviewed by del Amo *et al.* 2017., the BRB_i_ appears to prevent the permeation of molecules of a diameter ≥ 2 nm, although transcellular movement of particles may be possible through passive diffusion and active transport 7. Comparatively, the BRB_o_, comprised of the RPE, which rests upon the underlying Bruch membrane, separates the neural retina from the adjacent fenestrated choroidal capillaries. This capillary network is extensive, providing the retina with blood at a rate of 696±110 mg/min throughout the whole choroid 5, 54. Whilst systemically administered, neutral drug moieties up to 500 kDa freely enter the choroidal parenchyma through these fenestrations, entry through the BRB_o_ is significantly limited based on the drug's physicochemical properties. Drug penetration is similarly limited above 2 nm in diameter, and in addition, the permeation of small molecules appears highly dependent on their lipophilicity; where the lipophilic drug betaxolol has demonstrated a trans-BRB permeability an order of magnitude higher than the similarly sized, but hydrophilic, carboxyfluorescein (10.3-16.7 x 10^-6^ vs 0.96-2.33 x 10^-6^ cm/s, respectively) 7. In addition, active transport mechanisms appear to play a dominant role in successful drug delivery to the retina from the systemic route. Drugs which display successful ocular delivery (either intended or serendipitously, in the case of adverse drug reactions) often are the substrates of influx transporters present on the RPE 7. Comparatively, two major efflux transporter populations, p-glycoproteins (p-gp) and multidrug resistance-associated proteins (MRPs) have been reported in human RPE, with the efflux directed into the choroid 55. These are highly efficient transporters with broad substrate specificity. Their combined expression allows the removal of large neutral (both p-gp and MRP transporters), anionic (MRP pumps) and cationic (p-gp transporters) molecules from the RPE 55.

Similarly, systemic drug delivery to the ciliary body and iris are limited by the presence of tight junctions within the anterior segment, which forms the blood-aqueous-barrier (BAB). Tight junctions are located on iris and ciliary muscle endothelial vasculature, and on the apical surface of the posterior iris, Schlemm's canal and non-pigmented epithelium 7. Since aqueous humour is secreted into the posterior chamber through non-pigmented epithelium, the BAB forms a barrier to systemic delivery of drugs targeting the anterior segment.

Methods used to overcome these systemic barriers to ocular drug delivery have historically relied on serendipitous drug discovery for moieties capable of either acting on drug targets accessible before the BRB or BAB, or by diffusing transcellularly, rather than paracellularly, to reach targets beyond the tight junction barrier. In addition, systemic drug delivery into the eye has been successful as a result of intentional drug design of neurological agents, where drug aspects promoting trans-BBB delivery also improve trans-BRB/BAB penetration due to structural homology within these barriers 58.

In the case of drugs used for glaucoma, where the site of action is commonly within the ciliary processes, highly lipophilic drugs such as betaxolol are capable of diffusing into the ciliary processes (and retina) via transcellular pathways 59. Comparatively, in the context of transporter homology between the BBB and BRB/BAB, a classic example includes the administration of L-DOPA, an amino acid precursor of dopamine, used in the treatment of movement disorders such as Parkinson's disease. L-DOPA is recognised- and transported- by the L-type amino acid transporter 1 (LAT1), a transporter present both on the BBB and BRB. As a result, the ocular symptoms of Parkinson's disease, including blurred vision or impaired convergence, may be effectively treated by systemic L-DOPA administration 58. Additional methods of drug physicochemical and transporter-related drug delivery to ocular structures are reviewed elsewhere 59.

#### 3.4.4. Ultrasound as an adjunct in overcoming barriers to traditional ocular delivery

An in-depth exploration of ultrasound physics, including how their interactions with ocular biology and cavitation nuclei have been used to facilitate drug delivery is located in section 3.5. As a prelude, describing how ultrasound has been used to overcome the aforementioned static and dynamic barriers is useful in highlighting its utility in ocular drug delivery.

Cavitation, either of dissolved gases endogenous to tissue, or of exogenously delivered microbubbles, is key to improving all aspects of ultrasound-mediated ocular drug delivery.

In trans-topical drug delivery, ultrasound creates cavitation nuclei from dissolved gases within the coupling media, which, at low acoustic pressures acts to “loosen” or create gaps in tight junctions of epithelium and, at higher pressures, appears to broadly disrupt cellular organisation in the upper epithelial cell layers 43, 60, 61. In addition, there appear to be underlying physiological responses controlling tight junction mechanics in response to ultrasound. In one study assessing the effect of ultrasound on corneal epithelium, low acoustic pressures reduced the expression of occludin, a transmembrane tight junction protein, and ZO-1, the binding protein anchoring occludin to the cell cytoskeleton, whilst higher pressures upregulated ZO-1 and further decreased occludin expression 43. Importantly, these changes appear reversible, and the epithelium has been shown to be capable of healing within 90 minutes to 6 hours 62. In addition, the acoustic wave induces bulk flow in the drug-filled coupling medium, termed acoustic streaming, which may act to actively deliver therapeutics into contact with, and through the topical surfaces 45, 49. Finally, thermal effects associated with tissue absorption of acoustic energy have been postulated to improve transcorneal drug delivery, however strict safety guidelines proposed by the United States Food and Drug Administration (US-FDA) limit applicability 16, 63.

For intravitreal delivery, ultrasound has primarily been used to improve the targetability of injected therapeutics through acoustic streaming. Whilst effective nanoparticle-loaded drug delivery through the vitreous and into the retina has been demonstrated 17, 40, 64, the inclusion of gas into the nanoparticle vehicle, as is the case for echogenic liposomes (ELIPs), has been particularly efficient as the gas within the ELIPs acts as a 'sail' with which the acoustic field interacts to propel the liposomes toward the targeted retina 42. In addition to acoustic streaming, ultrasound appears to improve the permeability of the ILM and promote drug delivery into the neural retina and RPE from the vitreous 40, 65.

For systemic delivery, ultrasound-mediated drug delivery to the eye has primarily mimicked similar efforts relating to trans-BBB drug delivery; using exogenous microbubbles as cavitation nuclei to open tight junctions and facilitate drug diffusion into the immune-privileged organ. In this aspect, drug is co-administered with microbubbles, which travel to the vascular bed of the tissue of interest targeted by ultrasound. As microbubbles reach the sonicated vascular bed, cavitation activity 'loosens' the intercellular tight junctions, allowing for targeted opening of the BRB, and diffusion of drug into the retinal parenchyma 15. This BRB opening appears reversible, with barrier function restored within 3 h post sonication 66. Additional theoretical mechanisms of improved retinal drug delivery stem from research in the BBB, where it has been shown that cavitation upregulates the internalisation pathways for the delivery of larger moieties 67, 68. The investigation of this interaction in BRB populations would be of benefit to furthering our understanding of mechanisms behind ultrasound-mediated ocular drug delivery of systemically administered therapeutics.

#### 3.4.5. Disease specific application of ultrasound in ocular drug delivery

Topical application of ultrasound to facilitate improved drug delivery are either focused on delivery into the cornea and anterior segment, or into and through the sclera for improved posterior segment delivery and have to-date only been evaluated pre-clinically. Whilst most studies have aimed to improve the delivery of poorly penetrable drugs, others focused on enabling the use of lower doses to reduce side effects with similar treatment efficacy. Gatifloxacin has been delivered into the cornea for bacterial keratitis or post-operative infection prophylaxis 45, various beta blockers (atenolol, carteolol, timolol and betaxolol), used in glaucoma, have had their corneal permeability increased when coupled with ultrasound 69, and dexamethasone sodium phosphate has been successfully delivered to the aqueous humour with the intention of reducing the dose (and therefore adverse effects) for managing inflammatory conditions of the anterior segment 16, 70. In addition, work has been completed to improve intracorneal delivery of riboflavin for collagen crosslinking in treating keratoconus and corneal ectasias 43, 71.

Regarding posterior segment delivery via the topical route, current studies have primarily relied on drug mimics and fluorophores of assorted sizes to demonstrate needle-free posterior segment delivery through ultrasound application. Mimics including sodium fluorescein 41, rhodamine 6G 49, dextrans ranging from 20 kDa to 150 kDa 39, 72, and bovine serum albumin 61, 73, 74, have all demonstrated improved delivery either into the sclera or through to the posterior segment through ultrasound application. These studies focused on demonstrating proof of concept, with the eventual intention of delivering a needle-free modality for drug delivery into the vitreous, for diseases such as endophthalmitis, and into the retina, for diseases including AMD and DRE.

When considering intravitreal delivery, studies have primarily investigated ultrasound as a modality for improved protein and gene delivery into the retina and RPE. Treatments have included both the gene and protein vectors of pigment epithelium-derived factor (PEDF) to inhibit choroidal neovascularisation (CNV) 75, 76, whilst others have transfected RPE with growth factor-β2 (GF-β2) and platelet-derived growth factor-B (PDGF-B) plasmids to attenuate proliferative vitreoretinopathy 34. Another study examined the efficacy of mouse nerve growth factor (MNGF) delivered to the retina to ameliorate hypertension-induced neuroretina damage 46.

Trans-BRB delivery applications are also primarily targeted toward improving the treatment of retinal diseases. The trans-BRB delivery of plasmids encoding wild type p53 and Rb94 induced apoptosis of intraretinal retinoblastoma xenografts 44, 77, whilst immunoliposomes loaded with PEDF protein successfully inhibited progression of CNV over the course of a week, requiring daily systemic injection followed by ocular sonication 76.

### 3.5. Foundational mechanisms facilitating ultrasound-mediated ocular drug delivery

**Figure 4:**
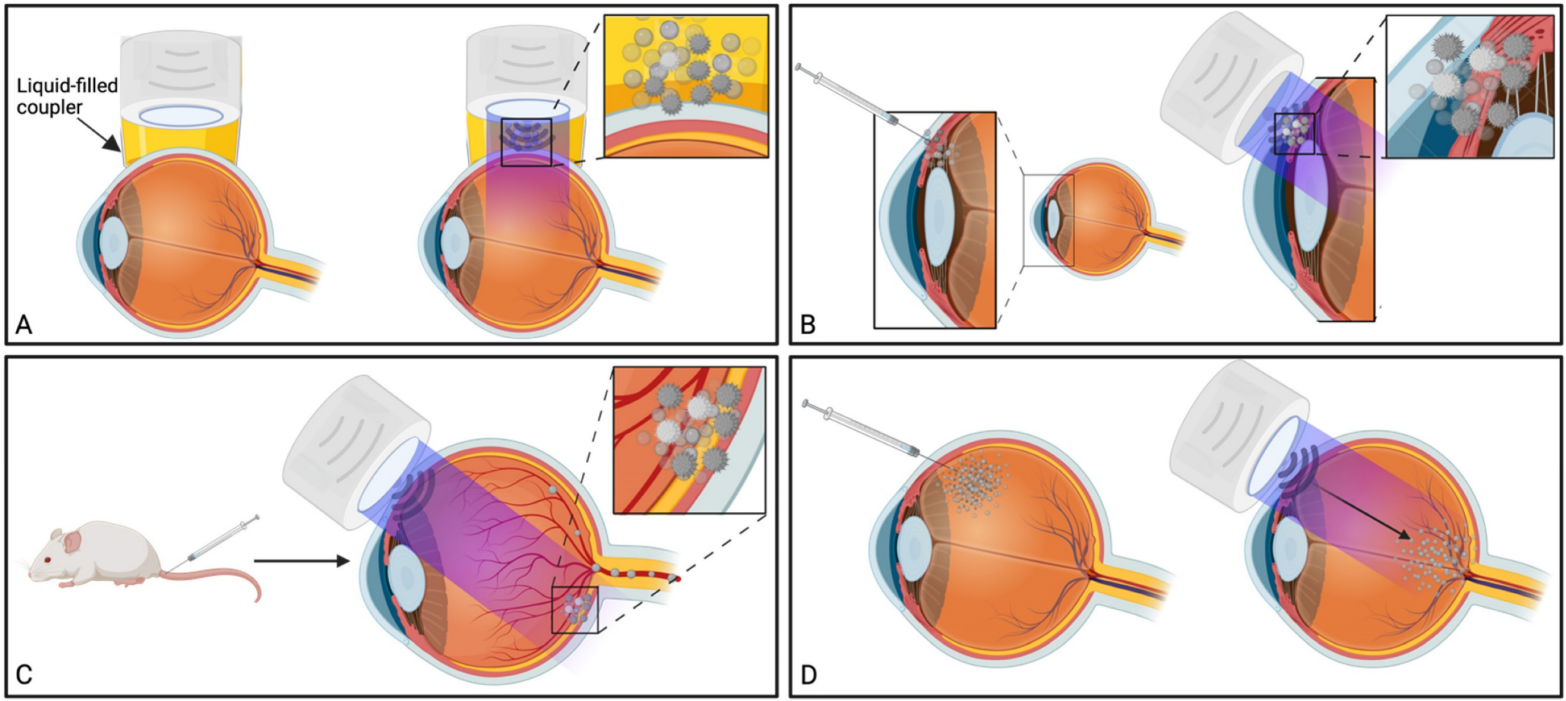
Summary of ultrasound-mediated drug delivery routes and their common underlying mechanisms. **A)** Trans-topical delivery primarily relies upon cavitation nuclei sourced from dissolved gasses in the drug-filled coupler (MBP_ENDO_); **B)** Direct injection of drug into target tissue and **C)** trans-BRB delivery use coadministered microbubbles as the source of cavitation (MBP_EXO_); **C)** AS facilitates drug delivery in the direction of applied ultrasound, either through liquid movement or via streaming of exogenously delivered microbubbles and is most clearly demonstrated by vitreal/vitreoretinal routes of drug delivery. Created using BioRender.com

Ultrasound has been used in multiple distinct ways to improve ocular drug delivery. The method of categorisation used in this study was dependent on a combination of the underlying mechanisms facilitating drug delivery, and whether an exogenous cavitation source was co-administered. Exogenous microbubble barrier permeation (MBP_EXO_) and endogenous microbubble barrier permeation (MBP_ENDO_) both rely on ultrasound-induced cavitation of microbubbles to improve drug delivery. In this review they have been differentiated by MBP_EXO_ requiring exogenous microbubble administration into the sonicated location, whilst MBP_ENDO_ relies on the generation of cavitation nuclei from dissolved gasses within the ultrasound field. The underlying cavitation activity differentiating these categories is not always clear, and may overlap, but they tend to have distinct ultrasound parameters and routes of administration due to the differing physicochemical characteristics between artificially engineered microbubbles and those which are produced from dissolved sources. Comparatively, the third category, acoustic streaming (AS), involves ultrasound-induced forces physically distinct from both MBP_EXO_ and MBP_ENDO_, whereby differences in ultrasound interactions between highly echogenic microbubbles and the less echogenic surrounds results in bubble acceleration in the wave propagation direction (figure 4). These differing mechanisms lend themselves toward distinct routes of delivery; Trans-topical applications primarily rely on MPB_ENDO_-mediated effects, whilst direct injection into target tissue and trans-BRB applications typically use exogenous microbubbles for cavitation effects. Acoustic streaming has most clearly been demonstrated in vitreal/vitreoretinal uses but may contribute to improved molecule delivery in the prior outlined routes as well (Figure 4). A characterisation of study delivery methods, route of administration, animal models and their assessments are included in Table 3.

#### 3.5.1. Exogenous microbubble barrier permeation (MBP_EXO_)

**Figure 5:**
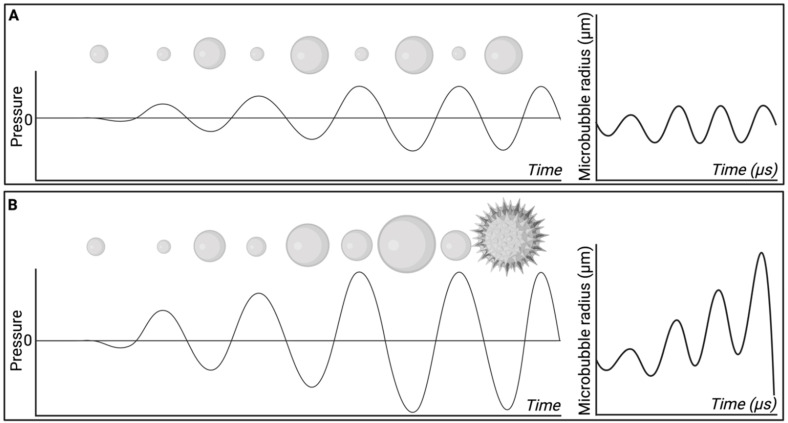
Microbubble behaviour at low and high sonication pressures. **A)** At low sonication pressures, or high frequencies, microbubbles tend to undergo stable cavitation whereby the bubble undergoes cyclical expansion and contraction. **B)** microbubbles influenced by high sonication pressures, or low frequencies, may undergo inertial cavitation, whereby during rarefaction microbubble size progressively increases until a critical size is reached, and the bubble undergoes implosion. Created using BioRender.com

Particularly relevant for targeted delivery of therapeutics across biological barriers, MBP_EXO_ relies on a combination of microbubble administration followed by ultrasound of relatively high peak negative pressures (PNPs) to improve drug delivery in a time- and location-dependent manner 78. When acted upon by an acoustic wave of relevant frequency and PNP, microbubbles expand and contract in an oscillatory motion, a phenomenon termed cavitation, which efficiently translates the applied acoustic pressure into more direct mechanical forces on the surrounds 79. At lower pressures, or higher frequencies, microbubbles undergo stable cavitation, whereby their repeated cycles of expansion and contraction impart reversible changes to the surrounding vasculature. Comparatively, at higher pressures or lower frequencies, the expansion and contraction of microbubbles becomes unstable, resulting in progressively larger oscillations and eventual collapse (Figure 5). This creates microjets of compressed gas which pierce surrounding tissue, increasing the risk of damage to the local parenchyma and vasculature 80.

Traditional applications of MBP_EXO_, such as BBB-targeted drug delivery, rely on injected microbubbles reaching the vascular beds of the brain parenchyma, which is sonicated in order to mechanically “loosen” the inter-endothelial cell tight junction proteins, such as claudins and occludins, allowing the co-delivered drug to passively diffuse into the parenchyma via paracellular movement 11. Additionally, cavitation has been shown to upregulate clathrin- and caveolin-mediated transcytosis and endocytosis pathways, particularly for larger, ~500 kDa sized particle delivery 67, 68. Advances in the application of this method have included using drug-filled microbubbles to minimise off-target toxicity and/or maximise localised drug delivery during microbubble destruction, a particularly useful tool in cancer chemotherapy 81. Alternatively, co-administering microbubbles with drug-loaded nanoparticles or viral loads with cell-specific promoters also shows promise in targeted treatment 79. In ocular drug delivery, studies have investigated the effect of coadministering microbubbles and drug either by direct injection into the target tissue, by intravenous injection using a readily accessible peripheral vein, or by comparing both methods (Figure 4 B, C).

BRB permeation using MBP_EXO_ relies on bubble cavitation for permeation, inducing intercellular tight junction separation, and the upregulation of transcytosis pathways. Trans-BRB delivery of PEDF has effectively been used to treat CNV in rat models 76. Importantly, almost all studies associated with blood-retinal barrier opening have used planar transducers to open the BRB of small rodents, where the distance between transducer to target is minimised by the relatively small eye dimensions (*c.f.* human eye), allowing for lower sonication pressures to be used. This contrasts with BBB delivery whereby it is common to use a focused ultrasound transducer which concentrates the ultrasound beam on the target tissue at a desired depth, improving ultrasound delivery through the skull and minimising the impact to the tissue in the beam path 11. One included study by Touahri *et al*. (2020) used focused ultrasound to open the BRB in rats, demonstrating effective delivery of Evans blue and an adeno-associated virus, with minimal retinal damage 15. Future BRB-opening studies in larger mammals and humans may benefit from the use of HIFU as this would allow for ocular structures in the beam path to be exposed to lesser intensities of ultrasound, improving thermal and mechanical side effect profiles in these sensitive tissues.

Most of the MBP_EXO_-studies directly injected a combination of microbubbles and ligand into the target tissue, with a substantial proportion delivering genetic material with the intention for transfection (Table 3). Injected locations included the conjunctiva 37, ciliary muscle 28, retina 35, 82, 83, and into vitreous-invading retinoblastoma xenografts 44, 77. All these studies displayed improved efficacy when combining microbubbles and ultrasound, compared to ultrasound alone at the applied parameters. Importantly, however, no study confirmed the presence of inertial or stable cavitation using a passive cavitation detector (PCD), thus it is not possible to reliably attribute the mechanism of improved delivery to stable cavitation, or microjet formation in the event of bubble collapse. Since stable cavitation is inherently less damaging to tissues than inertial cavitation, and requires less power at a given frequency, the addition of a PCD to experimental design would greatly improve investigations into the specific mechanism underlying transfection in these applications, improving study quality.

#### 3.5.2. Endogenous microbubble barrier permeation (MBP_ENDO_)

MBP_ENDO_ encapsulates the use of ultrasound to improve drug delivery without delivering exogenous microbubbles. Cavitation activity therefore relies on the chosen sonication parameters promote the growth of cavitation nuclei from dissolved gases within the coupling media or tissue being sonicated. As for MBP_EXO_, these cavitating microbubbles will act to translate the acoustic energy into mechanical and thermal energy, physically altering the surrounding tissue to create 'openings' and promote drug delivery 84, 85.

In ocular drug delivery, most studies use MBP_ENDO_ to deliver topically applied drug into or through the ocular surface (delivery pathways indicated in Table 3). This method has been used to deliver drug to both the anterior and posterior eye segments and represents a greatly improved delivery technique over eye drops, for which on average less than 5% of the applied dose is successfully delivered 48. Importantly, improved drug delivery to the posterior segment may help facilitate the obsolescence, and ultimately, retirement of intravitreal injections from medical practice. Intravitreal injections are inherently damaging to the eye in a multitude of ways, as described earlier 8, and their removal from practice may lower the barriers toward accessing sight saving therapy particularly in rural and remote regions. Despite the potential advantages of transscleral drug delivery, there are still significant hurdles before translation to clinical practice is achieved. These hurdles range from optimising ultrasound parameters to improve efficacy whilst minimising localised tissue damage, to reducing the time required for drug formulations to remain in contact with the eyes' surface post sonication.

The first study investigating transscleral delivery explored in this review applied continuous ultrasound to rabbit eyes for 60 minutes which, whilst effectively improving the delivery of a myriad range of drugs, is not a clinically feasible treatment duration 69. Since that study was published, sonication times have significantly reduced, with multiple studies using a shorter, 5- or 10-minute sonication time, followed by 60 minutes of soaking 41, 60, 70. Further studies have reduced the soaking time significantly too, toward 5 minutes (Table 3, ref 16, 62). This trend in faster ultrasound-mediated drug delivery has progressed this technique toward a viable treatment duration for widespread use, although no clinical trials or human application has been recorded to-date.

Four studies employing MBP_ENDO_-mediated drug delivery used PCDs to assess the presence of cavitation during treatment using different ultrasound parameters (Table 3, ref 61, 62, 72, 73). Passive cavitation detection plays a key role in ultrasound-mediated drug delivery, as it allows for improved characterisation of the forces being applied to tissue. When inertial cavitation occurs, the damage to localised tissue is often greater in magnitude compared to stable cavitation, due to the creation of shock waves and microjets which impart greater peak mechanical forces on the surrounding tissue, damaging cells, and vasculature 80. Bubble collapse also reduces the concentration of bubbles in the solution over time, whereas with stable cavitation the bubbles remain *in situ* for longer, and the mechanical forces may be applied throughout the entire duration of sonication. The relationship between cavitation and ultrasound frequency and PNP is investigated in section 3.6.3.

#### 3.5.3. Acoustic streaming

**Figure 6:**
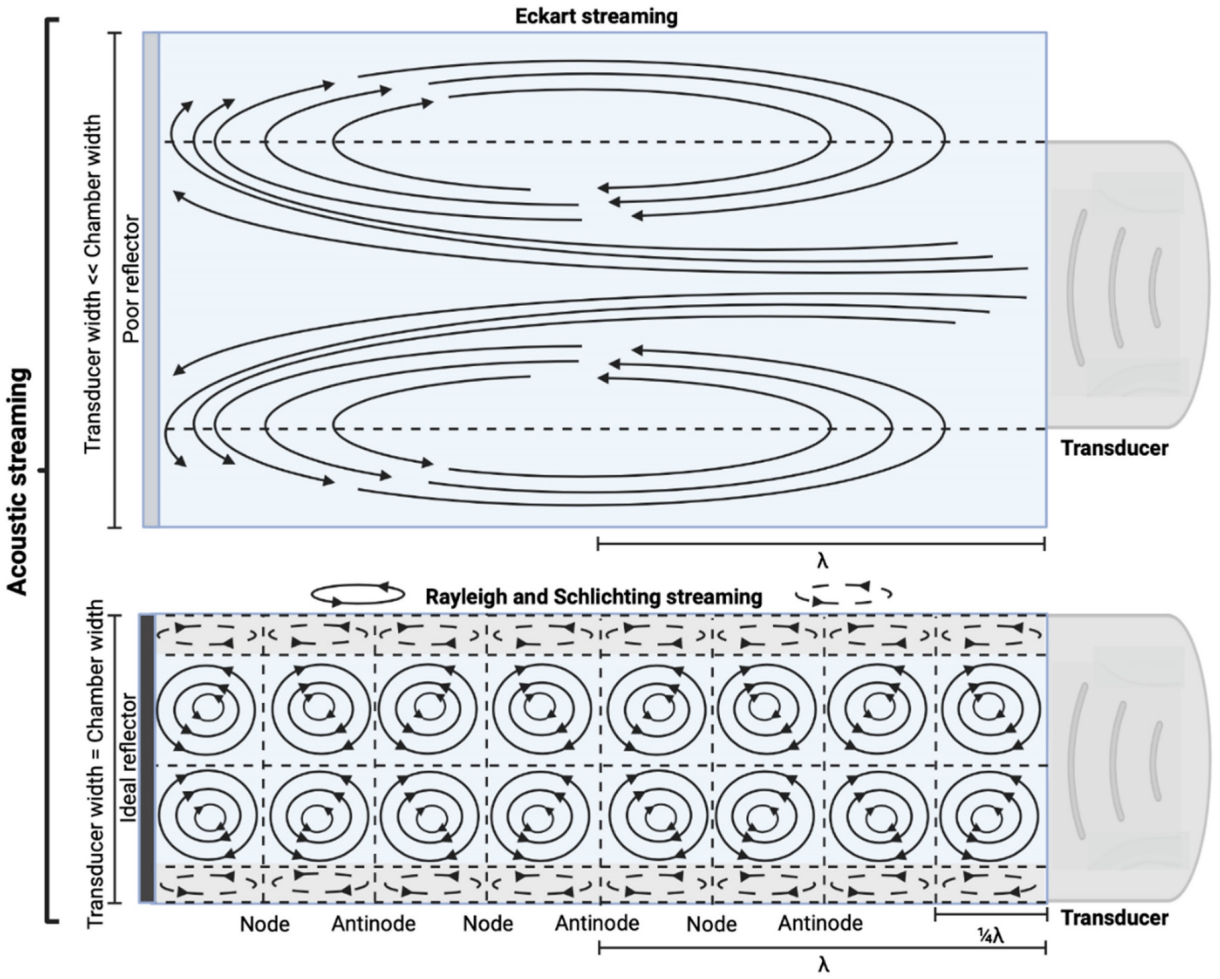
Comparison of the various streaming behaviours in fluid medium. Eckart streaming is caused by vessel diameters greater in size to the transducer diameter, whereas Rayleigh and Schlichting streaming occur in vessels where the transducer has a similar surface diameter (c.f. vessel). Created using BioRender.com.

AS describes the flow generated by an ultrasound wave through a fluid medium, in the direction of applied ultrasound 86. In the drug delivery context, this process has been shown to deliver drug within the beam path deeper into tissues than what would be expected from MBP_ENDO_-mediated mechanisms alone 61, 62, 69. Two of the primary physical properties contributing to this streaming behaviour include Eckart and Rayleigh streaming, each of which contribute to bulk streaming depending on the environmental characteristics of the sonicated tissue and the ultrasound apparatus (the contribution of Schlichting streaming, which relates to vortices formed against the vessel walls, is considered negligible in this instance) 87. For applications where the sonicated environment is larger than the transducer diameter, and/or where there is incomplete reflection of the ultrasound wave from the tissue surface, Eckart streaming dominates. This streaming is characterised by a scale far greater than the ultrasound wavelength, whereby a central stream traveling in the wave direction is surrounded by an opposing stream column outside the transducer area (Figure 6) 88. Comparatively, Rayleigh streaming occurs in environments where the sonication cavity is similar in diameter relative to the transducer, and/or the sonicated tissue forms an ideal reflector of the ultrasound wave. The interaction between the drag of the vessel wall and the velocity of the streaming medium creates vortices in the beam path approximately one quarter the size of the incident wavelength (Figure 6) 89.

Whilst the method for determining the relative contribution ultrasound-induced Eckart and Rayleigh streaming has been thoroughly characterised elsewhere 86, understanding what contributes to the velocity of a streaming fluid is more immediately relevant to drug delivery, and may be described by the following set of equations 90:


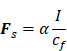

(1)

where 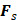 is the driving force of acoustic streaming per unit volume, 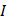 is the acoustic intensity of the transducer, 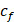 is the speed of sound in the fluid medium and 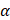 is the attenuation coefficient of the fluid.

In addition, acoustic intensity may be described as:


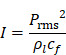

(2)

where *P_rms_* represent the root-mean-square (rms) pressure and 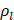 is the density of the liquid.

The attenuation coefficient 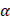 of the fluid medium will often be provided, but may be calculated by 90:


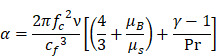

(3)

where 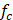 is the centre frequency of ultrasound, 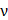 is the kinematic viscosity coefficient, 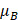 is the bulk viscosity coefficient, 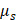 is the shear viscosity coefficient, 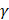 is the ratio of specific heats and 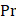 is the Prandtl number describing the ratio of kinematic viscosity to thermal diffusivity.

Finally, the acoustic pressure for a sinusoidal waveform may be described as:


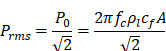

(4)

where 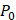 is the acoustic pressure amplitude and 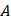 is the vibration amplitude of the ultrasonic transducer.

Therefore, the relative acoustic force accelerating the fluid stream in the beam path will be proportional to 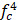 and 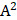, and, given a constant frequency, density, and speed of sound in liquid, pressure will increase proportionally to the vibration amplitude of the transducer. Thus, the two primary controllable factors determining acoustic streaming forces include frequency and sonication pressure.

Finally, when considering specifically the streaming of a medium where the bulk viscosity of the liquid medium does not contribute to the ultrasonic absorption coefficient, acoustic streaming velocity may be calculated using the following equation 91:


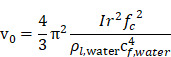

(5)

where 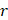 is the radius of the ultrasound beam. This has been used to estimate the contribution of acoustic streaming toward improved drug delivery in a trans-topical application, where a water bath containing various drug mimics was used to couple an ultrasound transducer to rabbit sclera 72.

In ocular drug delivery, it is a challenge to entirely separate the contribution of AS from cavitation caused by MBP_ENDO_ or MBP_EXO_ for barrier permeation. Trans-topical studies often investigate increased permeability of the ocular surface and attribute any increase in penetration above what would be expected from passive diffusion to the effect of AS & MBP_ENDO_. Two methods have been used in trans-topical drug delivery to attempt to separate these contributing factors:

1. Tissue was sonicated whilst the drug was present in the coupling media, and the magnitude of drug delivery was compared to tissue where drug was added only after sonication.

2. The magnitude of drug delivery after sonication and immediate snap freezing of the tissue was compared with tissue that was sonicated and allowed to soak in the coupling media.

In the first instance, two studies showed coincubation of drug during sonication achieved higher transscleral penetration when using long (60 minute) and short (5 minute) sonication duration (SD) and coincubation times 62, 69. In the second instance, immediate snap freezing revealed a reduced penetration of FITC-labelled bovine serum albumin (67 kDa), after 30 second sonication, when compared to a 30 second sonication plus 15-minute coincubation. No sham ultrasound control was used 61. The studies support the hypothesis that AS contributes to the magnitude of trans-topical drug delivery.

Comparatively, another study used the snap freeze method to show that the depth of penetration caused by acoustic streaming was proportional to decreasing frequency, and inversely proportional to increasing molecular weight. Chau *et al*. (2017) assessed dextran penetration through *ex vivo* rabbit sclera and found that after 30 seconds of sonication and subsequent snap-freezing, 20 kDa dextran penetrated 20.42, 9.20, 7.99 and 5.90-fold deeper compared to non-sonicated controls at frequencies of 40 kHz, 500 kHz, 1MHz and 3 MHz, respectively. Whilst 70 kDa dextran only demonstrated an improvement to penetration distance at 20 kHz and 500 kHz, at 10.66 and 3.88-fold improvement. These results were compared to samples sonicated and allowed to soak for 15 minutes in coupling media, where the relative improvement in penetration distance compared to a nil-sonication control was 2.97, 3.02, 2.31 and 1.94-fold improved for 70 kDa dextran. 20 kDa dextran penetrated the entire sclera at all sonication parameters after the 15-minute coincubation and were unable to be compared 72. The authors also modelled the expected streaming velocity of the coupling medium under different frequencies and found the velocity induced by the most effective frequency, 20 kHz, should be negligible, at 7.81 x 10^-6^ cm/s, whilst the highest frequency would have achieved a non-negligible 4.39 x 10^-2^ cm/s velocity, moving a particle ≈ 1.32 cm during 30 seconds of sonication. The authors suggested that rather than linear AS forces delivering dextran into the sclera, the flow induced by microstreaming at the boundary layer of cavitating nuclei in the coupling media likely contributed to the improved penetration. Since the magnitude of cavitation activity is inversely proportional to sonication frequency, this theory may be a viable explanation. Another aspect to consider includes the behaviour of cavitation nuclei within ultrasonic fields, whereby cavitating gas is propelled with high efficiency in the direction of the acoustic wave, thus the streaming velocity of the coupling medium may be higher than what is predicted by the applied models. This is a key mechanism underlying vitreal/vitreoretinal routes of ultrasound-mediated drug delivery.

AS has been investigated in combination with echogenic dye-loaded microbubbles delivered intravitreally into *ex vivo* porcine eyes 42. When adding a population of microbubbles to a solution under the influence of an ultrasound wave, additional secondary forces become apparent. Specifically, secondary radiation forces, such the secondary Bjerknes forces cause the microbubble population to cluster, then accelerate under the present Eckart and Rayleigh forces 92. Similar to the driving parameters influencing streaming force, 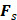, the velocity of microbubbles in a solution will be primarily determined by the viscosity of the solution and the frequency and pressure output of the ultrasound transducer, as described by the following equations 93:


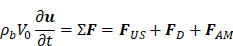

(6)

where 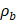 is the density of the gas core, 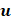 is microbubble velocity and 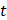 is time, 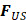 is the ultrasound radiation force, 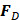 is the drag force, 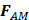 is the “added mass” force, 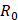 is the bubble radius at equilibrium, and 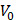 is the initial bubble volume described by:


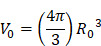

(7)

If we ignore secondary radiation forces, which will group bubbles in the initial seconds of sonication 92, and ignore the buoyancy force on the bubble, which is orders of magnitude smaller compared to other forces acting on the microbubble 93, the individual forces acting on the bubble may be calculated as follows:


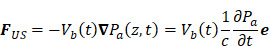

(8)

where the bubble volume, 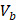, at any given time is:


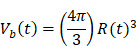

(9)

And the local pressure gradient, 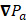, at a particular time and location from the transducer is:


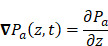

(10)

where 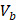 is the bubble volume, 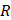 is the bubble radius, 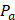 is the acoustic driving pressure, and 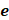 represents the unit vector in the direction of the ultrasound wave propagation. The drag forces due to the viscosity of the solution is expressed as:


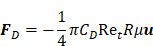

(11)

where


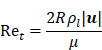

(12)

and


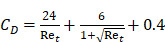


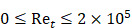

(13)

where 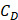 is the drag coefficient of a sphere, 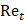 is the translational Reynolds number and 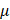 is the liquid viscosity. It should be noted that this model for finding the drag coefficient is accurate within ±10% where 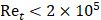, above which the boundary layer on the sphere becomes turbulent and drag markedly drops. Drag is also assumed to be quasisteady and only dependent on the instantaneous bubble radius and velocity 94. Therefore, the model is primarily relevant in viscous mediums, like those seen within the vitreous.

The force due to added mass is incorporated to represent the effect of the bubble accelerating and decelerating through the surrounding liquid, adding inertia to the system throughout the process. Microbubbles are treated as spheres in this model, whereby:


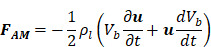

(14)

where


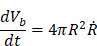

(15)

where over dots represent time derivatives.

Therefore, incorporating equations (5, 6, 8, 11 and 15) allow for the calculation of the bubble's translational acceleration:


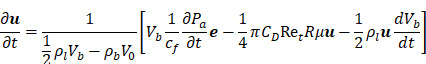

(16)

Equation 16 constitutes one half of a wave equation of translational motion, whose counterpart is a radial equation of motion described below 93:


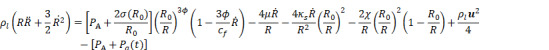

(17)

where 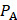 is the atmospheric pressure, 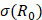 is the initial surface tension of the bubble shell (set to zero to comply with bubble stability), 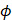 is the polytropic component of the gas core, 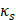 is the dilatational surface viscosity, and 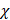 is the surface elasticity of the bubble.

In this case, the full derivation of equation (17) and an outline of its physical implications is available elsewhere 93. Relevant to the translational motion of microbubbles under the influence of ultrasound, equations (16) and (17) may be recursively solved using a variable-step, fourth-order Runge-Kutta method, providing the translational velocity 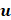 and thereby the displacement 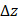 within a single sonication pulse. This is particularly useful when measuring bubble displacement directly using Particle Image Velocimetry 93:


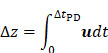

(18)

where 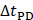 stands for the pulse duration and 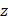 is the bubble translation.

Therefore, both the expected force applied by an acoustic wave and the velocity of a population of microbubbles within that wave may be calculated. This may be used to model the passage of drug through mediums of differing viscosity.

The streaming effects seen in the intravitreal ELIP delivery study by Thakur *et al*. (2019) showed significant bubble travel through the vitreous, particularly when applying repeated 30 second sonication treatments to the tissue 42. This is particularly encouraging, as it demonstrates the possibility of targeted drug delivery even through a viscous medium, such as the vitreous.

### 3.6. Factors influencing efficiency of drug delivery

#### 3.6.1. Ultrasound transducer wave characteristics

Ultrasound may be delivered through a myriad of transducer types capable of influencing the characteristics of the acoustic wave. Transducers range from simple, planar delivery, to phased array devices where multiple elements are assembled and act in concert to alter the direction and target of the acoustic wave. A thorough review of ultrasound probes and their various makes and purposes has been written by De Luca *et al.* (2021) 95. Additionally, a systematic review of ultrasound-mediated drug delivery through the blood-brain barrier by Gandhi *et al.* (2022) includes additional experimental transducers beyond what has been investigated with ocular drug delivery applications 11. The ultrasound transducers used in ocular drug delivery to date have included single-element planar and focused transducers.

##### 3.6.1.1. Single-element planar transducers

The majority of included studies used unfocused cylindrical planar piezoelectric transducers to facilitate ocular drug delivery. Ultrasound sources, such as the piezoelectric crystal in the transducer head, may be considered as a collection of point sources radiating individual acoustic wavelets into the tissue 96. If the transducer diameter is considerably greater than the ultrasound wavelength, there will be many regions of constructive and destructive interference established in the tissue. This effect will be greatest in the zone nearest to the acoustic source and will become progressively less dramatic as the wave travels away from the transducer tip 97. This area of variable sonication pressure is termed the Fresnel zone (aka the Near zone), and the distance from the transducer (for a disk shaped transducer) where this effect will dominate may be described by 96:


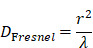

(19)

**Figure 7:**
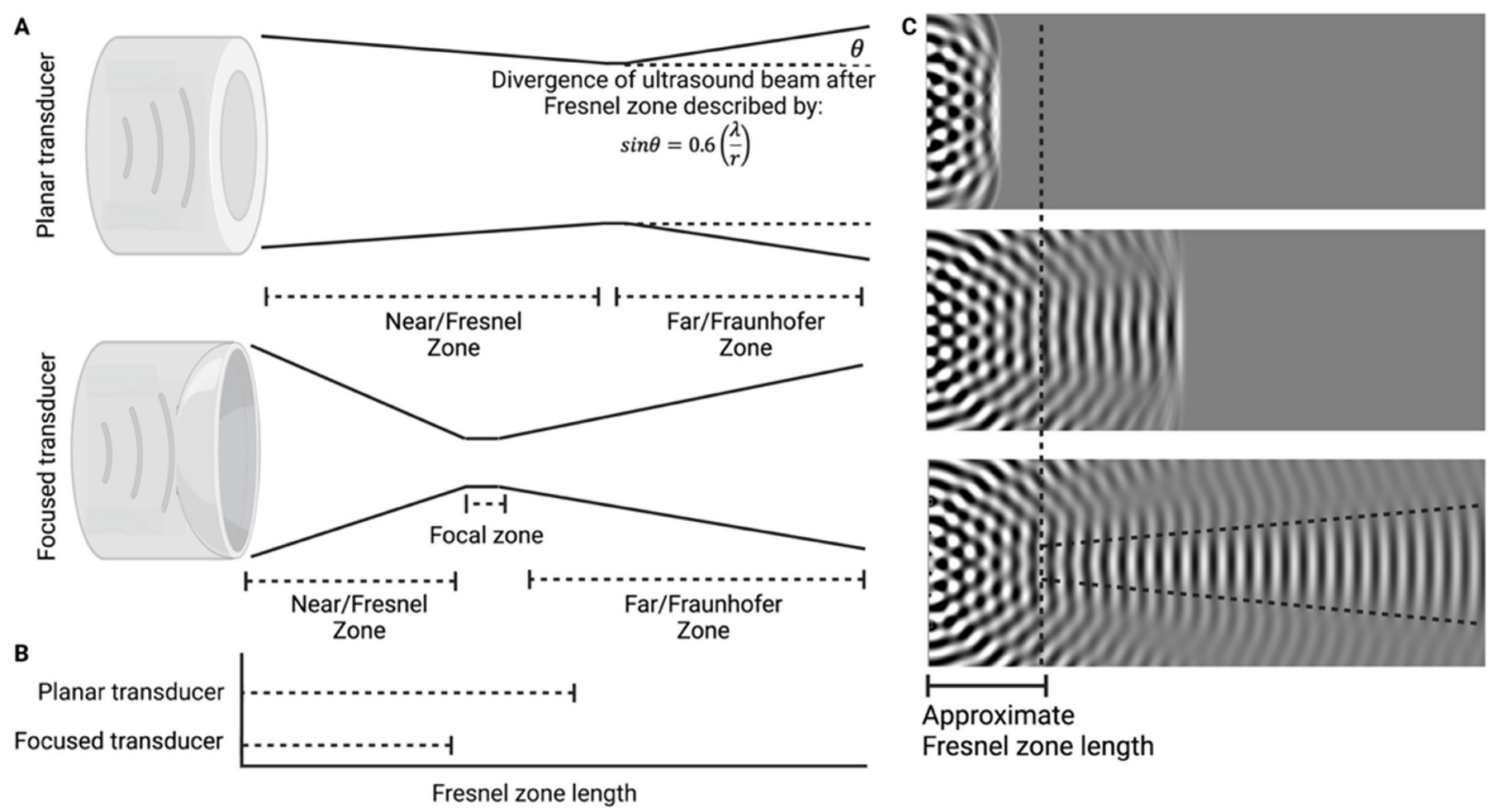
pictorial and graphical representation of ultrasound waves within the Fresnel and Fraunhofer zones. **A,** the ultrasound beam diverges beyond the Fresnel zone, commonly by 8-10°, **B,** Focused transducers functionally reduce the Fresnel zone length compared to planar transducers of the same diameter. **C,** Graphical representation of the interaction between ultrasound waves produced by a planar transducer, before and after the Fresnel length. Figure 7A, B, created using BioRender.com with graphics, 7C, produced at www.falstad.com/ripple/.

Thus, the Fresnel zone length, 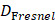, is dependent on both the ultrasound beam radius and wavelength. Within the Fresnel zone, the output pressure of the transducer within cross-sectional planes is unpredictable. Whilst it is possible to model the expected pressure of a point within this zone 98, it is far more practical, and reliable, to empirically measure the pressure output at the applied location using a hydrophone. The Fraunhofer zone (aka the far zone) is described by the region of ultrasound beyond the Fresnel zone. In this region the ultrasound pressure stabilizes, a result of the superposition of all ultrasound wavelets from the transducer surface, as per the 'Huygens-Fresnel' principle, thus the wavefront appears planar (Figure 7) 98. In addition, beyond the Fresnel zone, some of the acoustic energy will disperse along the periphery of the beam to create an increasingly divergent, and therefore less concentrated ultrasound wave described by 96:


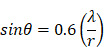

(20)

where 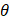 is the Fraunhofer divergence angle in degrees (Figure 7).

Authors have incorporated various methods to standardise the magnitude of ultrasound delivered to tissue. 2 studies opted to conduct their experiments at the intersection between the Fresnel zone and the Fraunhofer zone, the Focal zone, where the pressure output of the transducer is predictable 16, 70. Alternatively, 2 other studies opted to measure the pressure output at a set distance within the Fresnel zone and thereafter conducted all experiments at that distance 43, 62. Whilst both methods can deliver reproducible results, it is important to consider the practicalities associated with working in the Focal zone; one study using this method positioned their 15 mm diameter transducers 1.5, 2.25, 3.0 and 3.75 cm (for frequencies of 0.4, 0.6, 0.8 and 1 MHz, respectively) away from the scleral surface to maintain the tissue within the Focal zone. In practice, reliably maintaining coupling media over these distances would be difficult, and any increase in the transducer diameter would disproportionately increase this length. Therefore, working within the Fresnel zone may have a lower barrier to entry for clinical translation for larger area transducers. Alternatively, these issues may be overcome by altering the beam profile.

##### 3.6.1.2. Focused transducers

Focused transducers effectively draw the focal zone inward from the calculated distance of a planar transducer of the same diameter (Figure 7B). Within the focal zone of the focused transducer, the acoustic intensity may increase by factors greater than 100 compared to outside of the focal zone 96. This has the benefit of minimising the pressure experienced by non-target tissues, and, when used correctly, may significantly improve the safety of using ultrasound where the beam passes through delicate tissues such as the lens or cornea 99, 100. Given the friable nature of ocular tissue, and the potential damage that may be caused by mispositioning, understanding the focal length and focal area of a concave transducer is important. The focal length is the distance of maximal acoustic intensity from the transducer surface, the focal area is the cross-sectional area of the focal zone. These acoustic field parameters for a range of commercial focused transducers have been characterised elsewhere 101.

In ocular drug delivery, focused transducers have been used for both blood-retinal barrier disruption using exogenous microbubble delivery 15, 66, and trans- or intra-scleral delivery 49, 61. Park *et al.* (2012) used sixty 10 ms bursts delivered at a range of pressures including 0.81, 0.88 and 1.1 MPa through the cornea and lens to promote BRB opening. Whilst all parameters showed delivery of contrast agent though the BRB, 1.1 MPa PNP demonstrated severe extravasation of erythrocytes 66. Comparatively, Touahri *et al.* (2020) used double the number of 10 ms pulses at ranging intensities of 0.36-0.84 MPa, with reliable enhancement occurring above 0.7 MPa, although in this study there was significant intra- and inter- rat treatment and outcome variability, and safety outcomes were not linked to specific rats 15.

Focused ultrasound in trans-topical applications targeting the sclera have used far higher PNPs of 15.7 MPa (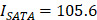 W/cm^2^) compared to trans-BRB studies. When testing a range of pulse durations (PD) between 10 and 100 ms, increasing PD increased both fluorescence intensity, penetration depth and area of delivery in *ex vivo* porcine eyes, but any duration above 50 ms incurred erosion of the scleral surface 49. Comparatively, a far lower intensity of 0.05 W/cm^2^ has been used continuously for 30 seconds to improve transscleral delivery of FITC-BSA without causing damage 61.

#### 3.6.2. Ultrasound parameters

**Figure 8:**
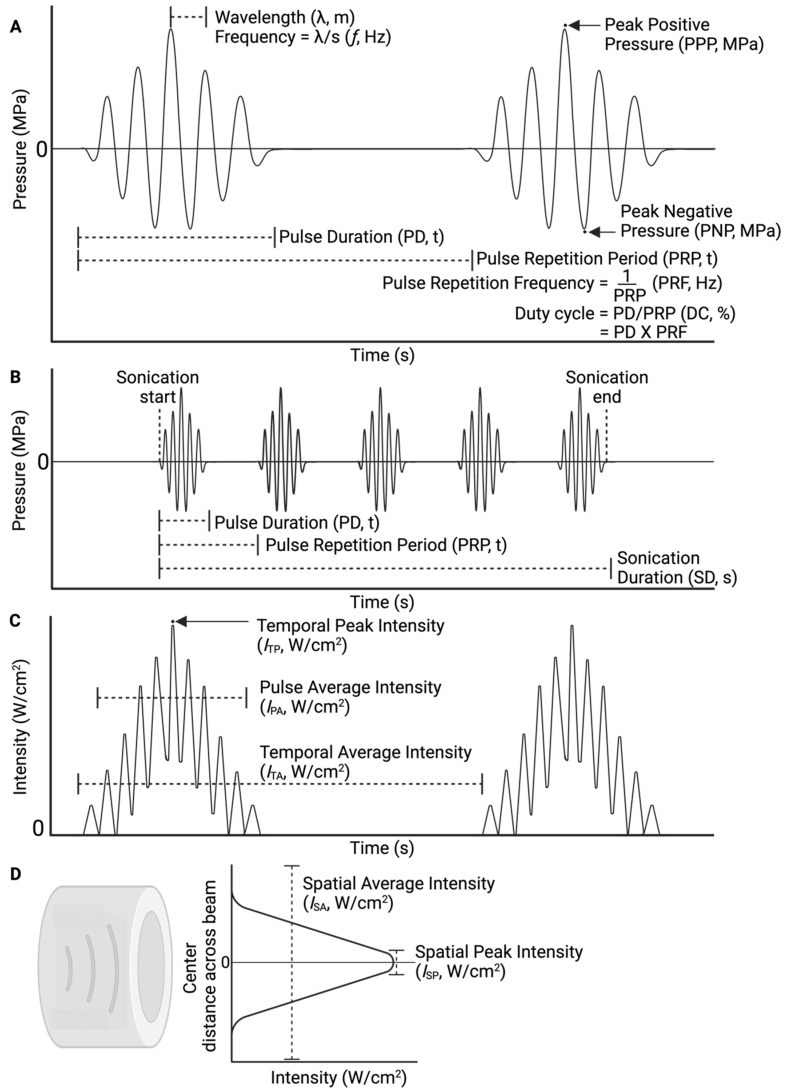
Overview of ultrasound wave properties and parameters. Ultrasound pressure and intensity may be expressed in terms of its spatial and temporal characteristics, which may be combined to describe various characteristics of the acoustic field. **A, B,** Ultrasound wave characteristics expressed as a function of the waveform pressure over time allows for the derivation of wavelength, PNP, PD, PRF and DC. **C,** Ultrasound wave characterised as a function of its intensity over time allows for the derivation of *I*_TP_, *I*_PA_, *I*_TA_. Measuring the intensity in different areas, **D**, allows for the assessment of *I*_SA_ and *I*_SP_. Temporal and spatial measurements may be combined to express ultrasound intensity as *I*_SPTP,_ *I*_SPTA_, *I*_SPPA_, *I*_SATP_, *I*_SATA_ and *I*_SAPA_. Created using BioRender.com

There are six parameters commonly altered to change the effect of the ultrasound wave on incident tissue, and therefore to influence drug delivery: frequency, power or PNP, duty cycle (DC), pulse repetition frequency (PRF), sonication duration (SD) and PD (Figure 8). When considering the commonly used ranges of ultrasound parameters, each a continuous variable and each having large impacts of the efficacy and safety of treatment, it becomes clear that considering any given parameter in isolation is erroneous. If each parameter had only 100 discrete values to change, this would result in 10^12^ combinations; the likelihood of choosing the most ideal set of parameters, or the ideal parameters to start with would be proportionally small. As such, each parameter has been characterised according to the common range of values used in experimentation. The effect of increasing or decreasing the parameter, where experimentally tested, has been described, in the context of the method of drug delivery chosen by the researchers, in Table 4. This table, in combination with a functional understanding of how each ultrasound parameter influences the impact to tissue and drug delivery, should be used as a guide toward choosing an appropriate set of starting parameters. In addition, these parameters are likely relevant only to the animal model tested; applying similar parameters that were effective in a mouse model will not guarantee efficacy when using the larger eyes of the cow. When considering clinical translation, the US-FDA requires acoustic output testing, and has outlined the minimum required parameters to be included in the testing methodology of transducers in detail 63.

##### 3.6.2.1. Frequency

Ultrasound frequency is the number of cycles of the acoustic wave per second, measured in Hz. In ocular medicine, ultrasound frequency is modulated depending on the application; Low frequency (22-50 kHz) ultrasound is used in phacoemulsion to physically degrade lens tissue through a 'jackhammer' effect to enable efficient removal and replacement of the lens in cataract surgery 102, 103. Comparatively, higher frequency ultrasound (7-20 MHz) is used in whole globe imaging applications 104, and extremely high frequency (35-100 MHz) has been used in ultrasound biomicroscopy to image the anterior segment with high detail 105. The parameters used in ocular imaging are often inappropriate for ultrasound-mediated drug delivery applications. Low (22-50 kHz) ultrasound frequencies with longer wavelengths increase the time for significant bubble growth and cavitation to occur, increasing not only the efficient translation of acoustic to mechanical energy into tissue, but the degree of tissue damage, too 103. Comparatively, at the higher frequencies used in ophthalmic imaging (7-100 MHz) both the penetration distance and cavitating potential of the ultrasound wave are drastically reduced 104, 105, which limits their application in drug delivery.

When considering frequency in drug delivery, the range used depends on the application: MBP_EXO_ applications use higher minimum frequencies compared to AS and MBP_ENDO_ methods (0.3MHz compared to 0.02MHz) and similar maximum frequencies (3 MHz, Table 4). One reason for the higher minimum frequency used in MBP_EXO_ relates to the presence of the exogenously administered microbubbles, which display a resonant oscillatory frequency inversely proportional to both the bubble size and sonication pressure 106, 107. The commercial microbubbles used for MBP_EXO_ in the reviewed studies ranged between 1 and 5 µm in mean diameter 15, 17, 36, 46, which, at a sonication pressure of 0.1MPa, results in a range of resonance frequencies between 0.38 MHz and 2.98 MHz 108. Whilst no study using MBP_EXO_ compared the effect of changing frequency on drug delivery, each study is subject to survivorship bias; the frequency range that works is the frequency range that is published, and this range is between 0.3-3 MHz for MBP_EXO_ applications.

Comparatively, AS and MBP_ENDO_ studies rely on lower frequencies to encourage the development of cavitation nuclei from dissolved gasses in the target media. In addition, these applications are more commonly applied to intra- and trans-scleral drug delivery, which relies on some measure of tissue damage to improve the efficiency of drug delivery (Table 4). Chau *et al.* (2017) demonstrated a clear relationship between particle penetration of the rabbit sclera whereby penetration depth was inversely proportional to both particle size and applied frequency. After 30 seconds of sonication, 20 kDa dextran penetrated the entirety of the rabbit sclera at 40 kHz, but only 46% of the scleral depth at 500 kHz, whilst 70 kDa dextran penetrated 33% and 12% of the sclera at 40 and 500 kHz, respectively. The 150 kDa dextran did not appreciably penetrate the sclera at any parameter, and at higher frequencies of 1 and 3 MHz, only 20 kDa dextran penetrated the sclera 72. Lower frequencies increase the magnitude of cavitation of dissolved gas bubbles whilst higher frequencies increase the velocity of acoustic streaming in a solution, improving the speed of drug delivery 90. Investigations comparing the effect of sonication frequency on transcorneal or transscleral drug delivery showed as frequency decreased, drug delivery increased 16, 61, 70, 72, 74. Unfortunately, only 2 of these studies included measurements of PNP, which allows for the calculation of mechanical index 61, 74. What becomes clear after considering the combination of frequency and PNP, is that penetration of drug through the sclera correlates to an increasing mechanical index (MI), which also correlates to an increase in the histological damage seen on the eye surface. MI characteristics are further outlined in section 3.6.3.3

##### 3.6.2.2. Power

Power output of an ultrasound transducer describes the magnitude of energy delivered by the ultrasound beam. The measurement of power output of an ultrasound transducer is dependent on both spatial and temporal factors; spatial measurements may consider only the area of peak intensity (spatial peak, *I*_SP_), or the total transducer area transmitting the wave (spatial average, *I*_SA_), whilst the temporal factors may consider a measurement averaged over only the pulse duration (pulse average, *I*_PA_), the period encompassing the peak intensity (temporal peak, *I*_TP_) or the average intensity spread across the entire sonication duration (time average, *I*_TA_) 109. These spatial and temporal measurements may then be combined in 6 configurations, as listed in Figure 8.

Safety assessments prescribed by the TGA for ophthalmic ultrasound outline a maximum *I*_SPTA_, derated by 0.3 dB MHz^-1^ cm^-1^, *I*_SPTA.3_, of 0.05 W/cm^2^. This value may be obtained by measuring the maximum ultrasound intensity emitted by the transducer, *I*_SP_, in water using a hydrophone, averaged over the Pulse Repetition Period, *I*_TA_, derated by 0.3 dB MHz^-1^ cm^-1^. Deration of the ultrasound output is applied due to assumed losses in ultrasound intensity as the pressure moves through tissue. The I_SPTA.3_ may be calculated by multiplying the derated Pulse Intensity Integral, 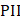, by the pulse repetition frequency (PRF) 110:


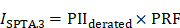

(21)

where 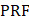 is the frequency by which ultrasound pulses are delivered per second, and the peak derated Pulse Intensity Integral (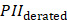) is 110:


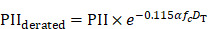

(22)

and where the factor 0.115 is the conversion between decibels to neper, (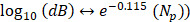, 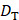 is the distance from the transducer depth of interest, and 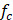 is the transducer centre frequency. 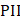 may be calculated from output voltages of a hydrophone measuring the PNP of the acoustic beam by using 110:


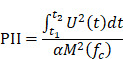

(23)

where 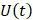 is the hydrophone voltage, 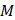 is the hydrophone sensitivity at the centre frequency, 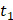 and 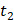 represent the time duration of interest.

Any power output above an *I*_SPTA.3_ of 0.05 W/cm^2^ in the included studies, with the intention of clinical translation would require specific approval by the US-FDA before being delivered to market, a potential barrier to entry for clinical translation. This is the case for devices capable of displaying the approximate delivered mechanical and thermal indices during use. For translation of transducers incapable of displaying these values, the US-FDA severely limits power output to an *I*_SPTA.3_ of 0.017 W/cm^2^ 63.

Unfortunately, the vast majority of included studies only measured the *I*_SATA_, as it is the best predictor of tissue heating, which is a separate US-FDA regulated safety limit discussed elsewhere in this review 111. *I*_SATA_ is commonly measured using one of three ways: the calorimetric method, using a hydrophone, and using a radiation force balance (RFB). Historically, the RFB was primarily used to measure ultrasound power output, whereby the radiative force of the ultrasound directed into a highly absorbing target is measured by a microbalance 70. This method is time intensive, costly, and is incapable of measuring large power outputs due to the risk of damaging the microbalance 112. As such, an alternate method relying on heat flux mechanisms has been developed, termed the calorimetric method, whereby the transducer is positioned in an insulated vessel of known liquid and volume, and the temperature rise is measured over a given period. The ultrasound power may be derived from the following equation 39, 61, 69, 72:


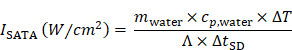

(24)

where *I*_SATA_ is the time average spatial average intensity of the ultrasound transducer (W/cm2), 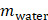 is the mass of water (g), 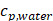 is the specific heat capacity of water (J/g·K), ΔT is the change in temperature (K), 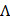 is the cross-sectional area of the transducer probe (cm2) and Δ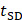 is the time for the sonication duration (s).

Finally, ultrasound power may also be measured using a hydrophone, whereby the hydrophone is initially positioned at the area of highest pressure from the transducer (*I*_SP_), and the plane parallel to the spatial peak position is measured in a 1 cm x 1 cm grid using the “pressure squared interval mode.” The data obtained is filtered by omitting values ≤ 0.25 of the peak intensity 113, and the intensity can be calculated by the equation 43, 73:


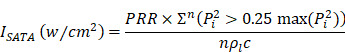

(25)

where 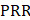 is the pulse repetition rate (equal to the frequency of ultrasound, 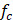, for a continuous wave) (Hz), 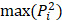 is the maximum pressure-square integral, 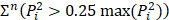 is the sum of all data within the matrix measured by the hydrophone with a value > 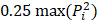, 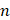 is the number of data points > 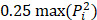 multiplied by the size of the grid (cm^2^), or the effective radiation area (cm^2^).

The effect of altering ultrasound power output on drug delivery has been extensively investigated in both MBP_EXO_ 33, 36 and AS or MBP_ENDO_ applications 43, 45, 60, 62, 64, 70, 73. When considering MBP_EXO_ applications: increasing the sonication power did not significantly increase the trans-BRB delivery and expression of delivered plasmids in rats, but one study found a positive trend with increasing power that was statistically insignificant 36. In another study an increase in power was associated with a significant decrease in plasmid expression density 33. Importantly, when comparing these 2 studies, the latter found that increasing power significantly increased the rate and severity of side effects, to the extent that the expression in groups of mice exposed to higher powers could not be determined due to the excessive intraocular haemorrhage 33. This may explain the inverse relationship between ultrasound power and transfection efficiency, as the toxicity of the higher power ultrasound exposure may have impeded transfection. Comparatively, the former study did not show a correlation of power and histological or gross signs of damage. This should not imply increasing power does not increase tissue damage, but rather at these sonication parameters, microbubble dose and rat model, this range of ultrasound powers appears safe 36. When considering AS and MBP_ENDO_ applications, safety assessments of porcine scleral and corneal tissue showed signs of topical damage at higher power outputs 43, whilst other researchers working with rabbits only reported greater damage when comparing any sonicated group to non-sonicated control tissue 60, 62, 70, and another did not assess epithelial tissue changes 73. Importantly, all of the studies showing epithelial damage assessed the tissue within 10 70, 20 60, 62, or 30 43 minutes of sonication. When tissue was assessed 90 minutes post sonication, epithelial damage had mostly resolved 60, 62, and by 6 hours post sonication, epithelial damage had completely resolved 70. This highlights the fast regeneration capacity of ocular epithelium, but also shows the importance of completing immediate and delayed safety assessments post sonication. Efficacy of drug delivery appears to display a parabolic relationship with ultrasound power 43, 64, 70, 73. Whereby ocular permeability increased from low to medium ultrasound power outputs, but then decreased from medium to high power exposure. The reason for this is likely related to cavitation activity, for which PNP directly contributes.

##### 3.6.2.3. Peak negative pressure

PNP represents the magnitude of rarefactional force output by the ultrasound transducer compared to ambient pressure. The mechanical impact this force has on tissue is also dependent on the frequency of the ultrasound wave. As such, both the frequency and PNP are required to describe mechanical index (MI), an approximate, unitless measure of the non-thermal effects of ultrasound 114:


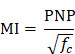

(26)

where 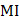 is the mechanical index (arbitrary units) and 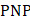 is the measured peak negative pressure (MPa).

Safety assessments of ultrasound involving mechanical index are discussed in detail at a later point in this review. MI is important when considering the likelihood of cavitation occurring in a medium, which is not possible to determine using power measurements alone. Whilst PNP and power are both measures of the magnitude of ultrasound impact applied to tissue, and increasing the power will also increase the PNP, the derivation of one to another is not possible and they need to be determined separately. Mechanical index, whilst useful for characterising the likelihood of cavitation, is limited as it does not consider the cumulative effect of sonication over time, which is impacted by the SD and pulse characteristics 108. Importantly, PNP should be assessed in water at the same distance from the transducer as the target tissue, using a hydrophone. The measured output voltage is then converted to pressure by dividing the hydrophone voltage by an appropriate sensitivity constant relative to the working frequency specific to the hydrophone 115. Historically, it would be common to then derate the measured pressure by 0.3 dB cm^-1^ MHz^-1^ which represents a conservative average estimate of the overall attenuation of ultrasound power occurring in soft tissue. In contrast to standard practice, it is more appropriate to consider the attenuation coefficient of each tissue barrier absorbing the ultrasound beam, which are known for ocular tissues, and have been included in Table 5. This more accurately reflects the actual magnitude of delivered ultrasound into ocular tissues and is further discussed in section 3.8.3.

Whilst the effect of changing PNP has been investigated in all three methods of drug delivery 43, 60, 62, 66, only one study considered the attenuation of the ultrasound wave across the ocular barrier, by incorporating the attenuation coefficient and thickness of the lens through which the ultrasound was being applied 66. This was the only study to assess the effect of altering PNP on MBP_EXO_-mediated drug delivery, and the researchers found that increasing the PNP significantly increased the signal intensity of the peripherally injected gadolinium contrast media in the sonicated areas of the retina, when viewed by MRI. However, as expected the higher pressure also significantly increased the development of deleterious petechiae within the retina of treated rats 66. Comparatively, three studies utilising AS and MBP_ENDO_ measured both power output and PNP in both porcine and rabbit eyes 43, or rabbit eyes only 60, 62. In these studies, the penetration of drug appeared parabolic in relation to the acoustic pressure administered. This becomes clear when considering the cavitation activity at different pressures; at the pressures most effective for drug delivery the researchers recorded subharmonic frequencies characteristic of stable cavitation. Comparatively, at higher intensities displaying reduced drug penetration the PCD recorded the presence of broadband frequencies indicative of inertial cavitation. Thus, for transscleral or transcorneal drug delivery, optimal improvements in permeability occur when primarily inducing stable cavitation within the coupling media 43, 60, 62.

##### 3.6.2.4. Duty cycle, pulse repetition frequency, pulse duration, and sonication duration

Ultrasound can be delivered either continuously or in individual packets interspersed between periods of inactivity. Both methods are employed for ocular drug delivery. Of the studies included in this review, 74% of the studies delivering drug via MBP_EXO_ used pulsed application, comparatively, 82% of studies utilising AS or MBP_ENDO_ used continuous sonication. The three parameters that impact the pulse characteristics of the ultrasound wave are DC, PD and PRF, which can be described by 108:


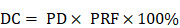

(27)

or


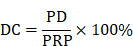

(28)

where 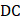 (%) is the proportion of ultrasound “on” time to “total” time, 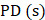 is the length of the ultrasound pulse, and 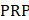 is the pulse repetition period, which represents the reciprocal of 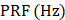.

Delivering ultrasound in pulses not only reduces the total acoustic energy delivered to the tissues, but also allows for thermal dissipation to take place between pulses, resulting in a lower final temperature in the sonicated tissue compared to continuous sonication. This is particularly relevant for highly vascularised tissue, such as the liver or kidney, where blood flow quickly transports heat away from the sonicated area 116. Interestingly, more studies involved in transscleral or transcorneal drug delivery used continuous ultrasound application in a tissue where the minimal vasculature reduced any effect of thermal dissipation due to blood flow. These parameters also impact the duration of time the various mechanisms influencing ultrasound-mediated drug delivery will have to do work; acoustic streaming and the creation- and/or cavitation- of dissolved or exogenously administered microbubbles occur only when acted upon by the ultrasound wave. Particularly relevant for BRB drug delivery using intravenously administered microbubbles, pulsing ultrasound allows time for the microbubble population to replenish within the targeted vasculature 117. Indeed, 2 studies using the same mechanical index will not have sonicated tissue to the same extent if they are using different pulse parameters. As such, perhaps an additional method of assessing the mechanical aspects of ultrasound on tissue should be implemented to incorporate cumulative ultrasound dose:


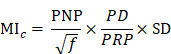

(29)

where 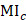 is the cumulative mechanical index and 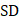 is the total sonication duration.

This highlights the importance of comprehensive reporting of ultrasound parameters, to ensure replicability of published results. Of the included studies, one failed to include the applied ultrasound frequency 77, and a further six studies did not include information regarding the PD or PRF of applied ultrasound, making reliable replication of their experimentation impossible 36, 37, 64, 65, 75, 76.

When considering ultrasound-mediated drug delivery, no studies assessed the effect of changing PRF directly, however studies did assess the effect of changing the DC and PD 49, 64.One included study investigating vitreal/vitreoretinal delivery demonstrated that by reducing the DC from 100% to 50%, the increase in small (53 nm) nanoparticles across the *ex vivo* bovine neural retina into retinal pigment epithelial cells was doubled, however this trend was not maintained for larger (131 nm) nanoparticles 64. It should be noted that the PRF was not stated, and as such the pulse duration is unknown. Also, the ultrasound delivered was not measured in PNP, nor was a passive cavitation detector employed to investigate cavitation activity; thus, determining the cause of the improved nanoparticle delivery is difficult. Despite this, a hypothesis around the effect of pulsed ultrasound may be proposed. As nanoparticle was delivered into the tissue via MBP_ENDO_ and/or acoustic streaming, the pause between pulses may allow additional nanoparticles to diffuse into the beam path, whereas otherwise the constant pressure may inhibit particle movement into the area. This has been suggested as a potential mechanism of improved flavonoid extraction from plant tissue in industry practice 118.

Only one study, using a HIFU ultrasound device, has examined the effect of increasing pulse duration on transscleral ocular drug delivery in *ex vivo* porcine eyes. The researchers maintained the PRF at 1 Hz and tested pulse durations of 10, 20, 50 and 100 ms, which correspond to a duty cycle of 1, 2, 5 and 10%. Increasing the pulse duration significantly increased the transscleral delivery of bicinchronic acid, to the extent that 100 pulses at 100 ms/pulse allowed the bicinchronic acid to penetrate the entire scleral distance 49. Unfortunately, SEM and fluorescence microscopy showed severe erosion of the scleral surface above a 50 ms pulse duration, indicating the higher cumulative ultrasound dose may cause tissue damage. In addition, the study did not standardise the cumulative dose of ultrasound applied, i.e, the improvement in bicinchronic acid delivery may have been dependent on the total sonication dose. This may have been tested by also delivering 200 pulses of 50 ms ultrasound, 500 pulses of 20 ms, and 1000 pulses of 10 ms ultrasound into tissue.

Sonication duration determines the total time ultrasound is applied, and has been definitively studied in AS- and MBP_ENDO_-mediated drug delivery 42, 43, 64, 69. Studies using exogenous microbubbles primarily used shorter (60-300 s) sonication durations compared to those relying on dissolved gas or AS (300-600 s). The assorted studies clearly show a positive trend correlating improved drug delivery with longer sonication durations (Table 4). Only one study assessed safety parameters at different sonication durations, whereby increasing the sonication duration from 60 to 240 s caused clouding of the lens, tissue dehydration and retinal delamination in *ex vivo* bovine eyes 42. The total duration of applied ultrasound can be considered as a multiplier of each of the other ultrasound parameters, increasing cumulative mechanical and thermal effects, and thereby increasing both the efficacy of drug delivery and risk of adverse events.

#### 3.6.3. Microbubbles

The typical use of microbubbles in ultrasound-mediated drug delivery has been extensively characterised elsewhere 119, 120. As such, only microbubbles used in included studies and factors relevant to ocular drug delivery will be discussed herein. Exogenous microbubbles are primarily included in ultrasound-meditated drug delivery as cavitation foci, which reduce the required sonication intensity required to initiate cavitation activity 121. Microbubbles have been used in both MBP_EXO_ and AS applications for ultrasound-mediated drug delivery (Table 6). Importantly, the type of microbubble used, their concentration, physicochemical properties, and size all contribute to their interaction between both acoustic field and surrounding environment. Relying on commercial microbubbles traditionally used for acoustic contrast imaging is common, as their safety in human use has been extensively tested. Some studies have opted to synthesize their own microbubbles, which holds the advantage of allowing researchers to design the microbubble to their chosen task. An understanding of what factors influence bubble behaviour, their effect on cavitation activity or drug delivery, and how to modify these factors, would be of benefit when attempting to optimise drug delivery.

##### 3.6.3.1. Concentration and size

Microbubbles used in medical imaging applications must be safe to inject into the bloodstream, and thus are size limited to < 10 µm, due to their need to traverse microvasculature without occluding flow 120. Increasing the microbubble diameter will disproportionately increase the volume of entrapped gas, as per the square-cube law, and the largest contributing factor to magnitude of cavitation is the amount of gas delivered to the ultrasound beam. When matching microbubble concentration, larger microbubbles elicit more intense cavitation profiles compared to smaller microbubbles 122. In addition, higher concentrations of monodisperse microbubble populations will both elicit greater cavitation compared to less concentrated solutions 123, and will begin cavitating (both stable and inertial) at lower sonication pressures 124. Finally, when combining the microbubble size and concentration into a measure of the gas delivered, matching the microbubble volume dose (MVD) results in similar onset and peak of cavitation activity irrespective of microbubble size or concentration 122. Thus, a useful way of comparing the amount of microbubble delivered in alternate studies may be to compare the MVD, rather than bubble size or concentration alone. An additional factor to consider is the impact microbubble size has on their resonant frequency (the frequency of maximal cavitation), an aspect beyond the scope of this review, and whose methods for calculation have been characterised elsewhere 108. Once the preferred size of microbubble is chosen and the ultrasound parameters are optimised, the magnitude of cavitation activity may be up- or down-titrated by altering the delivered microbubble dose.

As described above, microbubble concentration is closely correlated with the magnitude of cavitation behaviour, and thus the efficacy of drug delivery and/or risk of adverse effects. Park *et al.* (2012) studied the effect of ultrasound and microbubbles on BRB-mediated delivery of an MRI contrast agent of rats *in vivo* 66. The researchers targeted 5 areas of the retina, spaced 2 minutes apart, with a bolus dose of Definity® microbubbles delivered via tail vein to act as cavitation foci directly before each sonication. The authors cited using 2 minutes between boluses to allow for the majority of the previously injected microbubbles to be eliminated from the circulation. However, given that the circulation half-life of Definity® microbubbles in rodents is 6.8±4.88 minutes 125, and the degree of cavitation appears linearly proportional to the concentration of contrast agent in the blood 123, each subsequent bolus will have resulted in greater BRB-opening and/or damage due to ever-increasing intravascular microbubble concentrations. This highlights the utility of coincident use of a PCD during sonication, to help identify the extent of cavitation activity during experimentation, which may have allowed this issue to have been recognised.

##### 3.6.3.2. Choice of echogenic agent

Microbubbles must contain an echogenic agent, typically a gas, which acts as both a reflecting surface in imaging, and a cavitation focus during cavitation-assisted drug delivery. To remain stable during administration and to extend *in vivo* residence, the gas needs to linger within the enclosing shell. Increasing gas molecular weight and decreasing aqueous solubility aids these factors, by reducing the rate of gas diffusion into the surrounding aqueous media of the blood 120. This has led to the use of fluorinated gases, e.g., C_X_F_Y_ or SF_Y_, which are suitably large, hydrophobic, and inert, and thus act as ideal gas core agents. Upon dissolution into the blood, dissolved gases are delivered to the lungs, and exhaled efficiently during gas exchange 126. During bubble rupture under sonication forces, where larger amounts of gas are released into the surrounding vasculature, the unencapsulated gas quickly dissolves within milliseconds, minimizing the risk of air embolism 127.

##### 3.6.3.3. Choice of shell composition

Microbubbles must be encapsulated within a shell to minimise the thrombogenic risk associated with their propensity for rapid plasma protein adsorption, and to decrease the risk of air-embolism induced by bolus intravenous delivery of free gas 120. In addition, the encapsulating shell will stabilise the contrast agent against dissolution or coalescence, and will affect how the microbubble responds to applied forces 128. shell composition may include lipids, surfactants, polymers, proteins, or a combination of these constituents. Permutations to these core ingredients, for instance the addition of polyethylene glycol (PEG) polymers or targeting ligands, can be used to alter how the microbubbles behave *in vivo* 129. Relevant to cavitation-induced drug delivery, the parameters being altered by changing the shell components include the shell thickness, viscosity, stiffness, friction and surface tension 130. The interactions between these properties and ultrasound waves are used to estimate how a microbubble will oscillate within an acoustic pressure field and have been extensively reviewed elsewhere 130-132. How these parameters impact microbubble oscillation are beyond the scope of this review, however the shell stiffness and friction of common commercial microbubbles have been included in Table 6 for convenience.

##### 3.6.3.4. Commercial microbubbles

Commercial microbubbles include those developed for- and used in- practice as acoustic contrast agents. Of these, SonoVue®, Definity®, Optison™ and Artison® microbubbles have been used in ocular drug delivery studies (Table 6). SonoVue® and Definity® microbubbles rely on a phospholipid shell containing either SF_6_ or C_3_F_8_, respectively, with a primary difference in shell composition being the PEGylated phospholipids present on Definity® microbubbles. The presence of these large PEG chains protect microbubbles from their surroundings through steric hindrance, effectively preventing coalescence and macromolecule adsorption, improving circulation, and minimising immunogenicity 133. Comparatively, Optison™ microbubbles use an albumin shell, aimed at reducing the immunogenicity of their microbubbles by utilising a protein commonly produced in the body (Table 6). The commercial microbubbles used in the included studies range from 1.1 to 4.5 µm in diameter, and the microbubbles produced by SonoVue®, Optison™ and Artison® have been used primarily for local injection directly into tissue during sonication. Definity® microbubbles have been used for BRB drug delivery, in small rodents using systemic injection. The delivered dose varies between studies, with a 10-fold difference in dose between the 2 studies using Definity® microbubbles. Both of the studies used a peripheral intravenous injection of microbubbles through the rat tail vein, using 20 µL/kg 66, and 200 µL/kg 15, respectively, followed by focused ultrasound application to regions of the retina. Comparatively, when injecting microbubbles directly through intravitreal injection in small rodents, researchers primarily used smaller, 1-5 µL doses of microbubbles with one outlier of 40 µL delivered into rat subconjunctiva (Table 6) 37. Rabbit studies involving intravitreal injection relied on larger, 100 µL doses of microbubbles 46, which were reduced to 2 µL when injected into the cornea 36.

##### 3.6.3.5. In-house microbubbles

Researchers have also engineered their own microbubbles to aid drug delivery. These studies primarily described their microbubbles in terms of mean size, shell composition, and concentration, with only one study by Al Sadiq *et al*. (2021) assessing shell rigidity or stiffness parameters in a referred study 42, 134. Interestingly, one study referred to a prior published review for their microbubble production method 75, however the referred review does not describe any production techniques 135. Only one study, by Yamashita *et al*. (2007), has investigated the difference in transfection efficiency between Optison™ microbubbles and their in-house engineered echogenic liposomes (ELIPs) 37. These researchers found their ELIP formulation significantly increased gene expression of injected rat conjunctiva treated with ultrasound compared to Optison™ microbubbles (3.6 vs 2.0, respectively, mean fluorescence scores on a qualitative 5-point scale) 37. The ELIPs differed in their size (filtered up to 0.2 µm ELIPs vs 3-4.5 µm Optison™) and shell composition (phospholipid mix vs albumin shell) when compared to Optison™ bubbles. Given the significant improvement in transfection efficiency when using in-house microbubbles shown by Yamashita *et al*. (2007), further studies examining the mechanisms behind improved drug delivery efficiency between bubble compositions would be advantageous. The use of in-house microbubbles in the included studies typically demonstrate effective drug delivery, however the sporadic lack of information surrounding microbubble size, concentration, gas, and shell composition makes it difficult to determine the contributing factors which promote the demonstrated ideal vehicle characteristics. A set of accessible assessments for determining basic physicochemical microbubble characteristics of the in-house microbubbles would be beneficial in this area.

### 3.7. Applications in animal models

The choice of animal species used in ocular research should be balanced between accessibility factors and functional factors. Accessibility factors may include cost of purchasing and maintenance, the rigidity of animal ethics, the difficulty of handling and the models of disease available in the given species. Comparatively, functional factors relate to the applicability of the experimental results in humans, the sensitivity and specificity of the method of ultrasound-mediated drug delivery as it applies to characteristics of the eyes (e.g., bovine eyes allow more sensitive quantification of the contribution of acoustic streaming toward improved drug delivery compared to the smaller mouse eye), and the translatability of the animal disease model to the human. Unfortunately, animal species with excellent functional factors tend to also exhibit high accessibility barriers; for instance, the Rhesus and Cynomolgus monkeys present an ideal ocular model in human diseases due to their evolutionary similarities 136, but they are prohibitively expensive, difficult to handle, (rightfully) require fastidious ethical approvals and their availability is limited 137. It is therefore unsurprising that most studies relied on rabbits or rodents for their drug delivery assessments (Table 3).

#### 3.7.1. Small rodents

Mice and rats, used in 46 % of the included studies, represent an accessible, albeit functionally limited ocular model. Rodents are relatively cheap to purchase, handle and maintain, have a lower barrier to animal ethics approval, and there are multiple ocular disease models available 138. Despite this, the vast differences in retinal cell density, overall eye size, and thickness of the ocular structures limit the translation of results gathered in rodent studies toward human application 139. All included studies assessing rodents claimed some amount of progress toward clinical translation, however only one study justified their choice in animal as the rodents were “readily available and inexpensive” 64. In addition, only 2 of the studies acknowledged that the differences between rodent and human eyes may limit direct translation of results 45, 75. 16 of the 17 rodent studies investigated *in vivo* models, whilst the remainder used *ex vivo* mice. 15 of the 16 *in vivo* rodent studies used MBP_EXO_ to improve delivery via the BRB, vitreal/vitreoretinal and/or by direct injection into the target tissue. Of these studies, 14 delivered genetic material whilst the remaining study demonstrated BRB delivery of gadolinium contrast agent (Table 3). The remaining 2 studies utilised MBP_ENDO_, one assessed the efficacy of an intravitreal injection of PEDF loaded into immunoliposomes in an *in vivo* rat model of CNV 76, and the other assessed the trans-topical delivery of gatifloxacin into both *in vivo* and *ex vivo* mouse corneas 45. AS may have contributed to the effects of 5 of the 17 rodent studies but was not intentionally examined (Table 3).

**Figure 9:**
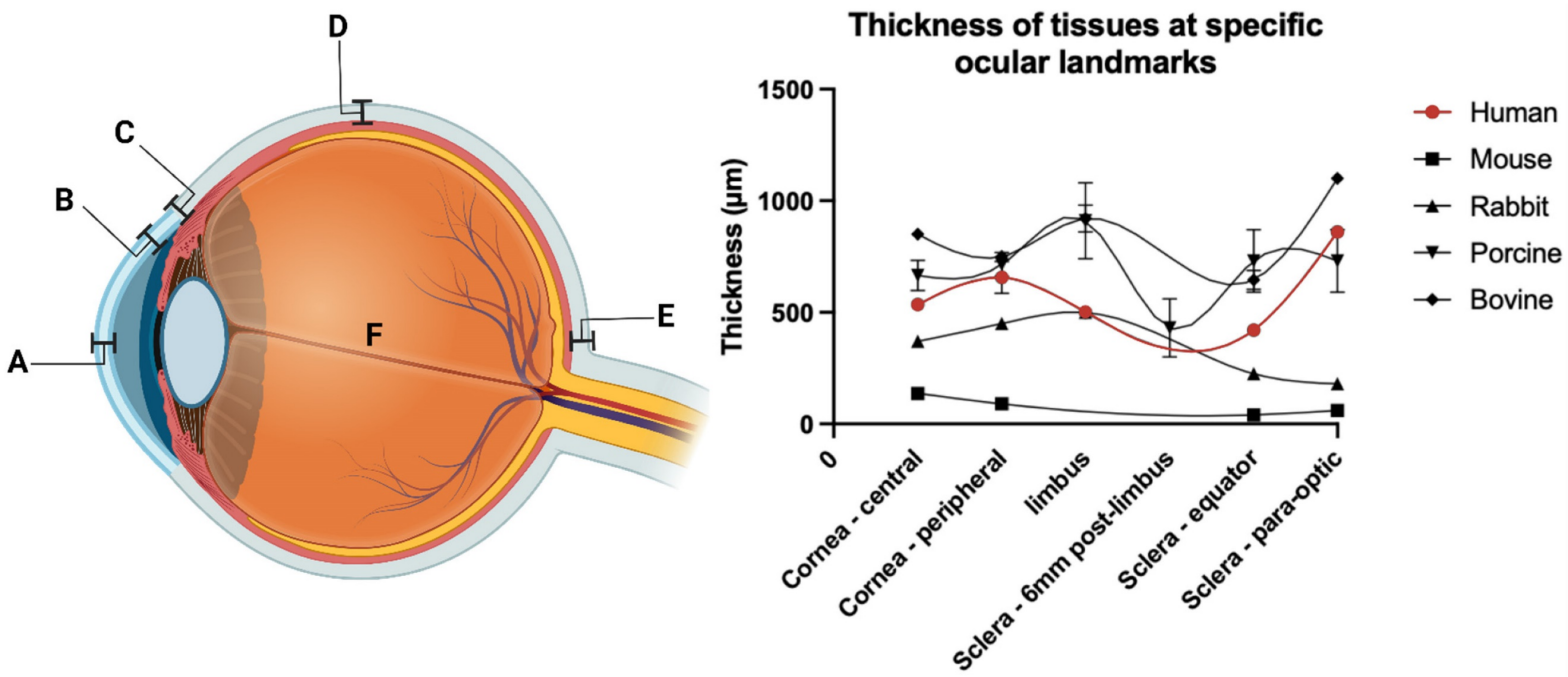
Corneal and scleral thickness differ between species and landmarks. Transcorneal and transscleral ultrasound-mediated drug delivery efficiency will depend on the thickness of the tissue through which drugs are delivered, therefore the choice of drug delivery location should be justified by linking the anatomical similarities of the chosen model with that of the human. Created using BioRender.com

The extensive use of gene delivery in rodent models highlights the advantage of using the small rodent eye for these purposes; all parts of the eye are accessible for injections, the small area helps minimise the work associated with imaging and quantification of transfected cells, and intravitreal injections into the small globe massively reduces the dilution of delivered genetic material, aiding efficient transfection. The surface structures of the rodent eye, however, are poorly relatable to humans for trans-topical drug delivery due to differences in thickness and cell layering. Human corneas average 535±20 µm in the centre, increasing to 657±71 µm in the periphery, compared to 137±14 µm and 90.55±1.9 µm in C57BL/6 mice (Figure 9, Table 7), and have half the number of epithelial layers on the corneal surface (5-7 in humans compared to 13 in mice) 140. In addition, the small volume of the rodent vitreous limits the ability to assess the contribution of AS in the delivery molecules through the retina, as such, the included studies often replaced a portion, or all, of the vitreous with their chosen treatment, maximising the concentration and passive diffusion of molecules throughout the cavity. Thus, whilst useful in early proof-of-concept studies, rodents are not ideal models to justify the validity of clinical translation of a given ocular drug delivery technology.

#### 3.7.2. Rabbits

Rabbits are considered to have an ideal balance between function and accessibility in ocular studies; they require similar ethical approvals to rodents, are relatively inexpensive to purchase and maintain, and may be easily handled. In addition, their eyes are much larger than their rodent counterparts, at a vitreal volume of 1.15-1.5 ml compared to the 5.3 µl and 50-55 µl of mice and rats, respectively (Figure 9, Table 7) 146. With a corneal thickness approximately 370 µm centrally, thickening to ~450 µm toward the limbus, and an epithelial thickness of 30-40 µm, rabbit eyes corneas remain approximately 20-40% thinner than the human counterpart 143. Despite this, the exposed surfaces of the rabbit sclera are comparable in thickness to the human eye, at around 500 µm at the limbus, which progressively thins toward the posterior eye. The equator separating the anterior and posterior globe has a thickness of 250 µm superiorly and 200 µm inferiorly and the region near the optic nerve displays an average thickness of 180 µm 143. Comparatively, the human sclera thins from 500 µm at the limbus toward 420 µm at the equator, then thickens toward 860 µm outside the optic nerve head 147. Thus, trans-topical delivery in rabbits is most representative of the human standard near the limbus, with a risk of exaggerating the reported molecule delivery efficacy compared to what may be seen in humans toward the equator and further posteriorly. Irrespective of this, most trans-topical studies in rabbits delivered drug to the entire exposed surface of the eye including both corneal and exposed sclera components.

The rabbit's high accessibility and large external surface area has made them a popular choice in trans-topical ultrasound-mediated drug delivery. Of the included studies, 16 (43%) used rabbits, of which 13 assessed MBP_ENDO_-mediated trans-topical delivery of small molecules or contrast agents (Table 3). 10 of the 13 trans-topical studies were completed in *ex vivo* models and the remaining three in *in vivo* animals. Of the three *in vivo* studies, all utilised MBP_EXO_ for molecule delivery. One transfected corneal epithelium after direct injection of a plasmid/microbubble suspension 36, another delivered mNGF intravitreally with microbubbles and ultrasound to preserve the optic nerve in a model of ocular hypertension 46, and the third replaced an epiretinal portion of the vitreous with an intravitreally delivered plasmid/bubble liposome suspension followed by applying ultrasound toward the retina from an intravitreally implanted ultrasound transducer 65. The use of rabbits has allowed for the expansive testing of trans-topical delivery systems and has arguably progressed the field toward clinical application. However, despite this progress in trans-topical drug delivery, no clinical trials in humans have yet been identified.

#### 3.7.3. Large mammals

Large mammals, such as non-human primates, represent an ideal model for ocular drug delivery. Particularly the Rhesus and Cynomolgus monkeys, as, compared to all of the species used in the included studies, these monkey species have eyes which contain a macula and share common susceptibility genes (Rhesus) or gene orthologues (Cynomolgus) for age-related macular diseases 148, 149. This, in concert with their similar anatomical organisation and functionality capability to that of human eyes often makes them the best animal model for advancing fundamental knowledge and developing new treatments for sight-threatening diseases 150. The barriers to accessing non-human primates for research are understandably high; stringent research ethics, prohibitive costs to obtain and maintain, as well as difficulties in handling contribute to their limited use. In the included studies, researchers have opted instead for bovine or porcine eyes, for either trans-topical or vitreal/vitreoretinal delivery. Both bovine and porcine sclera are thicker from near the limbus (920±60 µm and 800 µm) toward the equator (646±43 µm and 560 µm) compared to human eyes (500 µm and 420 µm, respectively) (Figure 9, Table 7) 144. It should be noted the porcine sclera progressively thins to a thickness comparable to human sclera approximately 6mm behind the limbus, to a mean thickness of 430±130 µm, before thickening as it progresses posteriorly toward the equator. As such trans-topical delivery may more closely match human kinetics at this region. Due to the thicker sclera, trans-topical delivery studies using bovine or porcine eyes may underestimate the efficiency of molecule delivery into the eye, when compared to human use. Whilst not initially problematic, this may result in the optimised use of more intense sonication parameters than what may be required, increasing the risk of unnecessary tissue damage.

These large mammals exhibit high barriers to accessibility, due to the cost of purchasing and maintenance, their size, difficulties in handling and more rigorous ethics applications. All 5 studies using large mammal eyes overcame these barriers by using *ex vivo* eyes collected posthumously from abattoirs, although this entails its own limitations, discussed later in section 3.7.4 of this review. Of the 5 studies using large mammals, 2 each used bovine and porcine eyes, and one used both. The porcine-only studies delivered small molecules or fluorophores trans-topically, one using HIFU 49, and the other a planar transducer 43. Murugappan and Zhou (2014) delivered bicinchronic acid through porcine sclera using HIFU 49, and the other trans-topical study targeted the porcine cornea, comparing the magnitude of trans-topical riboflavin delivery with and without the epithelium 43.

The 2 studies using bovine eyes both dissected and removed the anterior segment and vitreous from the eye, applied their treatment suspension onto the epiretinal space and sonicated the tissue to promote delivery into the neural retina 40, 64. In an additional experiment, one of these studies investigated the delivery of nanoparticles through the vitreous under the influence of transscleral ultrasound in whole porcine eyes 40. This was then reassessed later using dye-loaded echogenic liposomes to improve the efficiency of particle delivery through the vitreous 42. These 2 experiments represent the only studies highlighting the use of AS to improve the delivery of intravitreally injected molecules in eyes large enough to overcome the contribution of passive diffusion, as is seen in rabbit and rodent eyes.

The pig eye has a smaller volume compared to the human, 3.3 ml compared to 4.4 ml, Whilst the bovine vitreal volume is greater, at 14.6 ml (Table 7, Table S3). The rheological properties of bovine and porcine vitreous appear similar to the human, although diffusion coefficient and steady-state flux values do not align 56.

Given the similarities in anatomical structure, size, and function, non-human primates are the ideal model for translational ocular research. However, given the barriers associated with their use, alternate species such as the cow or pig may be a preferred modality, with greater translational potential compared to rabbit or rodent studies. Both large mammal models may be particularly useful for vitreal/vitreoretinal delivery, whilst trans-topical studies may be of greater benefit in pigs. Given the lack of translation in ultrasound-mediated ocular drug delivery from the lab bench to clinic, there may be value in validating previously studied techniques from rodent or rabbit studies in large mammals, due to their closer comparability to human eyes.

#### 3.7.4. *Ex vivo* and *in vivo* studies

A criterion for studies to be included in this review was the use of *in vivo* or *ex vivo* animal experiments. Each model has various advantages and disadvantages associated with their barriers to entry, experimental applications, and translational relevance. *Ex vivo* experimentation is commonly used as an initial proof of concept for drug delivery research as it is cheaper, less time consuming, and often has a lower barrier to entry compared to *in vivo* studies. Despite this, safety and therapeutic efficacy assessments are often severely limited in *ex vivo* studies, with most of the included *ex vivo* studies primarily relying on immediate structural changes induced by ultrasound, with some reassessing structural changes within 2 hours of treatment. There are difficulties associated with maintaining *ex vivo* eyes, particularly due to the relatively isolated nature of the neural retina, which introduces challenges associated with nutrient delivery. Thus, *ex vivo* models are often limited to delivery efficiency assessments only, rather than delivery and subsequent efficacy of delivered molecules in a therapeutic model. Despite this, Balasubramanian and Shabanian (2020) recently demonstrated viable retinal responses from porcine eyes up to 4 days post-mortem, when stored with the surrounding orbital tissues attached, so perhaps this trend will change in the future 151. *In vivo* studies must maintain the animals in holding facilities, which incurs additional costs, handling issues and ethical approvals. However, the advantages are significant, allowing for a more complete assessment of changes to the eye, a better capacity for applications in gene delivery, and the ability to assess both the long-term safety and effect of repeated treatments on living tissue.

14 of the included studies assessed drug delivery only in *ex vivo* animal eyes, 21 only used *in vivo* animals*,* and 2 studies examined both. 11 of the 14 *ex vivo* studies investigated trans-topical drug delivery, 10 of which were completed in rabbits and 1 in pig eyes. The remaining three *ex vivo-*only studies investigated vitreal/vitreoretinal delivery in either cow or pig eyes. The preference for trans-topical ultrasound delivery in *ex vivo* rabbit models demonstrate that the efficacy of delivery is currently the primary outcome measure of these studies, rather than the investigation of ultrasound-mediated bioeffects or improved therapeutic outcomes.

Comparatively, of the 21 *in vivo*-only studies, 16 relied on rodents and the remaining 4 studies used rabbits. The large skew toward rodent usage in *in vivo* studies underscores the higher barrier to entry associated with maintaining larger live animals, which as outlined prior, are preferred models for human translation. Thus, the current scene for ultrasound-mediated ocular drug delivery experimentation is characterised by either *ex vivo* studies using more translatable rabbit or large mammal models, or *in vivo* studies using rodents with a lesser capacity for clinical translation.

### 3.8. Safety assessment

Any procedure applied to the eye needs to satisfy an acceptable risk/benefit analysis; therefore, studies investigating a novel drug delivery method need to couple their findings with appropriately chosen assessment of safety 152. These assessments should not only consider thermal and mechanical indices of safety, but should investigate structural, biochemical, and functional deviations from expected norms to fully characterise the effect of ultrasound on ocular tissue. Different structures in the eye have differing rates of cell turnover, capacity for regeneration, and propensity for scaring. In addition, side effects may develop at various time points depending on the underlying pathology, thus the timing of safety measures need to also be considered. The assessments of ultrasound effect on tissue health implemented by each included study are summarised in Table 8.

#### 3.8.1. Assessment timing

Post sonication, changes to the ocular surface appear rapidly reversible. The human corneal surface is composed of 5-7 layers of non-keratinised squamous stratified epithelial cells with an average turnover of 10 days. Desmosomes form tight junctions between superficial epithelial cells and maintain the differing basal and apical environments 51, thus ensuring the relatively dehydrated nature of the underlying stroma remains so, and minimising the risk of infectious ingress 153. It is these tight junctions which are hypothesised to be disrupted to allow ultrasound-mediated trans-topical hydrophilic drug delivery (compared to the underlying collagen matrix whose disruption appears to facilitate lipophilic drug delivery) 12.

The magnitude of improved trans-topical drug delivery has been correlated directly with corneal damage 16. In a study assessing trans-topical drug delivery in rabbits at lower intensities, ultrasound-induced epithelial disorganisation and dimpling of the superficial epithelium appeared to be reversible within 90 minutes post sonication 62. When another study compared safety assessments made at intermediate (> 60 min and ≤ 24 h) or long time (> 24 h) periods post sonication in rats, the presence of apoptotic scleral cells, determined by Terminal deoxynucleotidyl transferase dUTP nick end labelling (TUNEL) staining, decreased with time post sonication 28. The majority of included studies only assessed one time point for safety assessments, and of those that tested multiple time points, most did not repeat the same type of safety assessment. This is important, as the fast-healing nature of the eye may mask the magnitude of damage initially induced by treatment if assessments are delayed, underrepresenting the actual effect of sonication. When aggregating the histological assessments pertaining to safety, 66% of studies that assessed damage within 60 minutes had at least one treatment group displaying signs of damage 15, 39, 40, 42, 43, 49, 60-62, 69, 70, 74, whilst of the studies assessing damage after 60 minutes but within 24 hours 16, 28, 39, 66, 154, and those assessing damage after 24 hours 17, 28, 35-39, 65, 77, 82, 83, 60% and 9% reported damage (n = 12, 5, and 11, respectively). Whilst not a certain indicator of missing data, it may be argued the studies assessing damage after 24 hours post sonication likely missed initial evidence of damage, whilst those only investigating damage directly post-sonication lost the opportunity to report on any potential recovery (or lack thereof) in the following hours and days. For *in vivo* studies, a longitudinal assessment of multiple time periods after sonication, for instance at times t = 0, 60 min, 24 hr and 7-10 days would provide a more comprehensive assessment of ultrasound-induced structural changes, and their chronic effects on eye health.

#### 3.8.2. Mechanical index

The MI describes the likelihood of cavitation occurring in a medium exposed to ultrasound. Stable cavitation is more likely to occur at lower MI values, whilst inertial cavitation, whereby the bubble collapses violently, is a consequence of ultrasound application at higher MI values. Inertial cavitation results in high temperatures, microjet formation and shock waves delivered into the surrounding medium 155. These forces can result in deleterious adverse events ranging from microscopic cellular changes such as membrane perforation and cytoskeleton rupture, to larger architectural changes such as microvascular haemorrhage 156. Given the severe consequences the mechanical effects of ultrasound may impart onto tissue, maximum MI values have been set by the medical safety authorities of various countries, including the US-FDA and the British Medical Ultrasound Society (BMUS), to a value of ≤ 0.23 and ≤ 0.3, respectively, for ophthalmic ultrasound applications 63, 157.

Of the included studies, only 9 either recorded the PNP of the delivered ultrasound or stated a calculated MI. Of these, 5 assessed indices below 0.3 39, 60-62, 74, 3 assessed MIs above 0.3 15, 49, 66, and 1 assessed a range both above and below 43. Those studies which assessed a range of MIs above 0.3 reported an increase in deleterious structural effects as the MI increased. These included extravasations of erythrocytes into the retinal layers, erosion of the corneal surface and disruption of retinal layer organisation 15, 49, 66. Comparatively, studies which assessed MIs below 0.3 generally found either nil adverse effects, or reversible changes on histology, such as corneal pitting which disappeared by 90 minutes post sonication 39, 60-62, 74. Similarly, the one study which assessed a range which crossed from below to above an MI of 0.3 showed nil tissue changes at an MI of 0.2, but some disruption of the corneal epithelium at 0.4, and extensive epithelial debridement at an MI of 0.8 43. Unfortunately, there is also a trend in improved drug delivery as the MI increases, thus a balance needs to be found between cell injury and treatment efficacy. Given the current safety standards of ophthalmic ultrasound set out by medical safety authorities, any intended treatment with a MI above 0.3 would have additional barriers to clinical translation compared to those using lower indices.

##### 3.8.2.1. Cellular and immunological aspects of mechanical ultrasound effects

Mechanical interactions with cellular components need to be considered to understand the risks associated with ultrasound application. Cavitation, both stable and inertial, acts to permeabilise, or sonoporate, cell membranes and is a primary underlying mechanism by which genes are delivered to *in vitro* and *in vivo* cell populations 154, 158. The degree of cell damage from sonoporation is often found to be proportional to the magnitude and duration of cavitation, whereby stable cavitation appears to induce mostly reversible holes in cell membranes, whilst inertial cavitation significantly increases the rate of cell death through widespread mechanical lysis of the cell 156, 159.

Stable cavitation, characterised by the cyclical contraction and expansion of gas bubbles, induces radiation force- and microstreaming-borne shear forces against cellular membranes and intercellular connections. Comparatively, the violent collapse and fragmentation of microbubbles undergoing inertial cavitation creates microjets and/or shockwaves which are capable of puncturing cell membranes and rupturing the underlying cell cytoskeleton 156. In vascular populations, the deleterious effects of extensive cell lysis and loss of intercellular adhesion may result in macroscopic vessel perforation and intraparenchymal bleeding. Importantly, the addition of microbubbles as a source of cavitation nuclei significantly increases the efficiency of acoustic energy conversion into mechanical energy, and thus the risk of damage to nearby cell populations significantly increases proportional to the amount of gas delivered to the tissue. The sonication pressure required to induce cavitation is inversely proportional to the microbubble concentration 124, and the magnitude of cavitation is directly proportional to both the mean size and concentration of the delivered microbubble population 122, 123. Therefore, great care should be taken when considering the choice of microbubble, and by what method it is administered, as rupture of the vasculature within the globe would have severe consequences reaching beyond the immediate intraparenchymal bleeding.

The eye is an immune-privileged organ bereft of a lymphatic system, although the surrounding conjunctiva has extensive lymphatic drainage 160. Therefore, during the initial months and years of development, antigens specific to intraocular tissue are never presented to the developing immune system to be recognised as “self.” As a result, any break in the barriers maintaining this immune-privileged state increases the risk of immunoreactivity to autoantigens present within the eye. This can trigger the immune system to recognise both eyes as foreign, resulting in a sight threatening chronic granulomatous pan-uveitis, termed sympathetic ophthalmia 160. Sympathetic ophthalmia occurs almost always after a penetrating ocular injury, or ocular surgery and, whilst not currently associated with ophthalmic ultrasound-mediated drug delivery, the mechanisms of BRB-targeted drug delivery may increase this risk, whereby incidental perforation of the vascular bed creates a potential opening for unwanted immune surveillance.

#### 3.8.3. Thermal index and thermometry

Thermometry is integral to safety assessments throughout all applications of ultrasonography. Temperature effects of ultrasound may be modelled using the Thermal Index (TI), which indicates the potential for heating in various tissues during sonication. A maximum TI of 1 has been both approved by the US-FDA and suggested by BMUS for use in ocular ultrasound, indicating that the applied ultrasound intensities should not likely increase the temperatures of ocular structures by more than 1 °C 63, 157. Sonicated tissue will absorb a proportion of the delivered acoustic energy as heat, which may cause damage if allowed to accumulate. In ocular tissue, the lens, cornea, aqueous humour, and vitreous humour are particularly sensitive to overheating due to the minimal blood flow available to dissipate heat 104. In addition, the dissipation of heat is impeded in these components of the globe, therefore elevated tissue temperature may extend beyond the cessation of sonication.

##### 3.8.3.1. Cellular and molecular aspects of thermal ultrasound effects

Ultrasound intensity is attenuated as it passes through tissues, and the acoustic energy is converted into thermal energy 161. The degree of thermal injury to tissue is dependent both on the magnitude of the increase in temperature above physiological values, and the duration for which those temperatures are applied 162. The time required to cause thermal death of *in vivo* tissues appears to decrease exponentially above temperatures of 40 ºC, to the extent that the lethal dose of exposure at 40 ºC may be measured in hours, whilst the lethal dose of 46 ºC is measured in minutes 163.

Deleterious effects due to ultrasound irradiation can range from mild temperature increase to coagulative necrosis and tissue vaporisation, depending on the type of transducer and parameters used 10. Elevations in ocular temperature due to prolonged exposure to ultrasound may result in increased denaturation of plasma membrane proteins, an increase in plasma membrane fluidity, and subsequent cell lysis 43. In addition, high intensity ultrasound has induced cataracts, corneal clouding, and lens opacification in humans, and as such these adverse effects should be assessed post-sonication 104, 164. Toxic *in vitro* thermal effects become apparent between 39 to 40 ºC, whilst coagulation of proteins occurs between 44 to 46 ºC and the protein constitutes of enzymes become denatured at approximately 50 ºC 163.

##### 3.8.3.2. Measuring and applying the thermal index

The TI is defined as the ratio of a device's output acoustic power to the power required to raise the tissue's temperature by 1 °C 24, 161:


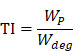

(30)

where 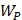 is the relevant (derated) acoustic power emitted by the transducer at the depth of interest, and 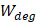 is the power required to raise the temperature of the tissue by 1 °C.

Acoustic power, 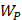, is measured using the derated bounded-square output power, 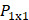, which is the peak intensity within a 1x1 cm square of the sonication beam measured perpendicular to the direction of the delivered wave 63, 165. A difficulty found in the use of TI includes determining an accurate 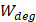 value for the target tissue, as the rate of heating of different tissues will differ based not only on properties of absorbance particular to the tissue, but also the magnitude and velocity of blood flowing through the tissue, which acts to efficiently dissipate heat. Thermal index is a measure of relative risk to the patient which, whilst providing a useful guide, does not represent the absolute thermal risk to exposed tissue, which requires an accurate measurement of temperature rise in targeted tissues 161.

The Soft Tissue Thermal Index Model (TIS) is commonly used for ophthalmic ultrasound 63. Designed for abdominal tissue, this model assumes the sonicated area is homogenous, has low fat content, does not contain large gas-filled spaces or calcifications, and the attenuation coefficient of the tissue, 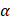, is equal to 0.3 dB cm^-1^ MHz^-1^ 162. Its calculation is dependent on whether the transducer is using a scanning or non-scanning beam; since only non-scanning beams have been used for ocular drug delivery, only the relevant equations will be discussed. For TI calculations, the full derivation and rationale may be found elsewhere 166. The model used for non-scanning ultrasound is dependent on the area of the transducer tip. For small, < 1 cm^2^ transducers, the location of maximum heating is assumed to be near the surface, as expected considering the relationship between the Fresnel zone length and transducer diameter given in equation (19). The TIS equation for small transducers is 161:


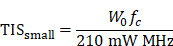

(31)

where 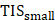 denotes the thermal index equation of a non-scanning transducer of aperture < 1 cm^2^, 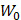 describes the total ultrasound output power from the ultrasound source in mW, and the denominator, 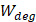 is equal to 210 mW MHz as experimentally determined by Curley (1993) 167.

Comparatively, when considering transducers where the initial beam width is larger (>1 cm^2^), the location of maximum risk from thermal mechanisms is assumed to be deeper in tissue, therefore TI is expressed as 161:


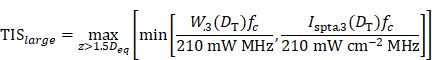

(32)

where the output power, 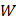, and 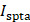 are measured in water at a distance, 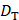, then derated assuming a tissue attenuation of 0.3 dB cm^-1^ MHz^-1^, to become 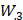 and 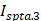, respectively. 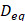 is the equivalent aperture diameter defined by 161:


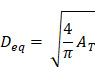

(33)

where 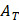 is the transducer area?

A minimum depth of 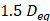 is used to attempt to avoid measuring 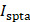 within the near field of the source, where intersecting acoustic wavelets would introduce inaccuracies in the pressure measurement.

Thus, the TI of non-scanning ultrasound transducers used in ocular drug delivery may be calculated using the established soft tissue models. This model, however, fails to consider the eye does not have homogenous attenuation throughout, rather the separate structures of the eye will attenuate the acoustic wave at different rates, and therefore heat at differing rates. The choroid, for instance, has an acoustic attenuation of 0.5 dB cm^-1^ MHz^-1^ (Table 5), but has the greatest vascular flow per gram of any body tissue, minimising the rate of heating expected in this tissue 54. Comparatively, the lens, which has an appreciable mean thickness of 4-4.7 mm (increasing in thickness from 20-60 years), has a higher attenuation coefficient of 1.38 dB cm^-1^ MHz^-1^ (Table 5) 168, 169. The lens is avascular, lacking the capacity to remove heat efficiently, and has been shown to develop cataracts under the exposure of erroneously targeted HIFU in humans 164, and clouding after excessive sonication from a planar transducer in *ex vivo* bovine eyes 42. So, using the TIS model to determine safety limits in the eye may not be sufficient to ensure tissue safety. Rather, a model which considers the individual attenuation of each tissue within the eye and gives the TI of each tissue (using separate tissue groups for trans-corneal application and trans-scleral application) would give a more accurate understanding of the heating expected within each tissue.

The eye has an additional structural aspect alienating it from the assumptions made by the TIS model, whereby it is enclosed in an orbit of bone. Bone heats rapidly compared to soft tissue 161, and the optic nerve must travel through a tunnel of bone, the optic chiasm, to reach the brain. Any heating of surrounding bone may quickly heat the nerve beyond what may be expected with the currently used TIS model.

An updated TI model more representative of the eye would be of benefit to both clinical and experimental ocular ultrasonography. Even so, the TI model only describes the relative likelihood of thermal accumulation in a tissue, compared to alternate parameters applied for the same duration. The 'dose' of ultrasound over time is not informed by the TI calculation, as such ultrasound applied using a TI of 0.1 may result in greater absolute tissue heating if applied for longer than a transducer applied using a TI of 0.4; lower TIs have the risk of giving a the operator a false sense of safety, particularly in ocular ultrasound 161. The model also does not consider the contribution of transducer heating, which has been demonstrated by Lamy *et al*. (2018) to as much as double the final ocular surface temperatures compared to when active cooling of the transducer tip is used (6.2 °C vs 3 °C, respectively) 41.

14 of the included studies (38%) included thermometry to assess the safety of their chosen ultrasound parameters, of which 12 assessed trans-topical delivery (Table 8). Eleven of the trans-topical studies applied continuous ultrasound, one of which measured an increased average temperature of 2-3 °C when testing frequencies of 0.4-1 MHz and intensities of 0.3-1 W/cm^2^ applied continuously for 5 minutes but did not specify which tested parameter resulted in this temperature increase 70. Two studies directly compared temperature changes at differing sonication frequencies; Nabili *et al*. (2014) found that decreasing the frequency from 0.6 MHz to 0.4 MHz at 0.8 W/cm^2^ for 5 minutes resulted in an average increase of 2.2 °C in the sclera by treatment end (1.8 °C and 4 °C, respectively) 16, whilst Chau *et al*. (2017) showed no significant change in tissue temperature when applying ultrasound from 0.04 MHz to 3 MHz for 30 s at 0.05 W/cm^2^, highlighting the additional importance of sonication duration and ultrasound power on absolute increases in temperature 72.

Chung (2019) tested the effect of increasing sonication power and duration on temperature rise using 40kHz continuous ultrasound and found that both contributed to an increase in final temperature. The change in temperature from baseline increased from 0.1 °C to 12.7 °C when increasing the *I*_SA_ from 0.07 W/cm^2^ to 1.22 W/cm^2^ after 10 minutes of sonication. Whilst the change in temperature at 1.22 W/cm^2^ from 10 to 30 minutes increased from 12.7 °C to 17.4 °C 43.

When continuous ultrasound was applied for five minutes or more, at powers equal to or above 0.5 W/cm^2^ and 880 kHz, there was a significant rise in tissue temperatures ranging from 6.2 °C to 9 °C 41, 60, 62, 71. One study suggested 1 MHz ultrasound applied continuously for 5 minutes at 0.5 W/cm^2^ does not significantly raise the surface temperature of the scleral surface, however their measurement methodology - an infrared thermometer measured before and after treatment - may suffer from additional uncertainties compared to the more commonly used *in situ* thermocouples 74. Lower powers and sonication durations did not significantly increase temperature when applied using 40 kHz ultrasound at 0.12 W/cm^2^ for 90 s 39, or using 1 MHz ultrasound at 0.05 W/cm^2^ for 30 s 61. When considering intermittent ultrasound, Kowalczukk *et al*. (2011) showed that pulsed ultrasound (50 % duty cycle, 100 Hz PRF) applied using 1 MHz ultrasound and an *I*_SATA_ of 2 W/cm^2^ for 2 minutes resulted in a mean increase in the lens and ciliary region of sonicated eyes by 3.7 °C and 7.3 °C, respectively 28.

Another study assessed thermometry whilst using ultrasound for trans-vitreal and vitreoretinal nanoparticle delivery to bovine retina. They showed that their working parameters of continuous ultrasound applied at 1 MHz using a power of 0.5 W/cm^2^ for 30 seconds resulted in a mean increase in scleral surface temperatures of 1.5 °C. The researchers then compared this to a positive control using 2 W/cm^2^ for 30 seconds, increasing scleral temperatures by 3 °C compared to non-sonicated controls 40.

A large limitation in the methods used in most of the included studies was the measurement of temperature only in the target tissue where drug was being delivered. No *in vivo* trans-topical drug delivery study assessed the impact of ultrasound on tissue behind the ocular surface, despite the consideration that internal tissues may be heating to a greater extent compared to the surface. Finally, the use of thermometry in these studies highlight the necessity for accurate temperature assessments. Both the sensitive nature of ocular tissue and the strict TI limiting ultrasound parameters supports the need for highly accurate probes used in temperature measurements. Equipment used for thermometry should have an accuracy uncertainty below ±1 °C and would ideally be within at least one order of magnitude less than that (< ±0.1 °C). This limits the preferred probes used for thermometry to platinum resistance thermometers, thermistors, and some specifically calibrated thermocouples. In addition, published studies should provide the make, model and accuracy of the probe used.

#### 3.8.4. Macroscopic assessments

The effects of excessive sonication can be obvious. When Xie *et al*. (2010), applied a total of 20 seconds of continuous ultrasound at intensities of 2.0 or 2.5 W/cm^2^, mice experienced a delayed vitreous haemorrhage three days post sonication severe enough to be visible to the naked eye 33.

A more effective measure of gross changes to ocular structures may be assessed using slit lamps to investigate both anterior and posterior segment health. These methods may be used in both *in vivo* and *ex vivo* experimental designs but are of particular use when tracking gross eye health over the course of multiple time periods post sonication in living subjects. Ultrasound-induced inflammation of the anterior segment can precipitate breakdown of the blood-aqueous barrier, resulting in an increase in the number of cells and protein in the aqueous humour 170. Slit lamp biomicroscopy is an ideal, although not readily available, ocular assessment tool, and has been used to assess for hypopyon, preretinal haemorrhage and changes in bulbar shape both acutely and chronically post sonication in rabbits 65, 74. The slit lamp, using a cobalt blue filter in combination with fluorescein administration, is also the ideal tool for assessing the integrity of the superficial layers of the eye, which may be altered by ultrasound. Fundoscopy, can be conducted to examine the posterior segment, and should include an assessment of endophthalmitis (particularly after intravitreal injection), vitreal opacity, retinal detachment, haemorrhage, or oedema 39, 74.

#### 3.8.5. Microscopic assessments

Histological examination can be used to assess structural and functional changes to sonicated tissue. Light microscopy alone has been used to assess structural changes in corneal epithelium 36, 62, and was able to detect the presence of corneal pits directly post sonication, which receded within 90 minutes 62. Whilst light microscopy is useful, greater details pertaining to cell populations, morphology and architecture can be gathered using common gold standard structural stains such as H&E. 22 of the included studies assessed the structural changes caused by sonication using H&E, whilst one study each used Richardson's stain (Methylene blue & azure II) 60, and toluidine blue (Table 8) 28.The most common result of sonication was disruption of the superficial corneal or conjunctival epithelial layers, although as mentioned prior, *in vivo* safety assessments that were delayed beyond 24 hours tended to reveal no significant changes under H&E. When assessed immediately, the magnitude of structural abnormality appeared to increase inversely proportionally to frequency, and proportionally to power, PNP, sonication duration and pulse duration (Table 4). More stringent structural assessments such as TEM and/or SEM confirmed transcorneal ultrasound may create holes in the epithelium due to individual cell sloughing and bulk cell removal, as well as induce structural signs of cell apoptosis (hypopigmentation and swelling) 49, 60. One study assessed ultrasound induced changes to scleral collagen networks using second harmonic generation (SHG) imaging. The imaging technique demonstrated a resolution capable of distinguishing individual collagen fibres and showed the range of ultrasound parameters they tested (*I*_SATA_ 0.002-1.8 W/cm^2^, 40 kHz, continuous for 30s) did not alter scleral collagen arrangement 73.

Cell-specific staining has been used to detect the infiltration of blood-borne cells through the BRB. This has been used primarily as an indicator of extensive vascular endothelial damage during retinal sonication after intravenous delivery of microbubbles for trans-BRB drug delivery. In particular, both erythrocytes and megakaryocytes have been stained for using anti-Ter119 and anti-CD41, respectively 15. In this study, 120 10 ms pulses of focused ultrasound using 1.1 MHz and 0.36-0.84 MPa PNP directed to the retina resulted in no extravasation of CD-41 positive cells after 30 minutes, but positive staining for erythrocytes. In addition, the presence of the clotting factor fibrinogen was confirmed upon antibody staining as well. It should be noted the antibody used is non-specific for fibrinogen, fibrin or the degradation product fragments D and E, so the presence of active clotting cannot be confirmed with confidence 171. In addition, since megakaryocytes are vanishingly rare in peripheral blood circulation, the use of anti-CD41 was likely testing for platelets, despite the author's claims toward testing for megakaryocyte infiltration. Platelet staining appeared negative, which is unusual given the presence of both clotting factors and the 5 x larger erythrocyte cells 15.

The assessment of structural changes and cell extravasation are useful and clear markers of ultrasound-induced ocular damage. A third category of microscopic analysis included functional changes in cell behaviour associated with damage responses. Two common stains for cell viability used in the included studies included trypan blue and TUNEL staining. Trypan blue has shown a lack of ultrasound-induced cell death up to 12 hours post sonication, when applied using parameters of 1MHz at 2 W/cm^2^, 100 Hz PRF and 50% DC for 300 seconds 154. This stain assesses changes in cell permeability, and, given that these changes occur toward the final stages of apoptosis, it has been shown to underestimate the number of non-viable cells in corneal epithelium by up to 8-fold compared to TUNEL staining in a comparative study using corneal epithelium 172. Another included study assessed cell apoptosis using TUNEL staining on day 1 and 8 post transscleral sonication of ciliary muscle injected with a combination of microbubbles and plasmid. This study found significant apoptosis at the injection site in both sonicated and non-sonicated eyes, which reduced to sparse positive staining in both groups by day 8. Sonicated ciliary muscle showed comparable results, albeit at a lower magnitude at both time points compared to the injection site 28.

An additional functional stain indicating an inflammatory response assesses for the presence of GFAP amongst retinal cell populations, indicating reactive gliosis of müller cells, which respond to cell injury. Whilst moderate gliosis is transient and protective, severe gliosis is cytotoxic, and may scar and remodel the retina permanently 173. The same study which used anti-CD41 staining also assessed GFAP expression in retinal regions using the prior-outlined sonication parameters 15. The researchers assessed GFAP upregulation only 30 minutes post sonication and found 1/6 retinas were positive. The researchers concluded the retinal injury response is delayed after focused ultrasound application, however since GFAP upregulation in retinal müller cells relies on protein expression, a negative signal within 30 minutes would be expected. The researchers did not assess GFAP upregulation at any later time point. Other studies in the literature have demonstrated GFAP upregulation and expression occurs after 1 hour post ischemia-hypoxia injury, within 2 hours post intraocular pressure rise, and 4-5 hours post axotomy of retinal ganglion cells 173. Thus, the protocol used by Touahri *et al*. (2020) to assess GFAP upregulation would have been unlikely to capture the window of upregulation, if any, post sonication. This was the only study using GFAP, CD41 and TER119 staining to assess ultrasound-induced damage. Further studies using stains for cellular extravasation and intraocular inflammation need to be carried out before ultrasound used for these purposes may be considered safe.

A key aspect of the histological assessments which need to be considered include the difficulties associated with tissue preparation. The primarily aqueous nature of the vitreous tends to promote tissue shrinkage and increased traction forces on the internal eye, particularly during the process of tissue dehydration and paraffin embedding. This may lead to artefactual retinal detachment. Comparatively, preserving the tissue via freezing may result in globe rupture due to expansion of the vitreous, and slower freezing techniques involving sucrose infiltration may also result in retinal detachment due to osmotic forces between the retina and retinal pigment epithelium. These factors need to be considered during structural analysis post treatment, as there is the risk of mistaking ultrasound-induced retinal detachment for tissue processing artefacts, and vice versa. Blinding of outcome assessors may help reduce the bias associated with reporting these factors.

#### 3.8.6. Electrophysiological assessments

A primary concern in safety associated with experimental ophthalmic ultrasound involves the risk to visual acuity. Structural and biochemical cellular changes may impart information on the likelihood of ultrasound causing deleterious effects of visual function, but direct assessment of visual acuity in animals is difficult. Broadly, there are two internationally recognised visual acuity standards for testing rodents; these include behavioural or electrophysiological visual acuity tests, which have been extensively reviewed elsewhere 174.

Only electrophysiological tests have been used in the included studies, namely full-field electroretinography (ffERG) 46, and flash visual evoked potential measurements (fVEP) 39. These tests are particularly useful in translational research, since similar methodologies may be used for both laboratory animals and humans, and human retinal pathologies such as AMD share phenotypic commonalities with animal disease models 175. Essentially every region of the primary visual pathways, and any aspects of vision, from a single cell to a network level, may be tested using electrophysiology 174. ffERG relies on electrodes applied to the corneal surface, followed by a flash emitted by a controlled light source in either a light or dark-adapted setting. The electrode measures the electrophysiological response formed by the changes in ion currents within the photoreceptors, bipolar cells, müller glia cells and RPE 176. ffERG, was used by Suen *et al*. (2013) to assess the safety of transscleral delivery of dextrans to the posterior segment in rabbits. The researchers found there was no change in visual acuity at day 1, 7 and 14 post sonication compared to measurements taken 4 days prior to treatment 39. It should be noted that no study assessed retinal activity during sonication, which is relevant, as ultrasound has been shown to stimulate the neurons of blinded rats, resulting in visual cortex activity 177. An alternate method of electrophysiological assessment, ffVEP, has been conducted to assess treatment efficacy in a separate study, which will be further discussed in section 3.9 46. Testing the neuronal activity and pathways of the visual system in response to experimental ultrasound is a direct and vital measure of treatment safety and may improve the capacity for efficient clinical translation of these methods.

### 3.9. Assessment of drug delivery

Multiple methods assessing the degree of drug delivery have been used in the included studies, which are separated both by the *in/ex vivo* model and the mechanism of delivery. Trans-topical delivery, which primarily relies on *ex vivo* models, assessed either diffusion of tracer molecules through tissue by means of a Franz diffusion cell, or used whole eye explants. These studies either measured the depth of delivery through tissue or the concentration in the anterior or posterior eye segments. Comparatively, vitreal/vitreoretinal and BRB delivery studies primarily relied on *in vivo* models, which allowed both investigations into tracer molecule delivery and assessments of therapeutic outcome. Experiments assessing direct delivery into target tissue primarily relied on plasmid expression as a measure of treatment efficacy (Table 9). When considering the method of assessment, studies involving plasmid expression assessed delivery success primarily using quantitative microscopy, gene- or gene product-quantifying techniques such as real-time polymerase chain reaction (RT-PCR), Western blot, or enzyme-linked immunosorbent assay (ELISA). A minority of studies relied on qualitative scoring using masked observers. Studies utilising tracer molecules such as fluorescent dyes, nanoparticles, dextrans or drugs to demonstrate improved delivery primarily relied on spectrophotometry and quantitative microscopy, with a minority using flow cytometry. Finally, the two studies assessing a therapeutic outcome of a delivered molecule used either quantitative microscopy or electrophysiology measurements (Table 9).

#### 3.9.1. Tracer molecules

Tracer molecules, such as fluorescent dyes and drugs, have been used in all routes of ultrasound-mediated drug delivery to demonstrate treatment efficacy, whilst BRB-mediated delivery has also utilised MRI contrast imaging. Different tracers exhibit differing physicochemical properties, such as size, degree of hydro- or lipophilicity, charge, and the molecule's reactivity to local tissue structures Each of these properties will alter the efficiency of delivery depending on both the route chosen and ultrasound parameters used. Whilst sporadic, some studies assessed the effect of changing at least one of these physicochemical variables on delivery efficiency. The inclusion of ultrasound alters the physical environment to improve molecule delivery, as such typical trends in ocular penetration characteristics, may differ as outlined in Table 10.

Assessments of the effect of differing molecule charge and lipophilicity have been studied in transscleral studies. The effect of differing lipophilicity was investigated by Zderic *et al*. (2002), who used four beta blockers of increasing LogP (atenolol, carteolol, timolol and betaxolol with LogP coefficients of 0.57, 1.1, 1.8 and 2.81, respectively) in combination with ultrasound. The researchers used rabbit corneas in a Franz-diffusion cell arrangement with a 60 minute coincubation period 69. Betaxolol exhibited a significantly increased permeability after ultrasound compared to less lipophilic beta blockers, although the permeability of atenolol and carteolol was found to be greater than timolol (Table 10). Comparatively, Nabili *et al*. (2013), assessed the transcorneal delivery of three compounds, sodium fluorescein, tobramycin and the hydrophilic ester prodrug, dexamethasone sodium phosphate (DSP), of differing lipophobicity (LogP -0.67, -5.8 and 1.64), using various ultrasound frequencies and intensities 70. The researchers measured the concentration of drug in the receiver compartment after a 60-minute coincubation period, which began with 5 minutes of continuous ultrasound. Tobramycin delivery was unable to be improved significantly using ultrasound wherein the non-sonication control permeated at a mean rate of 25 x 10^-7^ cm/s, with ultrasound improving the permeability by 46.9% to a rate of 36.7 cm x 10^-7^ cm/s (ns). Comparably, sodium fluorescein and DSP demonstrated poor initial permeability of 5 x 10^-7^ cm/s and 0.11 x 10^-7^ cm/s, respectively, and after sonication, these two latter drugs demonstrated significantly improved permeability of 12 x 10^-7^ cm/s and 0.25 x 10^-7^ cm/s, respectively (an increase of 240% and 227%, Table 10). Whilst the difference in lipophilicity may have contributed to these differences in permeability, it should be highlighted the physiological charge differed between drugs; sodium fluorescein and DSP exhibit a charge of -2, and tobramycin displays a charge of +5. This is relevant because positively charges moieties have demonstrated impeded transscleral permeability due to the presence of negatively charged proteoglycans within the scleral matrix, which act to bind positively charged molecules 55.

The transport barriers of the eye also impede molecule delivery based on size and shape. As expected, proteoglycans, intercellular adhesion molecules, and the collagen and elastin matrix all work to impede larger weight molecules more than smaller ones 55, 178. Interestingly, studies have highlighted the importance of molecule shape over size as a primary contributor to permeability, whereby higher weight globular molecules diffuse more readily than more linear dextrans of similar or lesser molecular weight 50, 179. In ultrasound-mediated drug delivery studies, Chau *et al*. (2017) has shown that lower molecular weight dextrans penetrate through the sclera more easily with and without ultrasound 72. The researchers demonstrated that 20 kDa, 70 kDa and 150 kDa FITC-labelled dextrans may diffuse on average 100 µm, 60 µm and 50 µm, respectively, after 15 minutes coincubation. When applying ultrasound, the size-dependence was maintained, but dextran delivery was significantly improved, with 20 kDa dextrans capable of penetrating the entire 360 µm thick sclera after 30 s of ultrasound and a 15-minute coincubation, whilst 70 kDa and 150 kDa dextrans penetrated 304 µm and 178 µm, respectively (Table 10). Similarly, when considering vitreal/vitreoretinal molecule delivery, Peeters *et al*. (2008) showed the degree of ultrasound-mediated improvement for delivery depended on the diameter of their PEGylated polystyrene nanospheres 64. Sonication for 30 s, followed by a 2-4-hour coincubation allowed 52 nm nanospheres placed within the cow eye-cup to achieve a 17-fold improvement in RPE cell delivery through the neural retina compared to non-sonicated controls. Comparably, 131 nm nanospheres required 120 s sonication to achieve a 9.4-fold improvement, and no safe duration of ultrasound could improve the penetration of larger 218 nm nanospheres (Table 10).

#### 3.9.2. Plasmids

Therapeutic plasmid delivery as a novel treatment modality has its roots in the ever-growing understanding of the human genome and the underlying genetic aberrations now associated with previously incurable diseases, such as Parkinson's and Alzheimer's 180. The ability to alter the root cause of a disease to modify its activity or progression is an attractive therapeutic goal, evidenced by the over 3000 completed or ongoing gene therapy trials reported worldwide 181. The difficulty of developing a successful therapy appropriate for treatment may, therefore, be highlighted by the comparatively small number of approved gene-therapy drugs/products, which totalled 33 in mid-2021 181. Commonly cited challenges to successful translation to the clinic include their anionic charge, susceptibility to enzymatic degradation in the bloodstream and tissues, their inherent immunogenicity and their inhibited capacity to be delivered to the correct cell population 182. Ultrasound has been used in laboratory settings for the efficient transfection of *in vitro* cell lines at a rate of up to 200,000 cells/min, with over 80% viability after 72 h, with a population-dependent transfection efficiency ranging from 15-62% 158.

Ultrasound appears to facilitate gene delivery through cavitation-induced shear stresses which improve membrane permeability, creating temporary pores with which genetic material may enter 159. In *in vivo* applications, ultrasound has the additional advantage of enabling both local targeting and temporal delivery of gene therapies directly into the target tissue. In the case of ocular drug delivery, ultrasound typically improved the expression of delivered genes, which were primarily fluorescent proteins enabling quantitative microscopy or qualitative score-based assessments of transfection efficiency (Table 8).

#### 3.9.3. Electrophysiology

Evaluations of electrophysiology have been described previously, where it was used in safety assessment studies in the form of electroretinography to ensure the applied ultrasound did not impact the function of treated retinas. In addition to this methodology, a study has relied on flash visual evoked potentials (fVEP) to assess treatment efficacy of mNGF to protect neuronal function in an intraocular hypertension rabbit model 46. This method relies on a recording electrode placed above the inion of the skull (posteriorly), with ground and reference electrodes placed at the skull apex and forehead, respectively. The recording electrode may then measure the electrical activity of the visual pathway in response to unpatterned flashing lights or patterned images 183. Shen *et al*. (2016) assessed the neuroprotective effect of mNGF in rabbits with induced ocular hypertension. The researchers delivered mNGF via intravitreal injection, with or without ultrasound and/or microbubbles, once weekly, for three treatments starting one week post hypertension induction. After 4 weeks the researchers completed fVEP assessments, demonstrating that the combination of mNGF, ultrasound and microbubbles significantly reduced latency and increased signal amplitude compared to the hypertension control (63.8 ± 8.35 ms and 11.37 ± 2.84 nV compared to 125.00 ± 18.70 ms and 5.5 ± 3.03 nV, respectively), and was closer to normotensive control values (46.20 ± 6.90 ms and 15.9 ± 2.48 nV, respectively). Importantly, ocular electrophysiology assessments utilise similar methods between humans and animals 174, and diseases with retinal pathologies such as AMD, Alzheimer's disease, Huntington's disease and Neuronal Ceroid Lipofuscinosis all present similar electrophysiology profiles between humans and animals 175. These factors may help facilitate a continuity of research whereby the same techniques used to assess efficacy in animals may be used in initial human trials. Finally, Electrophysiology assessments of porcine retina has successfully demonstrated retinal viability 4 days post-mortem, opening the possibility of using these assessments in *ex vivo* experimentation in larger animals 151.

#### 3.9.4. Therapeutic outcome

Ultrasound-mediated drug delivery aims to improve the efficiency of delivered drugs, with the overarching goal to improve the treatment of ocular diseases. Studies assessing disease outcome directly have the advantage of demonstrating this end purpose. These studies are often conducted in disease models that are either currently difficult or impossible to treat, such as retinoblastoma, or highly costly, such as CNV. All the studies investigating disease outcome used rodent *in vivo* models. Two studies developed CNV models in rats by inducing damage to Bruch's membrane using lasers, confirmed by fluorescein fundus angiography (FFA) 75, 76. Both studies rescued the CNV pathology using PEDF delivered either in the form of plasmid transfection, or delivery of PEDF-loaded liposomes or PEDF-loaded VEGFR-2-binding immunoliposomes. Zhou *et al*. (2009) confirmed transfection using RT-PCR and Western blot, and assessed CNV progression at days 7, 14 and 28 post-treatment (which began 14 days post CNV induction) using FFA. Fluorescein leakage was significantly reduced in all ultrasound-treated groups compared to controls at all time points (optical density around 70, 60 and 50 on days 7, 14 and 28, respectively, in treatment groups, compared to 100, 120 and 130 in controls) 75. Comparatively, Li *et al* (2010) demonstrated a significantly reduced CNV area in ultrasound-treated rats compared to non-sonicated controls on day 7 of treatment (which began 7 days post CNV induction), whereby sonicated PEDF-loaded immunoliposomes significantly reduced mean CNV area compared to sonicated PEDF-loaded liposomes, non-sonicated PEDF-treated controls, and nil treatment controls (2.23 ± 1.38 µm^2^, 12.84 ± 4.62 µm^2^, 22.83 ± 4.74 µm^2^ and 38.46 ± 4.27 µm^2^, respectively) 76.

Similar to CNV, proliferative vitreoretinopathy (PVR) is characterised by uncontrolled cell growth and infiltration, but of RPE cells, müller cells, fibroblasts and macrophages rather than vascular endothelium 184. Zheng *et al*. (2012) induced PVR in rats using an intravitreal injection of platelet-rich plasma containing RPE-J cells, then, three days following, delivered siRNA targeting tissue growth factor-β2 (TGF-β2), PDGF, or both, via intravitreal injection, with or without ultrasound 34. The researchers assessed therapeutic delivery using ELISA and RT-PCR assays, and therapeutic outcome using qualitative scoring of fundoscopic images, scored by masked observers. A PVR grading scale, from 0-4, was used at days 14 and 28 post treatment, whereby the combination of TGF-β2 and PDGF siRNA with ultrasound resulted in the lowest mean score of 1.0 ± 0.04 and 1.5 ± 0.2 on days 14 and 28, respectively. The severity of PVR was less than nil treatment (1.8 ± 0.3 and 3.3 ± 1.5) and the combination of siRNA without ultrasound (1.1 ± 0.05 and 1.9 ± 0.6).

Retinoblastoma is a disease most commonly occurring in childhood, and whose treatment outcomes highlight the severe disproportionality of patient socioeconomic markers determining treatment success; whereby higher globe salvage is associated with better healthcare financing and accessibility, higher overall survival correlates inversely with Gini index and lower rural population numbers, and where both globe salvage and overall survival both positively correlate with education 185. Thus, improved treatment modalities which are targeted, requiring lesser amounts of high-cost chemotherapeutics, with fewer systemic side effects represent a much-needed accessible treatment modality, for which ultrasound-mediated drug delivery may suit. Two studies investigated intravitreal xenograft mouse models of retinoblastoma, relying on MBP_EXO_ either to deliver plasmid across the blood-retinal barrier and into cells, or after direct injection of plasmid and microbubbles into tumour tissue 44, 77. Both studies relied on plasmids to replace ineffective tumour protein p53 (p53) 77, or p53 and retinoblastoma (Rb94) tumour suppressor genes 44, with their functioning counterparts. Delivery of these functional genes is expected to halt cell cycle progression and induce apoptosis. To confirm delivery, both studies used RT-PCR, and Gao *et al*. (2014) also assessed protein expression using Western blot. Of these, only Gao *et al*. (2014) assessed a therapeutic outcome; quantifying the percentage of apoptotic tumour cells using TUNEL staining, whereby transfection of either p53 or Rb94 resulted in moderate cell apoptosis, and the combination of both genes significantly increased cell apoptosis, compared to non-treated and blank plasmid-treated controls (5.05 ± 0.8%, 6.43 ± 1.02%, 20.35 ± 2.14%, 0.46 ± 0.05% and 0.48 ± 0.06%, respectively).

#### 3.9.5. MRI

MRI has commonly been used in the assessment of MBP_EXO_-mediated BBB opening and molecule delivery, as it is non-invasive, aids in precision ultrasound targeting, and may report treatment success without requiring the sacrifice of treated animals, enabling longitudinal experimentation 186. In ocular delivery, MRI is most applicable in MBP_EXO_-BRB delivery and has been used to help quantify permeability improvements in two studies. Both studies relied on rodents, with one using an integrated MRI-transducer setup capable of targeting, sonication, and assessment without moving the animal 66, whilst the other relied on separate stations for targeting and sonication 15. Park *et al*. (2012), delivered a single bolus of gadopentetate dimeglumine (Gd-DTPA) via tail vein injection, which is normally unable to penetrate the BRB. They conducted serial MRI imaging of the retina, using a 3 Tesla MRI, at five-minute intervals up to thirty minutes post sonication, then repeated the injection at 3 or 3.5 h post sonication to examine BRB closure mechanics. Using this method, the researchers were able to demonstrate successful BRB opening in all sonicated mice, which returned to the pre-sonication phenotype within 3 hours 66. Comparatively, Touahri *et al*. (2020) achieved gadolinium enhancement in the retinas of half of their sonicated rats but, when repeating the experiment using tracer molecules, achieved Evans blue penetration in five of the six animals 15. Evans blue binds to albumin, with a large combined molecular weight of 67.5 kDa. As such it would be expected that the smaller gadolinium would more easily perfuse through smaller gaps in retinal vasculature. Given it is unlikely the 7 Tesla MRI used in this study failed to resolve the contrast agent, it is likely the study method may have contributed to the outcome variability. Two primary limitations should have been identified: the need to move the rat from the MRI to sonication station may have impeded appropriate targeting, and the method of ultrasound delivery lent itself toward variable delivery of sonication pressure to the eye. In particular, the researchers relied on a method devised prior in a trans-BBB study, whereby a PCD was used to measure the presence of stable cavitation using sub-harmonic emission detection, whilst up titrating the PNP of each subsequent ultrasound pulse. At the point of sub-harmonic wave detection, the pressure was reduced by 50%. Researchers successfully delivered gadolinium through the BBB in 19% of sonicated rats 187. Touahri *et al*. (2022) delivered a bolus dose of microbubbles at treatment start and applied one 10ms pulse per second (1 Hz PRF, 1% DC), similarly increasing the applied PNP until cavitation was recorded, then reduced the pressure by half for the remaining 120s of treatment, resulting in a different final pressure delivered to each rat. This method would be sound in a system where microbubbles were at steady state - four minutes into continuous microbubble infusion, for instance - however, since a bolus dose of microbubbles was given, the concentration of microbubbles would have significantly changed over the sonication duration and between rats. This may have contributed to the large variability of continuous sonication pressures used between each animal (ranging between 0.36 and 0.84 MPa). In addition, each pulse was delivered once a second, and the time taken to reach the microbubble concentration/sonication pressure combination required to achieve cavitation likely differed between each rat, meaning to the total sonication time at 50% of the cavitation threshold also differed between rats, resulting in a different cumulative 'dose' of sonication for each animal. Given, these factors, the fact the successful three gadolinium-treated rats achieved a 'weak', 'medium' and 'strong' enhancement is not surprising. Delivering the microbubbles via continuous infusion may have alleviated some of these issues, although the combination of a microbubble infusion and a constant pressure application between mice would be preferred to minimise variability.

## 4. Discussion

An extensive review of the studies involved in ultrasound-mediated drug delivery has been conducted in accordance with the ARRIVE 2.0 guidelines, SYRCLEs RoB tool and PRISMA statement. A total of 37 studies were selected according to the established inclusion criteria. The method of ultrasound-mediated drug delivery using MBP_EXO_, MBP_ENDO_ and AS have all yielded encouraging results for the delivery of various therapeutics, including plasmids, drugs, and dyes, often in combination with viral or nanoparticle vehicles. The underlying first principles of ultrasound waves and their physicochemical interactions with biological tissue and microbubbles have been synthesised, particularly in the context of their emergent mechanical and thermal indices. An overview of the animal models used, their strengths, limitations, and applicability to ocular drug delivery, and an assessment of their relevance to translation toward human trials has been completed. In addition, the safety assessments conducted in each study were characterised and, whilst generally favourable outcomes have been reported, studies often relied on structural stains only, offering limited reassurance of ultrasound safety.

The overall heterogeneity of included study protocols used for drug delivery, assessment of delivery and assessment of safety, in combination with the lack of progress toward clinical trials highlight the need for rigorous standardisation of study methods. This review of the available literature has yielded multiple key factors that may have contributed to the lack of clinical translation of ultrasound-mediated ocular drug delivery toward the clinic. These, broadly, may be divided into poor overall study reporting quality and high RoB, in combination with the limitations associated with the choice of animal models, methods of delivery efficacy assessment, and methods of safety assessment. Each of these factors is discussed and a guideline for further works is proposed (Table 11).

### 4.1. Study reporting quality and RoB

Designing animal studies to minimise or remove bias is difficult. An analysis of four bias parameters (blinding, randomisation, sample size calculation and reporting conflicts of interest) across 2,671 *in vivo* studies from 1941 to 2012 demonstrated this adversity when even recent studies published between 2008 and 2012 failed to achieve above 42% reporting in any parameter 188. Despite this challenge, researchers have a responsibility inherent in their role to ensure their research outputs are of a high standard; designing and reporting their experiments to minimise bias should be considered a minimal requirement of this role. Doing so not only assists in delivering accurate and replicable results, but may reduce wasted time and resources, and aid efficient translation of improved technologies toward clinical use.

The SYRCLEs RoB tool is designed to effectively assess the RoB in animal studies 20. As suggested by the tool developers, two independent reviewers assessed studies, with disagreements resolved through consensus-oriented discussion or via a third party. Whilst the practice of summarising and comparing the bias 'scores' of individual studies is considered overly reductive, to aid efficient discussion we have done so here, and provided the entire bias assessment of each study in Table S2. The resounding consensus for the bias assessment was damning, with all studies presenting a high or unclear risk of bias in at least six of the ten domains. Whilst it should be stated the SYRCLEs RoB tool was published in 2014, there was no trend in reducing bias over time or after its publication. In addition, the study of sources of bias in literature was well developed by the time of publication of the first included study in this review (Zderic *et al.* 2002), thus the authors of this review feel justified in the relevancy of the chosen assessment tool.

The reviewers suggest a RoB tool should be incorporated into studies moving forward, with published studies including their own assessment as a supplementary part of the paper. Particularly precarious, but easily addressed sources of bias highlighted in this review include the need to randomise animals to treatment and control groups using an approved method of randomisation, e.g., a random number table or generator. In addition, Animal characteristics after randomisation need to be reported to reassure the reader the treatment and control populations are comparable, ideally in table format. In the case of *in vivo* experimentation, animal characteristics need to include parameters relevant to ocular development, and at a minimum report animal age, weight, gender, species, sub-species, and overall health. When considering *ex vivo studies* the manner of eye collection needs to be specified, and characteristics such as eye weight, size and presentation should be noted as a minimum. Before commencing treatment, all eyes should be assessed for appropriateness for use within the study. such an assessment should be capable of identifying injury that may be caused by collection, or the application of ultrasound. These may include fluorescein staining to ensure external integrity, as well as fundoscopy to confirm the retina remains attached post collection. The time since animal death, how the eye was collected and in what manner the eye was stored (temperature and storage method) should be reported.

Whilst difficult, particularly for exploratory studies, blinding should be implemented in all stages of experimentation, including allocation of eyes to treatment or control, application of the treatment, maintenance of the animal, and assessment of the outcome measures. Blinding was typically not undertaken at any stage during the included studies, with only 5 masking outcome assessors, of which 4 also blinded safety outcome assessors 33, 35-38. Blinding should be considered mandatory, particularly for studies where successful translation toward clinic may yield a financial reward. In the case of independent research groups, this may be completed by using random number generated eye IDs, having the control application be visually synonymous and deidentified from the treatment, and by maintaining random animal IDs until after outcome assessment completion. In the case of larger research groups, applying an isolated approach whereby the masked outcome assessors differ from the masked investigators and animal caregivers would be ideal.

Reporting quality of the included studies was also problematic, with 5, 24 and 8 studies scoring an 'insufficient', 'poor' and 'average' rating, respectively. Whilst there may be consideration for obfuscating experimental design to maintain intellectual property in this translatable field, most studies declined to report basic study details. Studies failed to include the original sample size of animals, any attrition, and the causes thereof. No study justified the chosen sample size or provided an assessment of the number of participants required to provide enough power to reassure the significance of the results. Publishing a study protocol prior to experimentation would go a long way toward addressing the concerns raised due to these missing details and would improve the transparency of published study designs. Suffice to say, just the act of using an appropriate quality of reporting assessment tool, such as the ARRIVE 2.0 guidelines, during study design and writing stages, and citing the guide used, would wholly benefit future published material. Incorporating these systemic changes into study designs in this field would promote efficient translation of works into clinic.

### 4.2. Mechanisms of ultrasound-mediated drug delivery

A key factor influencing the scope of a given study was the mechanism by which ultrasound was used to facilitate drug delivery. Studies often utilised a combination of MBP_EXO_, MBP_ENDO_ and/or AS to improve delivery, however the mechanisms proposed by studies to improve delivery were mostly suggested theoretically rather than through experimentation. of the 37 included studies, 30 refer to bubble cavitation, however only five directly measured cavitation activity of endogenous microbubbles 61, 62, 72, 73. Four of these studies successfully highlighted the benefits of optimising ultrasound parameters toward producing stable cavitation to improve trans-topical drug delivery, as inertial cavitation further reduced delivery and increased the severity and incidence of adverse events. Comparatively, the single study assessing cavitation as part of MBP_EXO_-mediated trans-BRB drug delivery applied a method to first detect stable cavitation, then reduce the pressure by 50% thereafter; a method used with mild success previously in a BBB-opening study 187, but for which held severe risk of heterogenous ultrasound delivery in this study due to the chosen method of microbubble administration 15. No other studies claiming to utilise MBP_EXO_ directly measured cavitation activity, although most claimed inertial cavitation to play the primary role in improved efficacy of delivery. All three mechanisms of ultrasound delivery rely on cavitation activity in some fashion; thus, the lack of passive cavitation detection detracts from the dependability of discussions around mechanisms of delivery presented by authors. As such, it would be ideal to either incorporate PCDs into experimental designs, or soundly justify the lack thereof. Such a justification would be challenging, given the cavitation characteristics of both endogenous and exogenous microbubbles rely heavily on their surrounding environment, applied sonication parameters, and their shell characteristics, size, and concentration. The often-occurring claim of “we may assume stable/inertial cavitation occurred in our experiments as these ultrasound parameters generated cavitation in other studies previously” made by researchers is thus inherently flawed due to the inherent complexity associated with factors influencing the likelihood of cavitation; cavitation should be confirmed experimentally within the published study.

### 4.3. Factors influencing efficiency of delivery

Most of the included studies relied on single-element planar transducers to deliver microbubbles. These primarily have the advantage of being cheap, with simple targeting characteristics. When considering the reliability of pressure characteristics of the delivered wave, placing the target of ultrasound at the intersection between the Fresnel and Fraunhofer zones may minimise variability. Comparatively, focused transducers have the benefit of tuning the Fresnel zone length based on the degree of curvature of the chosen transducer, at the disadvantage of increased cost. In any case, five included studies failed to appropriately describe the transducers used, severely limiting their ability to be reliably replicated. Given the context of application in these studies, the reviewers were able to surmise these were planar transducers, however this highlights the need for some minimum detail of the transducers used, including transducer width, type, manufacturer and intended application (for instance whether they are manufactured for immersion or for gel coupling). Justification of the choice and characteristics of the chosen transducer used would be a beneficial inclusion in future studies.

Of particular importance to translation and replicability is the accurate and comprehensive reporting of sonication parameters. One study failed to describe the frequency of sonication applied, completely removing any capacity for comparison with other studies or translation of its application toward clinic 76. 29 studies only reported the magnitude of ultrasound delivered in terms of power, 2 studies only reported PNP, whilst the remaining 6 studies reported both. Since the US-FDA requires both an ultrasound device's TI and MI to be reported during sonication, and these indices require power and PNP for their calculation, respectively, we strongly advise a more comprehensive reporting of both parameters for future studies 63. Methods of measuring these parameters have been discussed in this review, and an appropriate method should be described within the published study. Particularly relevant to reporting the power applied, the method of power measurement clarifies which of the six intensity terms are being used (*I*_SPTP_, *I*_SPTA_, *I*_SPPA_, *I*_SATP_, *I*_SATA_ or *I*_SAPA_). It should be noted the US-FDA requires power output to be reported as I_SPTA.3_, thus studies aiming for translation of their technology should at least measure and report this aspect. However, as I_SATA_ has been shown to be the best predictor of tissue heating 111, researchers may find benefit in measuring and reporting both parameters.

When considering PNP measurements, the primary justification for measurement relies on the US-FDA maximum MI standards for ophthalmic ultrasound 63. This requires the reporting of both applied frequency and PNP, for which the method of measurement should be described. In addition, a statement clarifying whether the reported power and PNP measurements were derated, and a justification for the degree of derating would also be ideal. Such a justification may quote the US-FDA standard attenuation of 0.3 dB cm^-1^ MHz^-1^ or may be specific to the delivered beam after it passes through individual ocular structures, for which the attenuation has been supplied in Table 5 of this review.

Included studies have also shown the PD of delivered ultrasound to impact the efficiency of delivery, however 5 of the included studies either did not report PD or failed to report relevant parameters required to calculate the PD, making study replication impossible. Therefore, a full characterisation of the delivered ultrasound should be reported, including PRF, DC, SD, and PD. If the study utilises continuously applied ultrasound, then this should be clearly stated as well.

The delivery of microbubbles in AS or MBP_EXO_ applications in the included studies was mostly well reported when delivering commercial bubbles. Comparatively, studies utilising in-house microbubbles did not consistently report basic bubble aspects, such as method of manufacture, bubble concentration, size, gas and/or shell composition 37, 65, 75, 77. These parameters significantly impact the degree of acoustic energy translated into mechanical within the surrounding tissue, and thus their reporting is imperative. Comparatively, whilst determinations of shell stiffness and friction are relevant and useful in the context of ultrasound-mediated drug delivery, their lack of characterisation is unlikely to impact the replicability of the study (but are useful for modelling microbubble behaviour). The method of microbubble administration should be justified in line with the intended mechanism by which their addition improves drug delivery. For intravenously administered microbubbles, particularly when applying ultrasound to multiple areas of the retina, a well-reasoned method of delivery is paramount. An IV bolus of microbubbles will decay rapidly after administration; therefore, cavitation activity is unlikely to be stable during longer sonication periods or repeated sonications. Addressing this through repeated boluses harbours the significant risk of further deranged cavitation activity in recurrent sonications, as was highlighted when considering the study by Park *et al*. (2012) 66. Delivering microbubbles using an infusion and allowing time for the microbubbles to reach steady state within the body addresses this concern. Using a PCD throughout the duration of sonication allows for direct measurement and comparison of cavitation activity both within and across multiple treatment subjects.

### 4.4. Applications in animal models

The use of animal models in ocular drug delivery is characterised by a large skew toward animals with lower barriers to entry for research, but larger barriers to direct translation toward human use. The method of ultrasound delivery was also disproportionately represented within certain animal groups; small rodents were primarily used by MBP_EXO_-related studies assessing improved transfection of genetic material, requiring the maintenance of live subjects. Comparatively, MBP_ENDO_ studies mostly relied on the larger eyes of the readily rabbits. Only one of the included studies justified their choice of animal 64, and only two acknowledged the differences between rodent and human eyes may limit relevancy of the results 45, 75.

The severe lack of progression in follow-up studies toward eyes more representative of humans highlights both a potential factor contributing to the lack of successful clinical translation, and the barriers to entry associated with acquiring and maintaining larger mammals. All the studies utilising large mammal material did so posthumously, thus avoiding challenges associated with *in vivo* animal maintenance and the more rigorous ethics approvals. Unfortunately, this also precluded longitudinal assessments of safety post sonication, and is not suitable for gene therapy experiments. The argument may be made that these are primarily exploratory or proof of concept studies, thus the choice of animal model is appropriate; however, all included studies were to some extent successful and claimed their results contributed toward translation, but are consistently yet to follow-up using more rigorous models. Other routes of drug delivery, such as transdermal or oral delivery are often able to rely on rodent models due to heavily conserved physiologies 189, 190. In the case of ocular drug delivery, the intersection between ultrasound behaviour and globe anatomy severely limits the translatability of results. For instance, direct injection of genetic material into the retina, followed by sonication is wholly more approachable in the rodent compared to the human. Additionally, the comparative thinness of most parts of the sclera in rabbits compared to humans overestimates the magnitude of trans-topical drug delivery. Future studies need to transition established ultrasound-mediated drug delivery technologies toward *in vivo* application in large mammal models. Given the understandably high barriers to entry associated with the use on non-human primates, the next most relatable animals would be cows, pigs, or sheep. In addition, given the end goal of drug delivery is to treat or prevent the progression of disease; to efficiently facilitate clinical translation, such a study would be ideally aimed to determine the improved treatment efficacy in a large mammal model of ocular disease. Significant, longitudinal assessment of safety would need to be a key aspect of such a study, to justify a change in current medical practice.

### 4.5. Methods of safety assessment

Studies investigating ultrasound-mediated ocular drug delivery often failed to complete comprehensive safety assessments, yet almost all demonstrated the potential damage caused by excessive sonication of this delicate organ in basic structural stains. Most studies utilised transducers either repurposed from their usual applications, such as sonoporation or non-destructive testing, or created transducers in-house. In either case, the transducers and parameters used usually have not demonstrated their safety in ophthalmic human use. Whilst regulating agencies such as the US-FDA have prescribed guiding upper limits associated with the intensity of ultrasound applied, namely MI and TI, these indices are generally reductive, and fail to incorporate duration of treatment into their calculation. In addition, the derated calculations built into the indices are not representative of ocular tissue attenuation. Given these factors, determining the safety of experimental ultrasound in a comprehensive manner should be a priority to researchers, and constitutes a major barrier to translation.

Despite the limitations associated with the prescribed mechanical and thermal safety limits, they allow a ballpark minimum assessment of safety, and a limited method by which studies may be compared. 29 of the 37 studies either did not investigate, or did not report, the PNP delivered into tissues, making a calculation of MI impossible. In addition, 23 of the included studies did not assess changes in temperature during experimentation. Increased globe temperature can damage the eye and overheating of the lens has resulted in cataract formation in the past 104, 164. Temperature assessment should be considered a basic requirement of sonication studies in ocular tissue, and future studies need to incorporate high accuracy thermometry into their design.

The eye is a complicated organ, with some capacity for regeneration, particularly in the corneal and conjunctival epithelium. Other tissues such as the retina and RPE typically rely on complicated repair pathways which may progress toward permanent scarring or complete repair depending on the nature and duration of insult. The included studies mostly relied on single or delayed assessments of tissue healing, but not both. In addition, there was a negative trend in reported adverse effects reported by separate studies which correlated with the length of time between sonication and safety assessment. thus, it is possible the delayed safety assessments in some studies investigated sonicated tissue health after the diminution of damage due to fast healing. Since repeated injury increases the risk of scaring, and these novel therapies are positioned toward repeated application, there is a risk of chronic injury being masked in these studies. As such, safety assessments need to be comprehensive in terms of both the methods of assessment used, and the assessment timing, with an example assessment timeline being t = 0, 60 min, 24 hr and 7-10 days to provide a more complete picture of tissue damage.

Many of the MBP_ENDO_-based studies assessed trans-topical delivery using dissected corneal tissue in a Franz diffusion cell, curtailing any possibility of assessing the health of retinal tissues originally in the beam path. The remaining *in vivo* trans-topical studies only considered the health of sonicated scleral or corneal tissue, despite the consideration that ultrasound will impact all tissues within the beam cross-section until the wave has been fully reflected or attenuated. Studies need to expand their consideration of tissue health to take this into account. In addition, larger transducers will have a Fresnel distance extending beyond the globe and into the cranium. Since bone has a greater attenuation coefficient compared to soft tissue, this may result in significantly greater warming than expected. Directly behind the eye are three key openings for neuronal pathing: the superior orbital fissure through which cranial nerves (CN) III, IV, V-1 and VI travel, the optic canal which holds the optic nerve (CN II), and the inferior orbital fissure containing the V-2 nerve 191. Damage to nerves travelling through these fissures, be it by thermal or mechanical effects, is a concern not considered by any of the included papers. Thus, future studies would greatly benefit by assessing and discussing the potential risks to these tissues due to treatment.

The choice of safety assessment included in most studies primarily relied on basic structural stains, such as H&E, toluidine blue or Richardson's stain. Four studies assessed structure using SEM, TEM or SHGI, and 11 studies did not assess tissue morphology at all. Whilst there is a barrier to entry for sub-micron imaging assessments, the use of H&E should be considered mandatory for translational studies using ultrasound, due to the clear thermal and mechanical impacts applied to tissue. In addition, whilst perhaps more challenging in *ex vivo* models, the use of TUNEL staining as a secondary measure of damage would be ideal, particularly if tracked over multiple time points as suggested above. Assessment of protein changes, upregulation of inflammatory markers, or the infiltration of blood-borne entities, as completed by one study, allows for a comprehensive assessment of the impacts of the delivered treatment on tissue health. Whilst immunohistochemistry does impart challenges to experimental design and tissue processing, for studies aiming to have their technology progress toward human use, the inclusion of more comprehensive and well-reasoned assessments of safety will be critical.

## 5. Conclusions

Efficient treatment of ocular disease by conventional means is challenged by the presence of static and dynamic barriers, limiting efficient ingress of topically, orally- and systemically- administered drugs toward therapeutic targets. Using ultrasound as an adjunct tool in ocular drug delivery has been proposed as non-invasive method to overcome these barriers, with applications in improving systemic, topical, and directly injected drug delivery. Whilst studies published to date all report significantly improved ocular delivery using ultrasound, the extensive heterogeneity in the methods and parameters used, in combination with the decidedly poor reporting quality and high RoB severely impedes the progress toward clinical trials. Concerted effort to address these limitations in study design and reporting, and a demonstration of safety and improved treatment efficacy particularly in longitudinal disease models using appropriate animals is warranted before translation can be considered. Considering these shortcomings, we have produced a set of guidelines with a particular focus on addressing the problem areas identified in this review, with the aim of facilitating efficient translation of this technology to clinic.

## Mathematical symbols


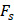
: the driving force of acoustic streaming per unit volume; 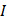: the acoustic intensity emitted by the transducer; 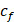: the speed of sound in the fluid medium; α: attenuation coefficient of the medium; 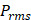: the root-mean-square pressure; 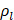: the density of the liquid: 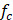: the centre frequency of ultrasound; 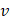: the kinematic viscosity coefficient; 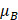: the bulk viscosity coefficient; 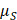: the shear viscosity coefficient: 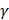: the ratio of specific heats; 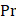: the Prandtl number describing the ratio of kinematic viscosity to thermal diffusivity: 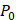: the acoustic pressure amplitude; 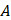: the vibration amplitude of the ultrasound transducer; 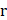: the radius of the ultrasound beam; 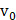: acoustic streaming velocity; 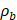: the density of the gas core of the microbubble; 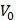: the initial bubble volume; 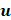: the microbubble velocity; 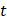: time; 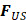: the ultrasound radiation force; 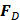: the drag force; 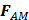: the “added mass” force; 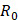: the bubble radius at equilibrium; 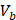: the bubble volume; 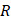: the bubble radius; 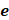: the unit vector in the direction of the wave propagation; 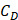: the drag coefficient of a sphere; 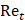: the translational Reynolds number; 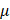: the liquid viscosity; 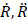: the time derivatives of 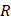; 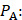 the atmospheric pressure; 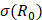: the initial surface tension of the bubble shell; 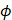: the polytropic component of the gas core; 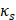: the dilatational surface viscosity; 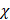: is the surface elasticity of the bubble; 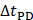: pulse duration (time); 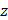: bubble translation 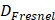: Fresnel zone length; 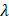: wavelength; 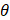: the Fraunhofer divergence angle in degrees; 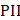: pulse intensity integral; 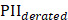: peak derated pulse intensity integral; 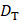: the distance from the transducer; 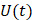: hydrophone voltage; 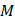: the hydrophone sensitivity at the centre frequency; 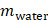: the mass of water in grams; 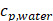: the specific heat capacity of water; 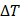: change in temperature; 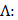
the cross-sectional area of the transducer probe; 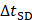: sonication duration (time) 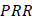: the pulse repetition rate; 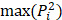: the maximum pressure-square integral; 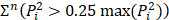: the sum of all data within the matrix measured by the hydrophone with a value greater than 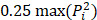; 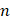: the number of data points greater than 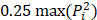 times the size of the grid, or the effective radiation area; 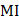: mechanical index; 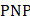: peak negative pressure; 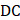: duty cycle; 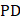: pulse duration; 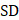: sonication duration; 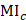: the cumulative mechanical index; TI: thermal index; 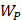: relevant acoustic power at the depth of interest; 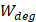: the acoustic power required to raise the temperature of the tissue by 1°C; 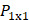: the derated bounded-square power; 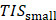: the soft tissue thermal index of a non-scanning transducer of aperture < 1 cm^2^; 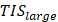: the soft tissue thermal index of a non-scanning transducer of aperture > 1 cm^2^; 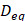: the equivalent aperture diameter of an ultrasound transducer of aperture > 1 cm^2^; 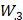: the output power derated by 0.3 dB cm^-1^ MHz^-1^; 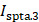: the spatial peak time averaged output power derated by 0.3 dB cm^-1^ MHz^-1^; 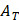: the transducer area.

## Supplementary Material

Supplementary tables S1, S3, S4.

Supplementary table S2: data characterisation.

## Figures and Tables

**Table 1:** Key elements of the research question expressed in terms of Population, Concept and Context.

Population	Eye diseases, the eye, and ocular structures, including the conjunctiva, sclera, cornea, retina, retinal pigment epithelium, anterior and posterior chambers, anterior and posterior segments.
Concept	Drug delivery, dosage form design, therapeutics, and ultrasound.
Context	*In vivo or ex vivo* studies, published between 2002-2022.

**Table 2:** Intra-study examination of the proportion of answered ARRIVE 2.0 questions compared to the total number of applicable questions expressed as a coefficient.

Ref	Year	Animal model	Target	Coefficient	Quality of study reporting
69	2002	Rabbit	Transcorneal to anterior segment	0.36	Insufficient
62	2004	Rabbit	Transcorneal to anterior segment	0.39	Insufficient
60	2004	Rabbit	Transcorneal to anterior segment	0.53	Poor
36	2006	Rabbit	Intrascleral	0.63	Average
37	2007	Rat	Intraconjunctival	0.56	Poor
64	2008	Bovine	Vitreal-retinal uptake	0.42	Poor
82	2009	Rat	Subretinal	0.46	Poor
75	2009	Rat	Vitreal-retinal uptake and blood-retinal barrier	0.39	Insufficient
38	2009	Rat	Vitreal-retinal uptake and subretinal	0.51	Average
77	2010	Mouse	Intravitreal and vitreal-retinal uptake	0.34	Insufficient
76	2010	Rat	Blood-retinal barrier	0.46	Poor
33	2010	Rat	Vitreal-retinal uptake	0.59	Poor
61	2010	Rabbit	Intrascleral	0.53	Poor
28	2011	Rat	Intra-ciliary	0.61	Average
154	2011	Rat	Vitreal-retinal uptake	0.61	Average
66	2012	Rat	Blood-retinal barrier	0.61	Average
65	2012	Rabbit	Vitreal-retinal uptake	0.56	Poor
34	2012	Rat	Vitreal-retinal uptake	0.56	Poor
71	2013	Rabbit	Intrascleral	0.58	Poor
70	2013	Rabbit	Transcorneal to anterior segment	0.44	Poor
39	2013	Rabbit	Transscleral to posterior segment	0.59	Poor
44	2014	Mice	Blood-retinal barrier	0.56	Poor
16	2014	Rabbit	Transcorneal to anterior segment	0.66	Average
74	2014	Rabbit	Transscleral to posterior segment	0.47	Poor
49	2015	Pig	Intrascleral	0.33	Insufficient
83	2015	Rat	Subretinal	0.54	Poor
35	2016	Rat	Subretinal	0.59	Poor
73	2016	Rabbit	Intraconjunctival	0.43	Poor
46	2016	Rabbit	Vitreal-retinal uptake	0.46	Poor
17	2017	Rat	Vitreal-retinal uptake	0.61	Average
72	2017	Rabbit	Intrascleral	0.50	Poor
40	2017	Pig & Cow	Intravitreal and vitreal-retinal uptake	0.56	Poor
41	2018	Rabbit	Intrascleral and transscleral to posterior segment	0.64	Average
45	2019	Mouse	Intraconjunctival	0.54	Poor
42	2019	Pig & Cow	Intravitreal and vitreal-retinal uptake	0.53	Poor
15	2020	Rat & Mouse	Blood-retinal barrier	0.49	Poor
43	2021	Pig & Rabbit	Transcorneal to anterior segment	0.54	Poor

Quality of study reporting defined by the following coefficient ranges: Insufficient ≤0.4, Poor 0.41-0.6, Average 0.61-0.8, and Excellent ≥ 0.81.

**Table 3:** Summary of the methods used by included studies to facilitate ultrasound-mediated ocular drug delivery.

Ref	Method of delivery			Delivery pathway				Animal model					Delivery assessment		Delivered compound					
	MBP_EXO_	AS	MBP_ENDO_	Trans-topical	Blood-retinal barrier	Vitreal/ Vitreoretinal	Direct injection into target tissue	Small rodent	Rabbit	Large Mammal		Coadministered microbubbles?	Cavitation assessed using PCD (W/cm^2^)	Streaming assessed experimentally	Virus	Plasmid	siRNA	Small Molecule	Fluorophore/ contrast agent	Topical co-incubation time (min)
69	-	✓	✓	✓	-	-	-	-	✓	-		-	-	Yes - contributed to delivery	-	-	-	✓	-	60
62	-	✓	✓	✓	-	-	-	-	✓	-		-	Stable @ 0.19-0.56Inertial @ 0.34-0.56	Yes - contributed to delivery	-	-	-	-	✓	5*
60	-	✓	✓	✓	-	-	-	-	✓	-		-	-	-	-	-	-	-	✓	60
36	✓	-	-	-	-	-	✓	-	✓	-		✓	-	-	-	✓	-	-	-	-
37	✓	-	-	-	-	-	✓	✓	-	-		✓	-	-	-	✓	-	-	-	-
64	-	✓	✓	-	-	✓	-	-	-	✓		-	-	-	-	✓	-	-	✓	-
82	✓	-	-	-	-	-	✓	✓	-	-		✓	-	-	✓	-	-	-	-	-
75	✓	✓	-	-	✓	✓	✓	✓	-	-		✓	-	-	-	✓	-	-	-	-
38	✓	-	-	-	-	-	✓	✓	-	-		✓	-	-	✓	-	-	-	-	-
77	✓	-	-	-	✓	-	✓	✓	-	-		✓	-	-	-	✓	-	-	-	-
76	-	✓	✓	-	✓	✓	-	✓	-	-		✓	-	-	-	-	-	✓	-	-
33	✓	-	-	-	-	✓	-	✓	-	-		✓	-	-	✓	-	-	-	-	-
61	-	✓	✓	✓	-	-	-	-	✓	-		-	Stable @ all frequencies	Yes - contributed to delivery	-	-	-	-	✓	5, 15, 30, 60
28	✓	-	-	-	-	-	✓	✓	-	-		✓	-	-	-	✓	-	-	-	-
154	✓	-	-	-	-	✓	-	✓	-	-		✓	-	-	-	-	✓	-	✓	-
66	✓	-	-	-	✓	-	-	✓	-	-		✓	-	-	-	-	-	-	✓	-
65	✓	✓	-	-	-	✓	-	-	✓	-		✓	-	-	-	✓	-	-	-	-
34	✓	✓	-	-	-	✓	-	✓	-	-		✓	-	-	✓	-	-	-	-	-
71	-	✓	✓	✓	-	-	-	-	✓	-		-	-	-	-	-	-	-	✓	45
70	-	✓	✓	✓	-	-	-	-	✓	-		-	-	-	-	-	-	✓	✓	60
39	-	✓	✓	✓	-	-	-	-	✓	-		-	-	-	-	-	-	-	✓	6.5, 11.5, 16.5
44	✓	-	-	-	✓	-	-	✓	-	-		✓	-	-	-	✓	-	-	-	-
16	-	✓	✓	✓	-	-	-	-	✓	-		-	-	-	-	-	-	✓	-	5*
74	-	✓	✓	✓	-	-	-	-	✓	-		-	-	-	-	-	-	-	✓	10, 20, 30, 40
49	-	✓	✓	✓	-	-	-	-	-	✓		-	-	-	-	-	-	-	✓	<1**
83	✓	✓	-	-	-	✓	-	✓	-	-		✓	-	-	-	✓	-	-	-	-
35	✓	-	-	-	-	-	✓	✓	-	-		✓	-	-	-	✓	-	-	-	-
73	-	✓	✓	✓	-	-	-	-	✓	-		-	Stable @ 0.002 to 0.05Inertial at 0.38 to 1.8	-	-	-	-	-	✓	15
46	✓	✓	-	-	-	✓	-	-	✓	-		✓	-	-	-	-	-	✓	-	-
17	✓	✓	-	-	-	✓	-	✓	-	-		✓	-	-	-	-	✓	-	✓	-
72	-	✓	✓	✓	-	-	-	-	✓	-		-	Stable @ all frequencies	Yes - contributed to delivery^	-	-	-	-	✓	<1, 15
40	-	✓	✓	-	-	✓	-	-	-	✓		-	-	-	-	-	-	-	✓	-
41	-	✓	✓	✓	-	-	-	-	✓	-		-	-	-	-	-	-	-	✓	60
45	-	-	✓	✓	-	-	-	✓	-	-		-	-	-	-	-	-	✓	✓	10***
42	-	✓	-	-	-	✓	-	-	-	✓		✓	-	Yes - contributed to delivery	-	-	-	-	✓	-
15	✓	-	-	-	✓	-	-	✓	-	-		✓	Depended on mouse^^	-	✓	-	-	-	✓	-
43	-	✓	✓	✓	-	-	-	-	✓	✓		-	-	-	-	-	-	✓	✓	30, 120

*Eye bath containing drug was removed after 5-minute sonication, however the eye was not rinsed after. **HIFU transducer. ***15 min sonication followed by drug. ^small molecules only. ^^Sonication pressures reported were not clearly defined.

**Table 4:** Summary of the effect of altering ultrasound parameters on drug delivery efficacy and adverse effects on tissue.

Ultrasound parameter	Method of ultrasound	Range of parameters studied and (majority of parameters studied)	Parameters directly compared	Effect of changing a given parameter on safety	Effect of changing a given parameter on delivery
Frequency	MBP_EXO_	0.3-3 MHz (1 MHz)	---------------------------------------------Not directly compared---------------------------------------------		
	AS and MBP_ENDO_	0.02-3 MHz (0.88-1 MHz)	0.04, 0.5, 1 and 3 MHz [72]0.4, 0.5, 0.8, 1.0 MHz [70]0.4 and 0.6 MHz [16]1 and 3 MHz [61]1 and 3.3 MHz 74	Reducing frequency increases epithelial damage at higher pressures and durations 16, 70, 74.	Transscleral delivery: Penetration depth through sclera increased with decreasing frequency 16, 61, 70, 72, 74.
Power	MBP_EXO_	0.15-3 W/cm^2^(0.5 or 2 W/cm^2^)	0.5, 1, 2 and 2.5 W/cm^2^ [33]1, 1.5 and 2 W/cm^2^ 36	Increasing power significantly increases vascular and epithelial damage, and results in inflammatory infiltrates, particularly when using continuous ultrasound 33, 36.	Increasing power does not appear to improve transfection efficacy 33, 36.
	AS and MBP_ENDO_	0.002-2.5 W/cm^2^ (0.05, 0.5 and 1 W/cm^2^) 0.05 or 105 W/cm^2^ using HIFU	0.002, 0.01, 0.05, 0.12, 0.38 and 1.8 W/cm^2^ [73]0.07, 0.31 and 1.22 W/cm^2^ [43]0.19, 0.34 and 0.56 W/cm^2^ [60, 62]0.3, 0.5, 0.8 and 1.0 W/cm^2^ [70]0.5 and 1 W/cm^2^ [64]1, 1.3 and 1.5 W/cm^2^ 45	Increasing power increases scleral temperature and the presence of corneal pitting and debridement, however structural changes appear rapidly reversable 73.	Scleral penetration is maximised when inducing stable cavitation in the topical media, but higher intensities, where inertial cavitation predominates appears to decrease penetration 43, 60, 62, 64, 73.Increasing power improves permeability at lower frequencies 70. When increasing power improves penetration, molecule retention in the sclera is prolonged 45.
Peak Negative Pressure	MBP_EXO_	Not measured using planar transducers,0.36-1.1MPa using HIFU	0.81, 0.88 and 1.1 MPa 66	Increasing PNP increases vascular damage 66.	Increasing PNP improves BRB permeation 66
	AS and MBP_ENDO_	0.041-0.165MPa (0.08-0.13MPa)15.7Mpa using HIFU	0.08, 0.1 and 0.13 MPa [60, 62]0.041, 0.082 and 0.165 MPa 43	Results as for power assessments 43, 60, 62.	Results as for power assessments 43, 60, 62
Sonication duration	MBP_EXO_	4-300 s(60-300 s)60 or 120 s using HIFU	---------------------------------------------Not directly compared---------------------------------------------		
	AS and MBP_ENDO_	30-3600 s(300-600 s)30 or 100 s using HIFU	30 and 120 s [64]60 and 240 s 42 600, 1800 and 3600 s [69]600 and 1800 s 43	Longer sonication durations resulted in clouding of the lens, tissue dehydration and retinal delamination 42.	Increasing the sonication duration increased the vitreoretinal delivery of small (53nm), and medium (131nm) but not larger (218nm) nanospheres [64]Increasing sonication duration further improved transscleral and corneoscleral delivery 43, 69.
Pulse repetition frequency	MBP_EXO_	1 - 100 Hz or continuous(100 Hz)	---------------------------------------------Not directly compared---------------------------------------------		
	AS and MBP_ENDO_	0.1428-1 Hz or continuous(continuous)			
Duty cycle	MBP_EXO_	1 - 50% or continuous(50%)	---------------------------------------------Not directly compared---------------------------------------------		
	AS and MBP_ENDO_	14.3% - 50% or continuous(50% or continuous)	50% and continuous 64	-	Decreasing the duty cycle from continuous to 50% doubled the delivery of smaller (53 nm), but not larger (131 nm) nanospheres into retinal pigment epithelial cells through the neural retina 64.
Pulse duration	MBP_EXO_	5-50 ms or continuous(5 or 10 ms)	---------------------------------------------Not directly compared---------------------------------------------		
	AS and MBP_ENDO_	5-1000ms or continuous (continuous)10-100ms using HIFU	10, 20, 50 and 100 ms using HIFU 49	Increasing the pulse duration caused erosion of the corneal surface 49.	Increasing pulse duration increased transscleral penetration distance 49.

**Table 5:** Summary of mean thermal and acoustic properties of human ocular tissues.

Structure	Acoustic impedance, (MRayl)	Density, (g/cm^3^)	Attenuation, (dB cm^-1^ MHz^-1^)	Speed of sound, (m/s)	Thermal conductivity (W/mK)	Specific Heat (J/kgK)	Ref.*
Vitreous Humour	1.54±0.04	1.01	0.01	1532	0.60	3999	24, 192
Retina	1.57±0.01	0.97±0.03	3.56±1.21	1618±58	0.57	3680	24, 193
Sclera	1.67±0.06	1.03±0.04	11.7±2.99	1618±46	0.58	4178	24, 193
Corneal epithelium	1.60	1.01	0.78	1586	0.58	4178	24, 194
Corneal stroma	1.63±0.05	0.98	3.9±2.8	1651±37			
Lens	1.73±0.03	1.06	1.38	1637	0.4	3000	24, 169
Ciliary muscle	1.58±0.02	0.98±0.03	6.05±1.56	1610±52	Unknown		193
Trabecular meshwork	1.64±0.05	1.02±0.02	10.53±3.01	1600±52	Unknown		193
Optic nerve	1.57±0.02	0.98±0.03	3.99±1.27	1611±57	0.53	3750	24, 193
Choroid	1.59±0.02	0.99±0.04	6.95±2.08	1612±63	0.6	3840	24, 193
Water	1.48	1.00	0.0025	1480	0.6	4182	195

*Where referenced studies disagree on thermal and acoustic ocular properties, only the most recently published results have been shown.

**Table 6:** overview of commercial microbubble characteristics used in ultrasound-mediated ocular drug delivery applications.

Ref	Brand	Animal model			Intended destination		Route of administration		Microbubble parameters							
		Small rodents	Rabbits	Large Mammals	BRB	V/VR	Local	Systemic	Dose (µl)	Concentration (bubbles/ml)	Mean Diameter (µm)	Gas	Shell composition	Bubble superstructure	Shell stiffness N/m	Shell friction kg/s
46	SonoVue	-	✓	-	-	✓	✓	-	I_vit_: 100	2-5x10^8^	2.5 125, 196	SF^6^ 196	DPPG*, DSPC**	Phospholipid monolayer	0.46 197	7.2x10^-9^ 197
82		✓	-	-	-	-	✓	-	SR: 4							
38		✓	-	-	-	-	✓	-	SR: 1							
154		✓	-	-	-	✓	✓	-	I_vit_: 2							
34		✓	-	-	-	✓	✓	-	I_vit_: 2							
83		✓	-	-	-	✓	✓	-	SR: 1							
35		✓	-	-	-	-	✓	-	SR: 1.6							
17		✓	-	-	-	✓	✓	-	I_vit_: 3							
66	Definity	✓	-	-	✓	-	-	✓	IV: 20/kg	1.2x10^10^	1.1-1.3 125	C^3^F^8^	DPPA***, DPPC^, MPEG5000 DPPE^^	Phospholipid monolayer	0.7 197	2.8x10^-9^ 197
15		✓	-	-	✓	-	-	✓	IV: 200/kg	1.2x10^9^						
28	Artison	✓	-	-	-	-	✓	-	I_cil_: 1.5	1.3x10^9^	2.4 198	-------------Unknown, company has dissolved-------------				
36	Optison	-	✓	-	-	-	✓	-	I_cor_: 2	5-8x10^8^	3.55 196	C^3^F^8^ 196	Albumin	Gas encapsulating microsphere	0.9 199	8.15x10^-6^ 199
37		✓	-	-	-	-	✓	-	I_con_: 40							
37	In-house	✓	-	-	-	-	✓	-	I_con_: 40	Unknown	<0.2	C^3^F^8^	DSPC**, DSPE-PEG (2k)-OMe^^^	Echogenic liposome	-----Unknown-----	
42		-	-	✓	-	✓	✓	-	I_vit_: 100	1.9x10^10^	0.2	C^3^F^8^	DPPC^, DSPE-PEG (2k)-OMe^^^,		0.11 134	0.31 134
33		✓	-	-	-	✓	✓	-	I_vit_: 2.5	8.7x10^9^	2.8	C^3^F^8^	DSPC**, DSPEᵔ, DSPAᵔᵔ		-----Unknown-----	
44		✓	-	-	✓	-	-	✓	IV: 500	1.8x10^9^	3-5	C^3^F^8^	DSPC**, DPPEᵔᵔᵔ, Glucose		-----Unknown-----	
65		-	✓	-	-	✓	✓	-	I_vit_: 50	-----Unknown----		C^3^F^8^	DSPC**, DSPE-PEG (2k)-OMe^^^		-----Unknown-----	
75		✓	-	-	✓	✓	✓	✓	I_vit_: 2RB: 30IV: 125,	8.3x10^8^	2.0-3.5	-----------------------Unknown--------------------------------				
77		✓	-	-	✓	-	✓	✓	IV: 200 I_vit_: 200^Ω^	1.2x10^9^	4-6	------------------------Unknown-------------------------------				

I_cor_ intracorneal, SR subretinal, I_vit_ Intravitreal, Ic_il_ intraciliary, I_con_: Intraconjunctival, RB retrobulbar, IV intravenous, ^Ω^Intravitreal injection of 200 µL into mouse eye was possible due to retinoblastoma xenograft model significantly increasing ocular volume, *1,2-Dipalmitoyl-*sn*-glycero-3-phosphoglycerol, **1,2-Distearoyl-*sn*-glycero-3-phosphocholine, ***1,2-Dipalmitoyl-*sn*-glycero-3-phosphate, ^1,2-Dipalmitoyl-*sn*-glycero-3-phosphocholine, ^^N-(Carbonyl-methoxypolyethylenglycol 5000)-1,2-dipalmitoyl-sn-glycero-3-phosphoethanolamine, ^^^1,2-distearoyl-sn-glycero-3-phosphatidyl-ethanolamine-(methoxy-polyethleneglycol 2000), *ᵔ*1,2-distearoyl-sn-glycero-3-phosphoethanolamine, *ᵔᵔ*1,2-distearoyl-sn-glycero-phosphoacid, *ᵔᵔᵔ*1,2-Dipalmitoyl-sn-Glycero-3-Phosphatidylethanolamine

**Table 7:** Comparison of ocular tissue thickness and vitreal volume of distinct species at various landmarks.

	A. central corneal, µm	B. Peripheral corneal, µm	C. Sclera - limbus, µm	D. Sclera - equator, µm	E. Sclera - para-optic, µm	F. Vitreal volume, mm^3^	Ref
Human	535±20	657±71	500	420	860	4,650±426 (♀)4,969±465 (♂)	56, 140, 141
Mouse	137±14	90.55±1.9	Unknown	41.0±6.3	61.1±8.0	5.3	140, 142
Rabbit	370	450	500	200*-250^	180	1,150-1,500	143
Porcine	666±68	714±54	910±170-430±130**^y^**	730±140	730±140	3,300	143-145
Bovine	850	750	920±60	646±43	1100	14,600±700º	143, 144

*Inferior sclera, ^superior sclera, **^y^**porcine sclera reduces in thickness to a minimum 6mm posterior to the limbus, ºmeasured experimentally by the review authors, raw data available in Table S4.

**Table 8:** Safety assessments used in included studies.

Ref	Animal	*In vivo*	*Ex vivo*	Macroscopic				Microscopic										Electrophysiology		Assessment timing		
				Gross	Slit lamp	Fundoscopy	ΔT	H/E	LM	TUNEL	MB/AII	TB	TdB	SHGI	TEM	SEM	Investigative assays	ffERG	fVEP	≤1hr	>1Hr	
69	Rabbit	-	**✓**	-	-	-	**✓**	**✓**	-	-	-	-	-	-	-	-	-	-	-	**✓**	-	
62	Rabbit	-	**✓**	-	-	-	**✓**	-	**✓**	-	-	-	-	-	-	-	-	-	-	**✓**	**✓**	**(90m)**
60	Rabbit	-	**✓**	-	-	-	**✓**	-	-	-	**✓**	-	-	-	**✓**	**✓**	-	-	-	**✓**	**-**	
36	Rabbit	**✓**	-	-	-	-	-	**✓**	**✓**	-	-	-	-	-	**✓**	-	-	-	-	**✓**	**✓**	**(2d)**
37	Rat	**✓**	-	-	-	-	-	**✓**	-	-	-	-	-	-	-	-	-	-	-	-	**✓**	**(2d)**
64	Cow	-	**✓**	-	-	-	-	-	-	-	-	-	-	-	-	-	-	-	-	-	-	
82	Rat	**✓**	-	-	-	-	-	**✓**	-	-	-	-	-	-	-	-	-	-	-	-	**✓**	**(7d)**
75	Rat	**✓**	-	-	-	-	-	-	-	-	-	-	-	-	-	-	-	-	-	-	-	
38	Rat	**✓**	-	-	-	-	-	**✓**	-	-	-	-	-	-	-	-	-	-	-	-	**✓**	**(4d)**
77	Mouse	**✓**	-	-	-	-	-	-	-	-	-	-	-	-	-	-	-	-	-	-	-	
76	Rat	**✓**	-	-	-	-	-	-	-	-	-	-	-	-	-	-	-	-	-	-	-	
33	Rat	**✓**	-	**✓**	-	-	-	**✓**	-	-	-	-	-	-	-	-	-	-	-	-	**✓**	**(3, 28d)**
61	Rabbit	-	**✓**	-	-	-	**✓**	**✓**	-	-	-	-	-	-	-	-	-	-	-	**✓**	-	
28	Rat	**✓**	-	**✓**	-	-	**✓**	-	-	**✓**	-	-	**✓**	-	-	-	-	-	-	**✓**	**✓**	**(1, 7, 8, 30d)**
154	Rat	**✓**	-	-	-	-	-	**✓**	-	-	-	**✓**	-	-	-	-	-	-	-	-	**✓**	**(12Hr)**
66	Rat	**✓**	-	-	-	-	-	**✓**	-	-	-	-	-	-	-	-	-	-	-	-	**✓**	**(1d)**
65	Rabbit	**✓**	-	-	**✓**	-	-	**✓**	-	-	-	-	-	-	-	-	-	-	-	-	**✓**	**(1, 3d)**
34	Rat	**✓**	-	-	-	-	-	-	-	-	-	-	-	-	-	-	-	-	-	-	-	
71	Rabbit	-	**✓**	-	-	-	**✓**	-	-	-	-	-	-	-	-	-	-	-	-	**✓**	-	
70	Rabbit	-	**✓**	-	-	-	**✓**	**✓**	-	-	-	-	-	-	-	-	-	-	-	**✓**	-	
39	Rabbit	**✓**	-	-	-	**✓**	**✓**	**✓**	-	-	-	-	-	-	-	-	-	**✓**	-	**✓**	**✓**	**(1, 7, 14d)**
44	Mouse	**✓**	-	-	-	-	-	-	-	-	-	-	-	-	-	-	-	-	-	-	-	
16	Rabbit	**✓**	-	-	-	-	**✓**	**✓**	**-**	-	-	-	-	-	-	-	-	-	-	**✓**	**✓**	**(90m)**
74	Rabbit	-	**✓**	-	**✓**	**✓**	**✓**	**✓**	-	-	-	-	-	-	-	-	-	-	-	**✓**	-	
49	Pig	-	**✓**	-	-	-	-	-	-	-	-	-	-	-	-	**✓**	-	-	-	-	-	
83	Rat	**✓**	-	-	-	-	-	**✓**	-	-	-	-	-	-	-	-	-	-	-	-	**✓**	**(5d)**
35	Rat	**✓**	-	-	-	-	-	**✓**	-	-	-	-	-	-	-	-	-	-	-	-	**✓**	**(4d)**
73	Rabbit	-	**✓**	-	-	-	-	-	-	-	-	-	-	**✓**	-	-	-	-	-	**✓**	-	
46	Rabbit	**✓**	-	-	-	-	-	**✓**	-	-	-	-	-	-	**✓**	-	-	-	**✓**	**✓**	**✓**	**(28d)**
17	Rat	**✓**	-	-	-	-	-	**✓**	-	-	-	-	-	-	-	-	-	-	-	-	**✓**	**(1d)**
72	Rabbit	-	**✓**	-	-	-	**✓**	-	-	-	-	-	-	-	-	-	-	-	-	**✓**	-	
40	Pig	-	**✓**	-	-	-	**✓**	**✓**	-	-	-	-	-	-	-	-	-	-	-	**✓**	-	
41	Rabbit	-	**✓**	-	-	-	**✓**	-	-	-	-	-	-	-	-	-	-	-	-	**✓**	-	
45	Mouse	**✓**	**✓**	-	-	-	-	-	-	-	-	-	-	-	-	-	-	-	-	**✓**	-	
42	Pig & cow	-	**✓**	-	-	-	-	**✓**	-	-	-	-	-	-	-	-	-	-	-	**✓**	-	
15	Rat & mouse	**✓**	-	-	-	-	-	**✓**	-	-	-	-	-	-	-	-	**✓**	-	-	**✓**	-	
43	pig or Rabbit	**✓**	**✓**	-	-	-	**✓**	**✓**	-	-	-	-	-	-	-	-	-	-	-	-	-	

**ΔT**: Change in temperature, **H/E** Hematoxylin and Eosin, **LM** Light Microscopy, **TUNEL** Terminal deoxynucleotidyl transferase dUTP nick end labelling, **MB/AII** Methylene Blue and Azure II, **TB** Trypan Blue, **TdB** Toluidine Blue, **SHGI** Second Harmonic Generation Imaging, **TEM** Transmission Electron Microscopy**, SEM** Scanning Electron Microscopy**, Investigative assays** include Ter119, CD41, fibrinogen and Glial Fibrillary Acidic Protein,** ffERG** full field Electroretinogram**, fVEP** flash Visual Evoked Potentials.

**Table 9:** Summary of ultrasound delivery assessments and quantification methods according to the route of delivery.

			Route of ultrasound delivery				Method to assess successful delivery									Method to quantify delivered molecule								
Ref.	*In vivo*	*Ex vivo*	Trans-topical	Direct injection into target tissue	BRB	V/VR	Plasmid expression	Fluorescent dyes	Fluorescently labelled dextrans	Fluorescently labelled nanoparticles	MRI	Drug delivery	Therapeutic outcome in disease model	Intracellular protein dynamics	Electrophysiology	Spectrophotometry	Quantitative microscopy	Flow cytometry	RT-PCR	Western blot	ELISA	MRI intensity	f-VEP	Qualitative scoring
69	-	✓	✓	-	-	-	-	-	-	-	-	✓	-	-	-	✓	-	-	-	-	-	-	-	-
62	-	✓	✓	-	-	-	-	✓	-	-	-	-	-	-	-	✓	-	-	-	-	-	-	-	-
60	-	✓	✓	-	-	-	-	✓	-	-	-	-	-	-	-	✓	-	-	-	-	-	-	-	-
36	✓	-	-	✓	-	-	✓	-	-	-	-	-	-	-	-	-	-	-	-	-	-	-	-	✓
37	✓	-	-	✓	-	-	✓	-	-	-	-	-	-	-	-	-	-	-	-	-	-	-	-	✓
64	-	✓	-	-	-	✓	-	-	-	✓	-	-	-	-	-	-	✓	✓	-	-	-	-	-	-
82	✓	-	-	✓	-	-	✓	-	-	-	-	-	-	-	-	-	✓	-	-	-	-	-	-	-
75	✓	-	-	✓	✓	✓	✓	-	-	-	-	-	✓	-	-	-	-	-	✓	-	-	-	-	-
38	✓	-	-	✓	-	-	✓	-	-	-	-	-	-	-	-	-	✓	-	-	-	-	-	-	-
77	✓	-	-	-	✓	✓	✓	-	-	-	-	-	-	-	-	-	-	-	✓	-	-	-	-	-
76	✓	-	-	-	✓	✓	-	-	-	-	-	-	✓	-	-	-	✓	-	-	-	-	-	-	-
33	✓	-	-	-	-	✓	✓	-	-	-	-	-	-	-	-	-	✓	-	-	-	-	-	-	-
61	-	✓	✓	-	-	-	-	✓	-	-	-	-	-	-	-	-	✓	-	-	-	-	-	-	-
28	✓	-	-	✓	-	-	✓	-	-	-	-	-	-	-	-	✓	✓	-	-	-	-	-	-	-
154	✓	-	-	-	-	✓	-	-	-	✓	-	-	-	-	-	-	✓	-	-	-	-	-	-	-
66	✓	-	-	-	✓	-	-	-	-	-	✓	-	-	-	-	-	-	-	-	-	-	✓	-	-
65	✓	-	-	-	-	✓	✓	-	-	-	-	-	-	-	-	-	✓	-	-	-	-	-	-	-
34	✓	-	-	-	-	✓	✓	-	-	-	-	-	✓	-	-	-	-	-	✓	-	✓	-	-	✓
71	-	✓	✓	-	-	-	-	✓	-	-	-	-	-	-	-	-	-	✓	-	-	-	-	-	-
70	-	✓	✓	-	-	-	-	✓	-	-	-	✓	-	-	-	✓	-	-	-	-	-	-	-	-
39	✓	-	✓	-	-	-	-	-	✓	-	-	-	-	-	-	✓	-	-	-	-	-	-	-	-
44	✓	-	-	✓	-	-	✓	-	-	-	-	-	✓	-	-	-	-	-	✓	✓	-	-	-	-
16	✓	-	✓	-	-	-	-	-	-	-	-	✓	-	-	-	✓	-	-	-	-	-	-	-	-
74	-	✓	✓	-	-	-	-	-	-	✓	-	-	-	-	-	✓	✓	-	-	-	-	-	-	-
49	-	✓	✓	-	-	-	-	✓	-	-	-	-	-	-	-	-	✓	-	-	-	-	-	-	-
83	✓	-	-	✓	-	-	✓	-	-	-	-	-	-	-	-	-	✓	-	-	-	-	-	-	-
35	✓	-	-	✓	-	-	✓	-	-	-	-	-	-	-	-	-	-	-	✓	-	-	-	-	✓
73	-	✓	✓	-	-	-	-	-	-	✓	-	-	-	-	-	-	✓	-	-	-	-	-	-	-
46	✓	-	-	-	-	✓	-	-	-	-	-	-	-	-	✓	-	-	-	-	-	-	-	✓	✓
17	✓	-	-	-	-	✓	✓	-	-	✓	-	-	-	-	-	-	-	✓	✓	-	✓	-	-	-
72	-	✓	✓	-	-	-	-	-	✓	-	-	-	-	✓	-	-	✓	-	-	-	-	-	-	-
40	-	✓	-	-	-	✓	-	-	-	✓	-	-	-	-	-	✓	-	-	-	-	-	-	-	-
41	-	✓	✓	-	-	-	-	✓	-	-	-	-	-	-	-	✓	-	-	-	-	-	-	-	-
45	✓	✓	✓	-	-	-	-	✓	-	-	-	-	-	-	-	-	✓	-	-	-	-	-	-	-
42	-	✓	-	-	-	✓	-	-	-	✓	-	-	-	-	-	✓	-	-	-	-	-	-	-	-
15	✓	-	-	-	✓	✓	✓	-	-	-	✓	-	-	-	-	-	✓	-	-	-	-	✓	-	-
43	✓	✓	✓	-	-	-	-	✓	-	-	-	-	-	-	-	✓	✓	-	-	-	-	-	-	-

**Table 10:** summary of the effect of ultrasound on the delivery of various tracer molecules with differing physicochemical properties.

Ref	Changing factor	Route of delivery	- ultrasound	+ ultrasound
			Effect	
70	Charge & Lipophilicity	Transcorneal	After 60 minutes coincubation, the negatively charged (-2), mildly lipophobic sodium fluorescein and the negatively charged (-2), and comparatively lipophilic dexamethasone sodium phosphate achieved moderate (5 x 10^-7^ cm/s) and poor (0.11 x 10^-7^ cm/s) transcorneal permeability, respectively, whilst positively charged (+5), severely lipophobic tobramycin achieved a higher 25 x 10^-7^ cm/s permeability.	After 5 minutes of 400 kHz ultrasound, and 60 minutes coincubation, tobramycin permeability increased by 5 x 10^-7^ cm/s, dexamethasone permeability increased by 127% to 0.25 x 10^-7^ cm/s, and fluorescein permeability increased by 149% to 12 x 10^-7^ cm/s.
69	Lipophilicity	Transscleral	Corneal permeability of all beta blockers tested was limited to 0.4-1.2 x 10^-5^ cm/s after 60 minutes coincubation.	Ultrasound increased the permeability of all tested beta blockers after 60 minutes of ultrasound, however, highly lipophilic beta blockers showed improved permeability compared to less lipophilic beta blockers.
72	weight	Transscleral	After 15 minutes coincubation, the penetration depth of 20kDa FITC-dextrans was greater than larger 70 kDa and 150 kDa dextrans at 100, 60 and 50 µm, respectively.	Transscleral penetration of FITC-dextrans appeared inversely proportional to size, with 20 kDa, 70 kDa and 150 kDa dextrans achieving penetration distances of 360, * 300 and 178 µm, respectively.
64	diameter	V/VR	PEGylated polystyrene nanospheres with a hydrodynamic diameter of 52, 131 and 218 nm achieved poor vitreoretinal uptake into RPE cells after 2-4 hours coincubation.	52 nm nanospheres achieved an RPE cell uptake 17-fold higher compared to control after 30 s ultrasound. 131 nm nanospheres required 120 s of ultrasound to achieve a 9.4-fold increase in cell uptake, whilst larger 218 nm nanospheres failed to improve their delivery even after 120 s sonication and 2-4 hours of coincubation.

*The 20kDa dextrans were delivered through the entire scleral thickness.

**Table 11:**
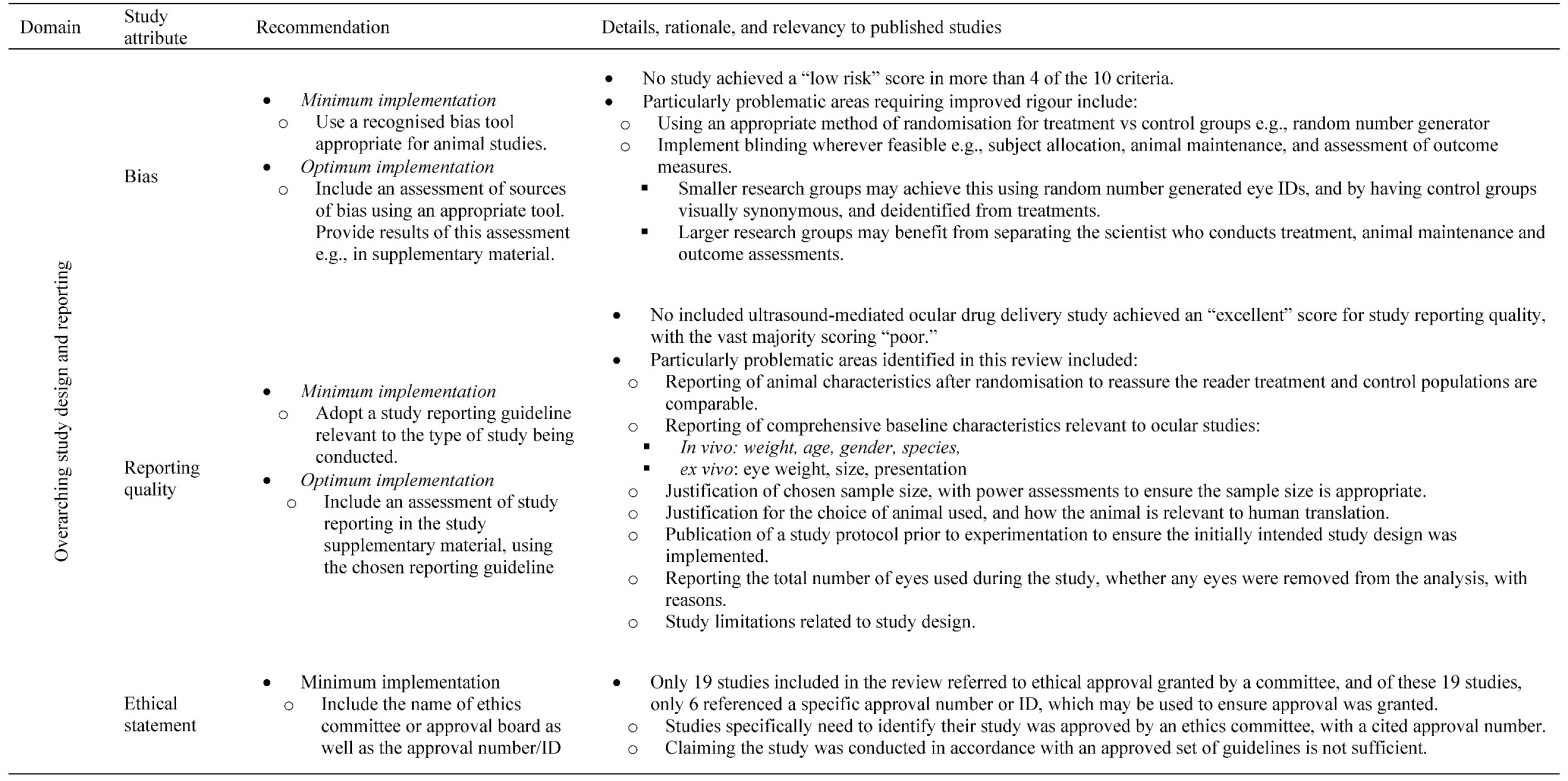

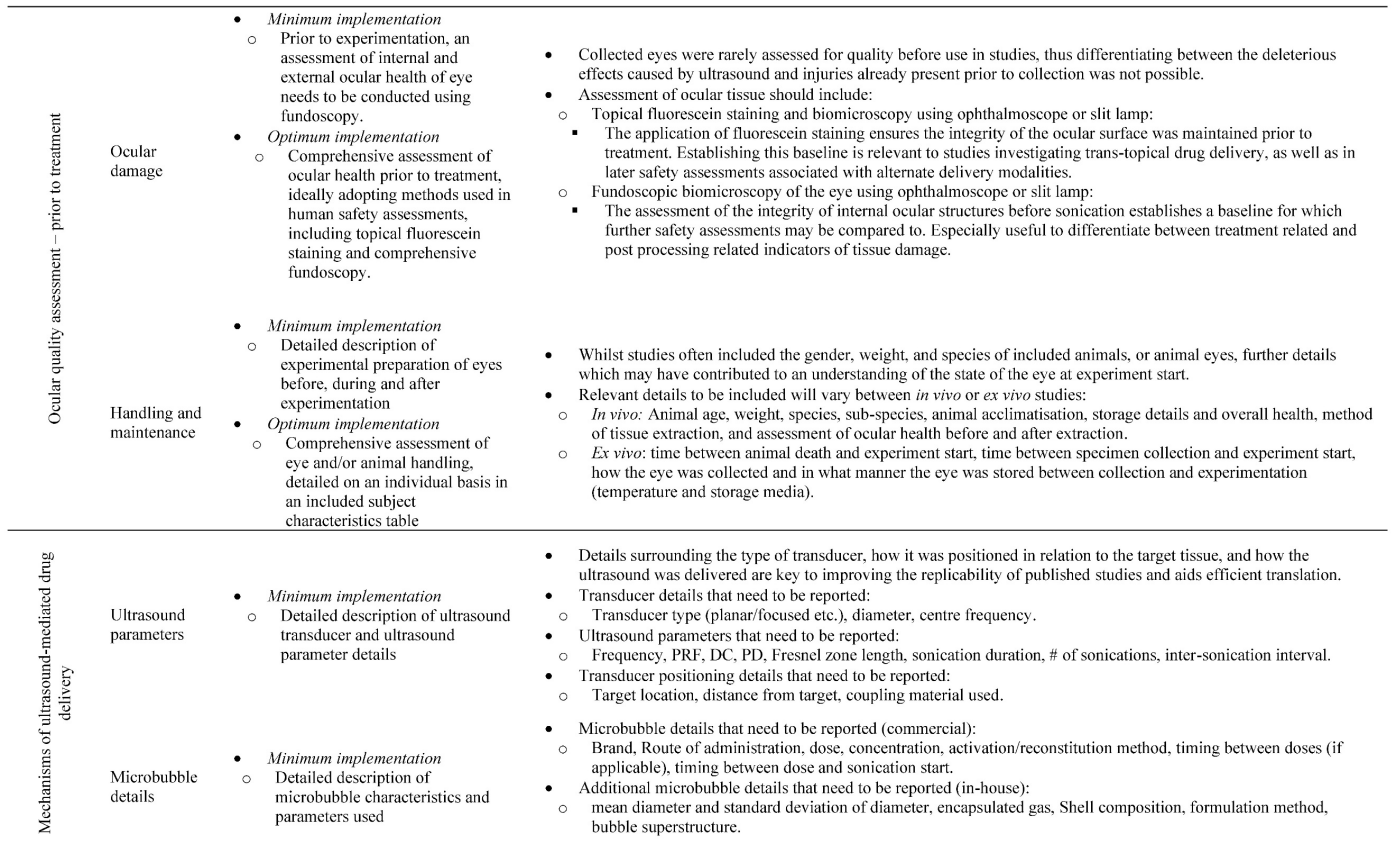

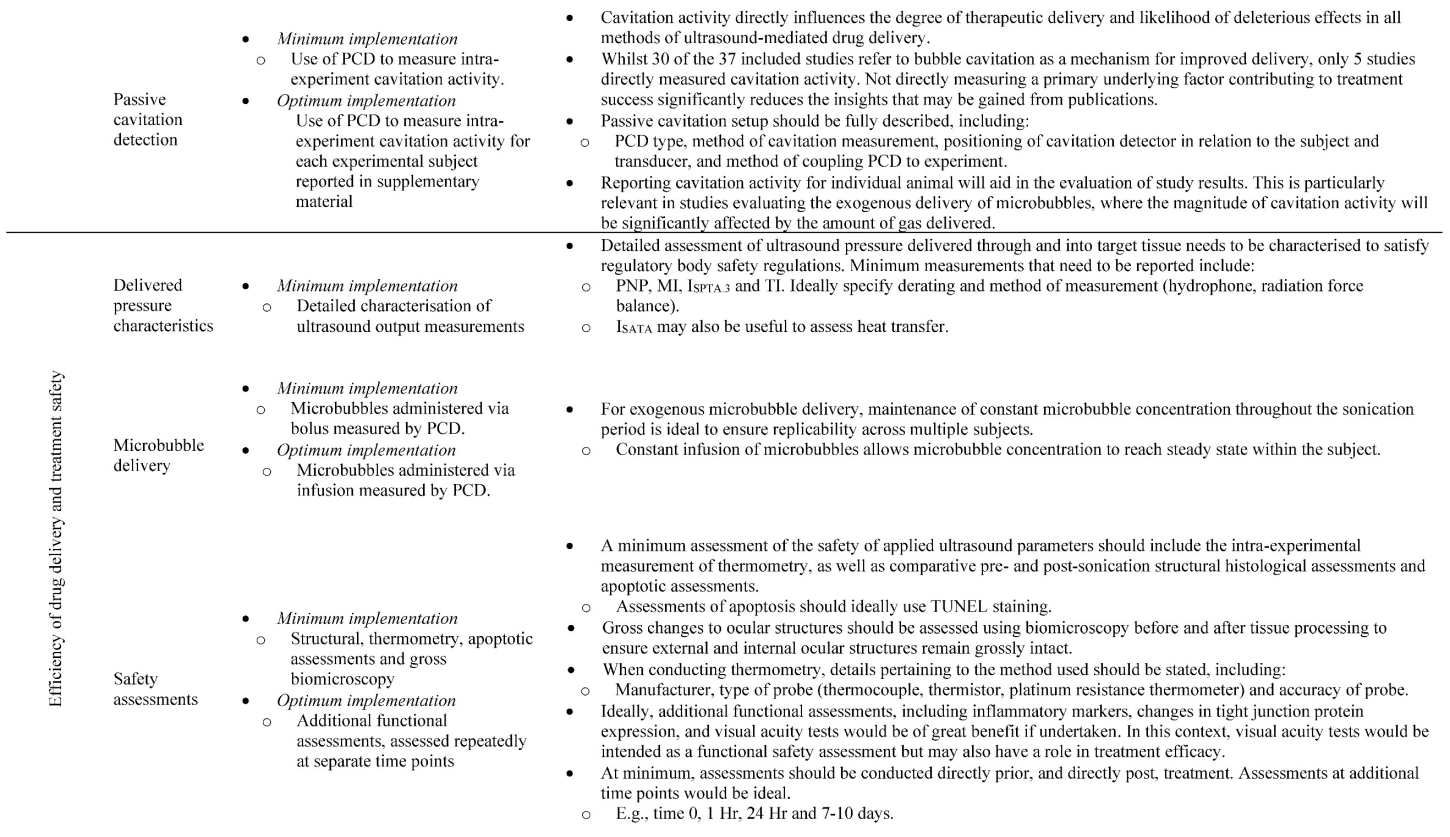

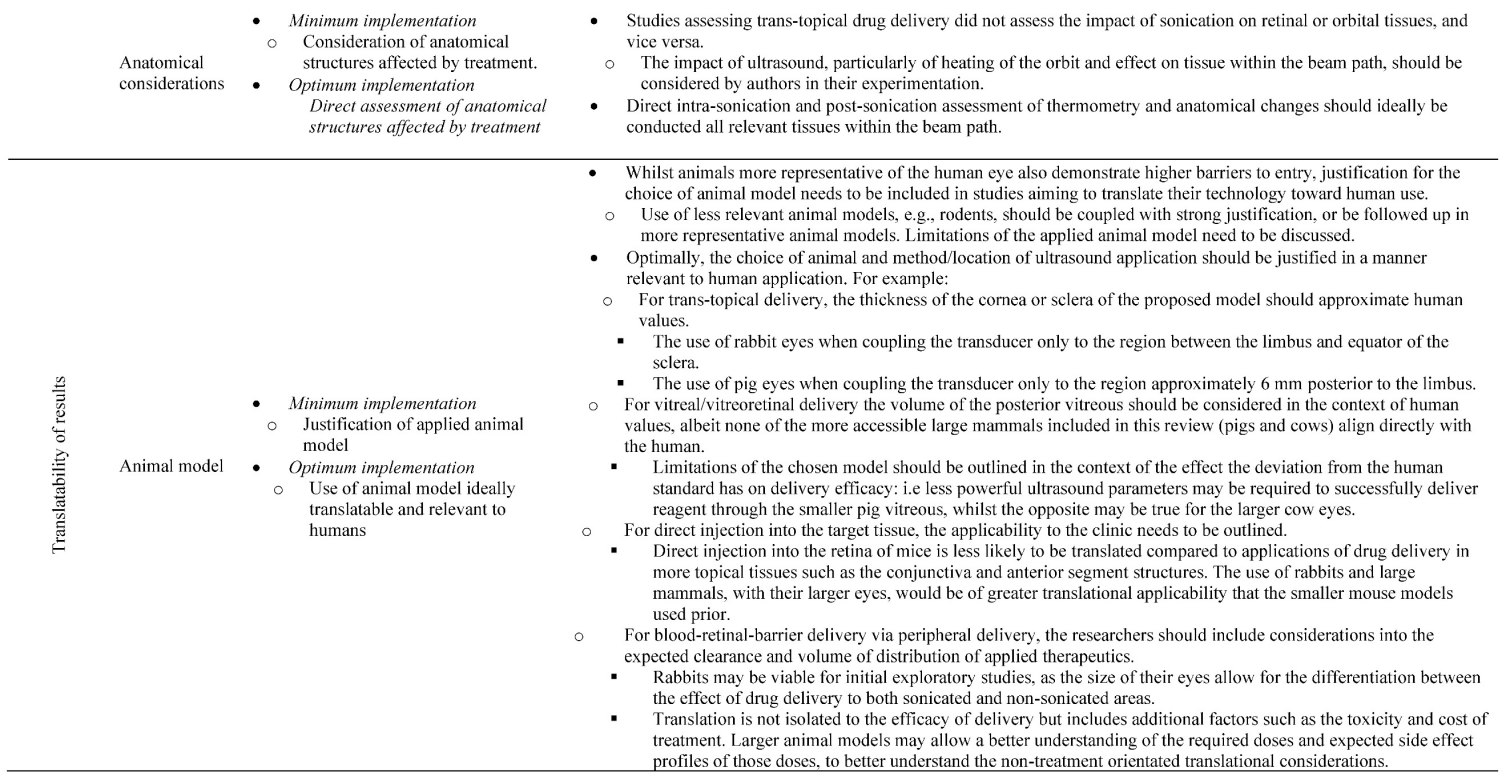
Guide to improved preclinical study design and reporting in ultrasound-mediated ocular drug delivery.

## Abbreviations

RoB: Risk of Bias
ARRIVE 2.0: Animal Research Reporting of *In Vivo* Experiments
SYRCLE: Systematic Review Centre for Laboratory animal Experimentation
AMD: age-related macular degeneration
DRE: diabetic retinopathy
BRB: blood-retinal barrier
RPE: retinal pigment epithelium
PCC: population, concept, context
MBP_EXO_: Exogenous microbubble barrier permeation
AS: acoustic streaming
MBP_ENDO_: endogenous microbubble barrier permeation
ILM: inner limiting membrane
BRB_i_: inner blood-retinal barrier
BRB_o_: outer blood-retinal barrier
ZO-1, -2, -3: zonula occludens-1, -2, -3
JAM: junctional adhesion molecule
p-gp: p-glycoprotein
MRP: multidrug resistance-associated proteins
BAB: blood-aqueous barrier
LAT1: L-type amino acid transporter 1
US-FDA: United States Food and Drug Administration
ELIP: echogenic liposome
PEDF: pigment epithelium-derived factor
CNV: choroidal neovascularisation
GF-β2: growth factor-β2
PDGF-B: platelet-derived growth factor-B
mNGF: mouse nerve growth factor
PNP: peak negative pressure
PCD: passive cavitation detector
PPP: peak positive pressure
PD: pulse duration
PRP: pulse repetition period
PRF: pulse repetition frequency
SD: sonication duration
DC: duty cycle
I_SA_: spatial average intensity
I_SP_: spatial peak intensity
I_TP_: temporal peak intensity
I_TA_: temporal average intensity
I_PA_: pulse average intensity
I_SPTP_: the maximum intensity found in a pulse that is measured in the focal zone
I_SPTA_: the measured intensity of one pulse repetition period measured in the focal zone
I_SPPA_: the measured intensity averaged over the entire pulse duration measured in the focal zone
I_SATP_: the maximum intensity averaged over the entire area
I_SATA_: the average intensity of one pulse repetition period averaged over the entire area
I_SAPA_: the average intensity over one pulse duration of the entire area
I_SPTA.3_: the spatial peak temporal average intensity derated by 0.3dB MHz^-1^ cm^-1^
RFB: radiation force balance
MI: mechanical index
SEM: scanning electron microscopy
TEM: transmission electron microscopy
MCD: microbubble volume dose
I_vit_: Intravitreal
RB: retrobulbar
IV: intravenous
DPPG: 1,2-Dipalmitoyl-*sn*-glycero-3-phosphoglycerol
DSPC: 1,2-Distearoyl-*sn*-glycero-3-phosphocholine
DPPA: 1,2-Dipalmitoyl-*sn*-glycero-3-phosphate
DPPC: 1,2-Dipalmitoyl-*sn*-glycero-3-phosphocholine
MPEG_5000_ DPPE: N-(Carbonyl-methoxypolyethylenglycol 5000)-1,2-dipalmitoyl-sn-glycero-3-phosphoethanolamine
DSPE-PEG(2k)-OMe: 1,2-distearoyl-sn-glycero-3-phosphatidyl-ethanolamine-(methoxy-polyethleneglycol 2000)
DSPE: 1,2-distearoyl-sn-glycero-3-phosphoethanolamine
DSPA: 1,2-distearoyl-sn-glycero-phosphoacid
DPPE: 1,2-*Dipalmitoyl*-sn-Glycero-3-*Phosphatidylethanolamine*
ΔT: Change in temperature
H/E: Hematoxylin and Eosin
LM: Light Microscopy
TUNEL: Terminal deoxynucleotidyl transferase dUTP nick end labelling
MB/AII: Methylene Blue and Azure II
TB: Trypan Blue
TdB: Toluidine Blue
SHGI: Second Harmonic Generation Imaging
ffERG: full field Electroretinogram
fVEP: flash Visual Evoked Potentials
ALARA: as low as reasonable achievable
ROS: reactive oxygen species
BMUS: the British Medical Ultrasound Society
TI: thermal index
FFA: fluorescein fundus angiography
TGF- β2: tissue growth factor-β2
PVR: proliferative vitreoretinography
p53: tumour protein p53
Rb94: retinoblastoma tumour suppressor gene
Gd-DTPA: gadopentetate dimeglumine

## References

[1] United Nations Department of Economic and Social Affairs Population Division (2022). World population prospects 2022. 27 ed. New York: United Nations.

[2] Yang X, Chen H, Zhang T, Yin X, Man J, He Q (2021). Global, regional, and national burden of blindness and vision loss due to common eye diseases along with its attributable risk factors from 1990 to 2019: a systematic analysis from the global burden of disease study 2019. Aging (Albany N Y).

[3] Furtado JM, Jonas J, Peto T, Steinmetz JD, Briant PS, Wong TY (2021). Global vision loss due to age-related macular degeneration. Invest Ophthalmol Vis Sci.

[4] Peto T, Resnikoff S, Kempen JH, Steinmetz JD, Briant PS, Wong TY (2021). Diabetic retinopathy contributes to global vision loss. Invest Ophthalmol Vis Sci.

[5] Campbell M, Humphries P (2012). The blood-retina barrier: tight junctions and barrier modulation. Adv Exp Med Biol.

[6] Gote V, Sikder S, Sicotte J, Pal D (2019). Ocular drug delivery: present innovations and future challenges. J Pharmacol Exp Ther.

[7] Del Amo EM, Rimpela AK, Heikkinen E, Kari OK, Ramsay E, Lajunen T (2017). Pharmacokinetic aspects of retinal drug delivery. Prog Retin Eye Res.

[8] van der Reis MI, La Heij EC, De Jong-Hesse Y, Ringens PJ, Hendrikse F, Schouten JS (2011). A systematic review of the adverse events of intravitreal anti-vascular endothelial growth factor injections. Retina.

[9] Aptel F, Lafon C (2012). Therapeutic applications of ultrasound in ophthalmology. Int J Hyperthermia.

[10] Miller DL, Smith NB, Bailey MR, Czarnota GJ, Hynynen K, Makin IR (2012). Overview of therapeutic ultrasound applications and safety considerations. J Ultrasound Med.

[11] Gandhi K, Barzegar-Fallah A, Banstola A, Rizwan SB, Reynolds JNJ (2022). Ultrasound-mediated blood-brain barrier disruption for drug delivery: a systematic review of protocols, efficacy, and safety outcomes from preclinical and clinical studies. Pharmaceutics.

[12] Lafond M, Aptel F, Mestas J-L, Lafon C (2017). Ultrasound-mediated ocular delivery of therapeutic agents: a review. Expert Opin Drug Deliv.

[13] Du J, Du LF, Li FH, Zheng XZ, Li HL, Shi QS (2011). Ultrasound targeted microbubble destruction-mediated gene delivery system: Application to therapy for ocular disease. Asian Biomed.

[14] Rousou C, Schuurmans CCL, Urtti A, Mastrobattista E, Storm G, Moonen C (2021). Ultrasound and microbubbles for the treatment of ocular diseases: From preclinical research towards clinical application. Pharmaceutics.

[15] Touahri Y, Dixit R, Kofoed RH, Miloska K, Park E, Raeisossadati R (2020). Focused ultrasound as a novel strategy for noninvasive gene delivery to retinal Muller glia. Theranostics.

[16] Nabili M, Shenoy A, Chawla S, Mahesh S, Liu J, Geist C (2014). Ultrasound-enhanced ocular delivery of dexamethasone sodium phosphate: An *in vivo* study. J Ther Ultrasound.

[17] Du J, Sun Y, Li FH, Du LF, Duan YR (2017). Enhanced delivery of biodegradable mPEG-PLGA-PLL nanoparticles loading Cy3-labelled PDGF-BB siRNA by UTMD to rat retina. J Biosci.

[18] Yang CD, Jessen J, Lin KY (2022). Ultrasound-assisted ocular drug delivery: a review of current evidence. J Clin Ultrasound.

[19] Page MJ, McKenzie JE, Bossuyt PM, Boutron I, Hoffmann TC, Mulrow CD (2021). The PRISMA 2020 statement: An updated guideline for reporting systematic reviews. PLoS Med.

[20] Hooijmans CR, Rovers MM, de Vries RBM, Leenaars M, Ritskes-Hoitinga M, Langendam MW (2014). SYRCLE's risk of bias tool for animal studies. BMC Med Res Methodol.

[21] Percie du Sert N, Hurst V, Ahluwalia A, Alam S, Avey MT, Baker M (2020). The ARRIVE guidelines 2.0: updated guidelines for reporting animal research. BMJ Open Sci.

[22] García-González M, Muñoz F, González-Cantalapiedra A, López-Peña M, Saulacic N (2021). Systematic review and quality evaluation using ARRIVE 2.0 guidelines on animal models used for periosteal distraction osteogenesis. Animals (Basel).

[23] Wang S, Mahesh SP, Liu J, Geist C, Zderic V (2012). Focused ultrasound facilitated thermo-chemotherapy for targeted retinoblastoma treatment: a modeling study. Exp Eye Res.

[24] Nabili M, Geist C, Zderic V (2015). Thermal safety of ultrasound-enhanced ocular drug delivery: A modeling study. Med Phys.

[25] Su H, Liu S, Wang Z, Xie W, Jiang B, Xiong H (2009). *In vivo* transfection of enhanced green fluorescent protein in rat retinal ganglion cells mediated by ultrasound-induced microbubbles. Neural Regen Res.

[26] Zderic V, Clark JI, Vaezy S (2005). Ocular drug delivery using ultrasound. 4th International Symposium on Therapeutic Ultrasound. Kyoto.

[27] Chau Y, Suen WL, Jiang J, Qu J, Zeng Y (2013). Mechanism of ultrasound-mediated transscleral delivery: Temporary alteration of scleral structure increases permeability of macromolecules. Invest Ophthalmol Vis Sci.

[28] Kowalczuk L, Boudinet M, El Sanharawi M, Touchard E, Naud M-C, Saïed A (2011). *In vivo* gene transfer into the ocular ciliary muscle mediated by ultrasound and microbubbles. Ultrasound Med Biol.

[29] Zhang H, Yang D, Wigg JP, Unger H, Unger M, Crowston JG (2014). Ultrasound-mediated transscleral delivery of Avastin to the posterior segment of rabbit eye *in vivo*. Invest Ophthalmol Vis Sci.

[30] Bae JH, Park HS, Shim SH, Kim JM (2014). Effect of low-intensity ultrasound on the transscleral delivery of serum protein. Invest Ophthalmol Vis Sci.

[31] Nabili M, Mahesh S, Geist C, Zderic V (2015). Ultrasound-enhanced delivery of anti-inflammatory ophthalmic drugs. Ultrasound Med Biol.

[32] Wang M, Yang Q, Li M, Zou H, Wang Z, Ran H (2020). Multifunctional nanoparticles for multimodal imaging-guided low-intensity focused ultrasound/immunosynergistic retinoblastoma therapy. ACS Appl Mater Interfaces.

[33] Xie W, Liu S, Su H, Wang Z, Zheng Y, Fu Y (2010). Ultrasound microbubbles enhance recombinant adeno-associated virus vector delivery to retinal ganglion cells *in vivo*. Acad Radiol.

[34] Zheng X, Du L, Wang H, Gu Q (2012). A novel approach to attenuate proliferative vitreoretinopathy using ultrasound-targeted microbubble destruction and recombinant adeno-associated virus-mediated RNA interference targeting transforming growth factor-beta2 and platelet-derived growth factor-B. J Gene Med.

[35] Li H, Qian J, Yao C, Wan C, Li F (2016). Combined ultrasound-targeted microbubble destruction and polyethylenimine-mediated plasmid DNA delivery to the rat retina: Enhanced efficiency and accelerated expression. J Gene Med.

[36] Sonoda S, Tachibana K, Uchino E, Okubo A, Yamamoto M, Sakoda K (2006). Gene transfer to corneal epithelium and keratocytes mediated by ultrasound with microbubbles. Invest Ophthalmol Vis Sci.

[37] Yamashita T, Sonoda S, Suzuki R, Arimura N, Tachibana K, Maruyama K (2007). A novel bubble liposome and ultrasound-mediated gene transfer to ocular surface: RC-1 cells *in vitro* and conjunctiva *in vivo*. Exp Eye Res.

[38] Li HL, Zheng XZ, Wang HP, Li F, Wu Y, Du LF (2009). Ultrasound-targeted microbubble destruction enhances AAV-mediated gene transfection in human RPE cells *in vitro* and rat retina *in vivo*. Gene Ther.

[39] Suen W-LL, Wong HS, Yu Y, Lau LCM, Lo AC-Y, Chau Y (2013). Ultrasound-mediated transscleral delivery of macromolecules to the posterior segment of rabbit eye *in vivo*. Invest Ophthalmol Vis Sci.

[40] Huang D, Chen Y-S, Thakur SS, Rupenthal ID (2017). Ultrasound-mediated nanoparticle delivery across *ex vivo* bovine retina after intravitreal injection. Eur J Pharm Biopharm.

[41] Lamy R, Chan E, Lee O-T, Phone A, Salgaonkar VA, Diederich CJ (2018). 880 kHz ultrasound treatment for drug delivery to the vitreous humor. Am J Transl Res.

[42] Thakur SS, Chen Y-S, Houston ZH, Fletcher N, Barnett NL, Thurecht KJ (2019). Ultrasound-responsive nanobubbles for enhanced intravitreal drug migration: An *ex vivo* evaluation. Eur J Pharm Biopharm.

[44] Gao R, Zhou X, Yang Y, Wang Z (2014). Transfection of wtp53 and Rb94 Genes Into Retinoblastomas of Nude Mice by Ultrasound-Targeted Microbubble Destruction. Ultrasound Med Biol.

[45] Jegal U, Lee JH, Lee J, Jeong H, Kim MJ, Kim KH (2019). Ultrasound-assisted gatifloxacin delivery in mouse cornea, *in vivo*. Sci Rep.

[46] Shen X, Huang LA-O, Ma D, Zhao J, Xie Y, Li Q Ultrasound microbubbles enhance the neuroprotective effect of mouse nerve growth factor on intraocular hypertension-induced neuroretina damage in rabbits. J Ophthalmol. 2016: 4235923.

[47] Balasubramaniam B, Chong YJ, Azzopardi M, Logeswaran A, Denniston AK (2022). Topical anti-Inflammatory agents for non-infectious uveitis: current treatment and perspectives. J Inflamm Res.

[48] Jumelle C, Gholizadeh S, Annabi N, Dana R (2020). Advances and limitations of drug delivery systems formulated as eye drops. J Control Release.

[49] Murugappan SK, Zhou Y (2015). Transsclera drug delivery by pulsed high-intensity focused ultrasound (HIFU): an *ex vivo* study. Curr Eye Res.

[50] Ambati J, Adamis AP (2002). Transscleral drug delivery to the retina and choroid. Prog Retin Eye Res.

[51] Sridhar MS (2018). Anatomy of cornea and ocular surface. Indian J Ophthalmol.

[52] Toris CB, Gagrani M, Ghate D (2021). Current methods and new approaches to assess aqueous humor dynamics. Expert Rev Ophthalmol.

[53] Smith DW, Lee CJ, Gardiner BS No flow through the vitreous humor: How strong is the evidence? Prog Retin Eye Res. 2020: 100845.

[54] Alm A, Bill A (1973). Ocular and optic nerve blood flow at normal and increased intraocular pressures in monkeys (Macaca irus): a study with radioactively labelled microspheres including flow determinations in brain and some other tissues. Exp Eye Res.

[55] Kim SH, Lutz RJ, Wang NS, Robinson MR (2007). Transport barriers in transscleral drug delivery for retinal diseases. Ophthalmic Res.

[56] Shafaie S, Hutter V, Brown MB, Cook MT, Chau DYS (2018). Diffusion through the *ex vivo* vitreal body - bovine, porcine, and ovine models are poor surrogates for the human vitreous. Int J Pharm.

[57] Silva AF, Pimenta F, Alves MA, Oliveira MSN (2020). Flow dynamics of vitreous humour during saccadic eye movements. J Mech Behav Biomed Mater.

[58] Wilson CG, Tan LE, Mains J (2011). Principles of retinal drug delivery from within the vitreous. In: Kompella UB, Edelhauser HF, editors. Drug Product Development for the Back of the Eye. Boston, MA: Springer US.

[59] Hughes PM, Olejnik O, Chang-Lin JE, Wilson CG (2005). Topical and systemic drug delivery to the posterior segments. Adv Drug Deliv Rev.

[60] Zderic V, Clark JI, Martin RW, Vaezy S (2004). Ultrasound-enhanced transcorneal drug delivery. Cornea.

[61] Cheung AC, Yu Y, Tay D, Wong HS, Ellis-Behnke R, Chau Y (2010). Ultrasound-enhanced intrascleral delivery of protein. Int J Pharm.

[62] Zderic V, Clark JI, Vaezy S (2004). Drug delivery into the eye with the use of ultrasound. J Ultrasound Med.

[63] United States Food and Drug Administration (2019). Marketing clearance of diagnostic ultrasound systems and transducers: guidance for industry and Food and Drug Administration staff. In: U.S Department of Health and Human Services, editor.

[64] Peeters L, Lentacker I, Vandenbroucke RE, Lucas B, Demeester J, Sanders NN (2008). Can ultrasound solve the transport barrier of the neural retina?. Pharm Res.

[65] Sonoda S, Tachibana K, Yamashita T, Shirasawa M, Terasaki H, Uchino E (2012). Selective gene transfer to the retina using intravitreal ultrasound irradiation. J Ophthalmol.

[66] Park J, Zhang Y, Vykhodtseva N, Akula JD, McDannold NJ (2012). Targeted and reversible blood-retinal barrier disruption via focused ultrasound and microbubbles. PLoS One.

[67] Pandit R, Koh WK, Sullivan RKP, Palliyaguru T, Parton RG, Gotz J (2020). Role for caveolin-mediated transcytosis in facilitating transport of large cargoes into the brain via ultrasound. J Control Release.

[68] Sanwal R, Joshi K, Ditmans M, Tsai SSH, Lee WL (2021). Ultrasound and microbubbles for targeted drug delivery to the lung endothelium in ARDS: cellular mechanisms and therapeutic opportunities. Biomedicines.

[69] Zderic V, Vaezy S, Martin RW, Clark JI (2002). Ocular drug delivery using 20-kHz ultrasound. Ultrasound Med Biol.

[70] Nabili M, Patel H, Mahesh SP, Liu J, Geist C, Zderic V (2013). Ultrasound-enhanced delivery of antibiotics and anti-inflammatory drugs into the eye. Ultrasound Med Biol.

[71] Lamy R, Chan E, Zhang H, Salgaonkar VA, Good SD, Porco TC (2013). Ultrasound-enhanced penetration of topical riboflavin into the corneal stroma. Invest Ophthalmol Vis Sci.

[72] Chau Y, Suen WL, Tse HY, Wong HS (2017). Ultrasound-enhanced penetration through sclera depends on frequency of sonication and size of macromolecules. Eur J Pharm Sci.

[73] Suen W-LL, Jiang J, Wong HS, Qu J, Chau Y (2016). Examination of effects of low-frequency ultrasound on scleral permeability and collagen network. Ultrasound Med Biol.

[74] Huang D, Wang L, Dong Y, Pan X, Li G, Wu C (2014). A novel technology using transscleral ultrasound to deliver protein loaded nanoparticles. Eur J Pharm Biopharm.

[75] Zhou X-y, Liao Q, Pu Y-m, Tang Y-q, Gong X, Li J (2009). Ultrasound-mediated microbubble delivery of pigment epithelium-derived factor gene into retina inhibits choroidal neovascularization. Chin Med J (Engl).

[76] Li T, Han Y, Zhang H, Xu LJ, Xiang Y (2010). Targeting therapy of pigment epithelium derived factor mediated by ultrasound activated immunoliposome for choroidal neovascularization. Chin Ophthalmic Res.

[77] Luo J, Zhou X, Diao L, Wang Z (2010). Experimental research on wild-type p53 plasmid transfected into retinoblastoma cells and tissues using an ultrasound microbubble intensifier. J Int Med Res.

[78] Chen H, Hwang JH (2013). Ultrasound-targeted microbubble destruction for chemotherapeutic drug delivery to solid tumors. J Ther Ultrasound.

[79] Delaney LJ, Isguven S, Eisenbrey JR, Hickok NJ, Forsberg F (2022). Making waves: how ultrasound-targeted drug delivery is changing pharmaceutical approaches. Mater Adv.

[80] Peruzzi G, Sinibaldi G, Silvani G, Ruocco G, Casciola CM (2018). Perspectives on cavitation enhanced endothelial layer permeability. Colloids Surf B Biointerfaces.

[81] Sharma D, Leong KX, Czarnota GJ (2022). Application of ultrasound combined with microbubbles for cancer therapy. Int J Mol Sci.

[82] Zheng X, Li H, Du L, Wang H, Gu Q (2009). *In vivo* and *in vitro* effects of ultrasound or/and microbubbles on recombinant adeno-associated virus-mediated transgene expression in the retina. Asian Biomed.

[83] Wan C, Qian J, Li F, Li H (2015). Ultrasound-targeted microbubble destruction enhances polyethylenimine-mediated gene transfection *in vitro* in human retinal pigment epithelial cells and *in vivo* in rat retina. Mol Med Rep.

[84] Polat BE, Hart D, Langer R, Blankschtein D (2011). Ultrasound-mediated transdermal drug delivery: mechanisms, scope, and emerging trends. J Control Release.

[85] Rooze J, Rebrov EV, Schouten JC, Keurentjes JT (2013). Dissolved gas and ultrasonic cavitation-a review. Ultrason Sonochem.

[86] Slama RBH, Gilles B, Chiekh MB, Bera JC (2019). Characterization of focused-ultrasound-induced acoustic streaming. Exp Therm Fluid Sci.

[87] Eckart C (1948). Vortices and streams caused by sound waves. Phys Rev.

[88] Wiklund M (2022). Ultrasonic enrichment of microparticles in bioaffinity assays [Doctoral thesis]. Stockholm: Royal Institute of Technology.

[89] Sharpe J, Greated C, Gray C, Campbell D (1989). The measurement of acoustic streaming using particle image velocimetry. Acta Acust United Acust.

[90] Oyama T, Imashiro C, Kuriyama T, Usui H, Ando K, Azuma T (2021). Acoustic streaming induced by MHz-frequency ultrasound extends the volume limit of cell suspension culture. J Acoust Soc Am.

[91] Shutilov VA (1980). Fundamental physics of ultrasound. London: Gordon and Breach Science Publishers.

[92] Alsadiq H, Tupally K, Parekh H, Veidt M (2018). Influence of acoustofluidic parameters on velocity streaming of sonicated medical microbubbles. Acoustics. Adelaide.

[93] Supponen O, Upadhyay A, Lum J, Guidi F, Murray T, Vos HJ (2020). The effect of size range on ultrasound-induced translations in microbubble populations. J Acoust Soc Am.

[94] White FM, Majdalani J (2006). Viscous fluid flow. 3rd ed. New York: McGraw-Hill.

[95] De Luca R, Forzoni L, Gelli F, Bamber J (2021). An educational overview of ultrasound probe types and their fields of application. Arch Acoust.

[96] Hendee WR, Ritenour ER (2002). Medical imaging physics. 4th ed. New York: Wiley-Liss.

[97] David C, Tiwari R (2015). Ultrasound in maxillofacial imaging: a review. J med radiol pathol surg.

[98] Noll D (2006). Ultrasound notes, part II - Diffraction. Michigan: University of Michigan.

[99] Silverman RH (2016). Focused ultrasound in ophthalmology. Clin Ophthalmol.

[100] Izadifar Z, Izadifar Z, Chapman D, Babyn P (2020). An introduction to high intensity focused ultrasound: systematic review on principles, devices, and clinical applications. J Clin Med.

[101] Civale J, Rivens I, Shaw A, Ter Haar G (2018). Focused ultrasound transducer spatial peak intensity estimation: a comparison of methods. Phys Med Biol.

[102] Benitez Martinez M, Baeza Moyano D, Gonzalez-Lezcano RA (2021). Phacoemulsification: Proposals for Improvement in Its Application. Healthcare (Basel).

[103] Zacharias J (2008). Role of cavitation in the phacoemulsification process. J Cataract Refract Surg.

[104] Abramowicz JS, Adhikari S, Dickman E, Estroff JA, Harris GR, Nomura J (2022). Ocular ultrasound: review of bioeffects and safety, including fetal and point of care perspective: review of bioeffects and safety, including fetal and point-of-care perspective. J Ultrasound Med.

[105] Coleman DJ, Silverman RH, Rondeau MJ, Lloyd HO, Daly S (2006). Explaining the current role of high frequency ultrasound In ophthalmic diagnosis (ophthalmic ultrasound). Expert Rev Ophthalmol.

[106] Gong Y, Cabodi M, Porter T (2010). Pressure-dependent resonance frequency for lipid-coated microbubbles at low acoustic pressures. IEEE Int Ultrason Symp.

[107] Doinikov AA, Haac JF, Dayton PA (2009). Resonance frequencies of lipid-shelled microbubbles in the regime of nonlinear oscillations. Ultrasonics.

[108] Roovers S, Segers T, Lajoinie G, Deprez J, Versluis M, De Smedt SC (2019). The role of ultrasound-driven microbubble dynamics in drug delivery: from microbubble fundamentals to clinical translation. Langmuir.

[109] Barwick C (1994). Developing a working knowledge of ultrasound intensities. J Diagn Med Sonogr.

[111] ter Haar G (2007). Therapeutic applications of ultrasound. Prog Biophys Mol Biol.

[112] Kikuchi T, Uchida T (2011). Calorimetric method for measuring high ultrasonic power using water as a heating material. J Phys Conf Ser.

[113] Nyborg WL, Carson PL, Dunn F, Miller DL, Miller MW, Ziskin MC (1985). Biological effects of ultrasound: mechanisms and clinical implications. J Acoust Soc Am.

[114] Sen T, Tufekcioglu O, Koza Y (2015). Mechanical index. Anatol J Cardiol.

[115] Szabo TL (2014). Chapter 13 - Ultrasonic Exposimetry and Acoustic Measurements. In: Szabo TL, editor. Diagnostic Ultrasound Imaging: Inside Out (Second Edition). Boston: Academic Press.

[116] Ter Haar G (2011). Ultrasonic imaging: safety considerations. Interface Focus.

[117] Choi JJ, Carlisle RC, Coviello C, Seymour L, Coussios CC (2014). Non-invasive and real-time passive acoustic mapping of ultrasound-mediated drug delivery. Phys Med Biol.

[118] Kobus Z, Krzywicka M, Starek-Wojcicka A, Sagan A (2022). Effect of the duty cycle of the ultrasonic processor on the efficiency of extraction of phenolic compounds from Sorbus intermedia. Sci Rep.

[119] Al-Jawadi S, Thakur SS (2020). Ultrasound-responsive lipid microbubbles for drug delivery: a review of preparation techniques to optimise formulation size, stability and drug loading. Int J Pharm.

[120] Borden MA, Song KH (2018). Reverse engineering the ultrasound contrast agent. Adv Colloid Interface Sci.

[121] Chowdhury SM, Abou-Elkacem L, Lee T, Dahl J, Lutz AM (2020). Ultrasound and microbubble mediated therapeutic delivery: underlying mechanisms and future outlook. J Control Release.

[122] Martinez P, Bottenus N, Borden M (2022). Cavitation characterization of size-isolated microbubbles in a vessel phantom using focused ultrasound. Pharmaceutics.

[123] Jangjou A, Meisami AH, Jamali K, Niakan MH, Abbasi M, Shafiee M (2021). The promising shadow of microbubble over medical sciences: from fighting wide scope of prevalence disease to cancer eradication. J Biomed Sci.

[124] Sujarittam K, Choi JJ (2022). The relationship between bubble concentration and the acoustic emission energy of separate frequency bands. JASA Express Lett.

[125] Wu SK, Chu PC, Chai WY, Kang ST, Tsai CH, Fan CH (2017). Characterization of different microbubbles in assisting focused ultrasound-induced blood-brain barrier opening. Sci Rep.

[126] Hutter JC, Luu HM, Mehlhaff PM, Killam AL, Dittrich HC (1999). Physiologically based pharmacokinetic model for fluorocarbon elimination after the administration of an octafluoropropane-albumin microsphere sonographic contrast agent. J Ultrasound Med.

[127] Segers T, Jong N, Lohse D, Versluis M (2015). Microbubbles for medical applications. RSC Nanosci Nanotechnol.

[128] Kabalnov A, Klein D, Pelura T, Schutt E, Weers J (1998). Dissolution of multicomponent microbubbles in the bloodstream: 1. Theory. Ultrasound Med Biol.

[129] Sirsi S, Borden M (2009). Microbubble compositions, properties and biomedical applications. Bubble Sci Eng Technol.

[130] Tu J, Guan J, Qiu Y, Matula TJ (2009). Estimating the shell parameters of SonoVue microbubbles using light scattering. J Acoust Soc Am.

[131] Doinikov AA, Bouakaz A (2011). Review of shell models for contrast agent microbubbles. IEEE Trans Ultrason Ferroelectr Freq Control.

[132] Versluis M, Stride E, Lajoinie G, Dollet B, Segers T (2020). Ultrasound contrast agent modeling: a review. Ultrasound Med Biol.

[133] Azami RH, Aliabouzar M, Osborn J, Kumar KN, Forsberg F, Eisenbrey JR (2022). Material properties, dissolution and time evolution of PEGylated lipid-shelled microbubbles: effects of the polyethylene glycol hydrophilic chain configurations. Ultrasound Med Biol.

[134] Alsadiq H (2021). Shell properties and concentration stability of acoustofluidic delivery agents. Phys Eng Sci Med.

[135] Tsutsui JM, Xie F Fau - Porter RT, Porter RT (2004). The use of microbubbles to target drug delivery. Cardiovasc Ultrasound.

[136] Pennesi ME, Neuringer M, Courtney RJ (2012). Animal models of age related macular degeneration. Mol Aspects Med.

[137] Gwon A (2008). The primate in cataract/IOL surgery. In: Tsonis PA, editor. Animal Models in Eye Research. London: Academic Press.

[138] Krebs MP, Collin GB, Hicks WL, Yu M, Charette JR, Shi LY (2017). Mouse models of human ocular disease for translational research. PLoS One.

[139] Volland S, Esteve-Rudd J, Hoo J, Yee C, Williams DS (2015). A comparison of some organizational characteristics of the mouse central retina and the human macula. PLoS One.

[140] Henriksson JT, McDermott AM, Bergmanson JP (2009). Dimensions and morphology of the cornea in three strains of mice. Invest Ophthalmol Vis Sci.

[141] Azhdam AM, Goldberg RA, Ugradar S (2020). *In vivo* measurement of the human vitreous chamber volume using computed tomography imaging of 100 eyes. Transl Vis Sci Technol.

[142] Cone-Kimball E, Nguyen C, Oglesby EN, Pease ME, Steinhart MR, Quigley HA (2013). Scleral structural alterations associated with chronic experimental intraocular pressure elevation in mice. Mol Vis.

[143] Prince JH (1960). Anatomy and histology of the eye and orbit in domestic animals. Illinois: CC. Thomas.

[144] Cheruvu NP, Kompella UB (2006). Bovine and porcine transscleral solute transport: influence of lipophilicity and the Choroid-Bruch's layer. Invest Ophthalmol Vis Sci.

[145] Faber C, Scherfig E, Prause JU, Sørensen KE (2014). Corneal thickness in pigs measured by ultrasound pachymetry *in vivo*. Scand J Lab Anim Sci.

[146] Ahn SJ, Hong HK, Na YM, Park SJ, Ahn J, Oh J Use of rabbit eyes in pharmacokinetic studies of intraocular drugs. J Vis Exp. 2016: 113.

[147] Vurgese S, Panda-Jonas S, Jonas JB (2012). Scleral thickness in human eyes. PLoS One.

[148] Francis PJ, Appukuttan B, Simmons E, Landauer N, Stoddard J, Hamon S (2008). Rhesus monkeys and humans share common susceptibility genes for age-related macular disease. Hum Mol Genet.

[149] Umeda S, Ayyagari R, Allikmets R, Suzuki MT, Karoukis AJ, Ambasudhan R (2005). Early-onset macular degeneration with drusen in a cynomolgus monkey (macaca fascicularis) pedigree: exclusion of 13 candidate genes and loci. Invest Ophthalmol Vis Sci.

[150] Mustari MJ (2017). Nonhuman primate studies to advance vision science and prevent blindness. ILAR J.

[151] Balasubramanian M, Shabanian H (2020). Structural integrity and functional viability of *ex vivo* porcine eyes after longer-term saline suspension. Invest Ophthalmol Vis Sci.

[152] Ishtiaq R, Chaudhary MH, Rana MA, Jamil AR (2016). Psychosocial implications of blindness and low vision in students of a school for children with blindness. Pak J Med Sci.

[153] Ashby B, Garrett Q, Willcox MDP (2014). Corneal injuries and wound healing - review of processes and therapies. Austin J Clin Ophthalmol.

[154] Zheng X, Ji P, Hu J (2011). Sonoporation using microbubbles promotes lipofectamine-mediated siRNA transduction to rat retina. Bosn J Basic Med Sci.

[155] Kim C, Choi WJ, Ng Y, Kang W (2021). Mechanically induced cavitation in biological systems. Life (Basel).

[156] Tu J, Yu ACH (2022). Ultrasound-mediated drug delivery: sonoporation mechanisms, biophysics, and critical factors. BME Front.

[157] BMUS Safety Group (2009). Guidelines for the safe use of diagnostic ultrasound equipment part II: detailed guidelines. The British Medical Ultrasound Society.

[158] Belling JN, Heidenreich LK, Tian Z, Mendoza AM, Chiou TT, Gong Y (2020). Acoustofluidic sonoporation for gene delivery to human hematopoietic stem and progenitor cells. Proc Natl Acad Sci U S A.

[159] Helfield B, Chen X, Watkins SC, Villanueva FS (2016). Biophysical insight into mechanisms of sonoporation. Proc Natl Acad Sci U S A.

[160] Parchand S, Agrawal D, Ayyadurai N, Agarwal A, Gangwe A, Behera S (2022). Sympathetic ophthalmia: a comprehensive update. Indian J Ophthalmol.

[161] Bigelow TA, Church CC, Sandstrom K, Abbott JG, Ziskin MC, Edmonds PD (2011). The thermal index: its strengths, weaknesses, and proposed improvements. J Ultrasound Med.

[162] Hedrick WR, Hykes DL (1993). An overview of thermal and mechanical acoustic output indices. J Diagn Med Sonogr.

[163] Miller MW, Ziskin MC (1989). Biological consequences of hyperthermia. Ultrasound Med Biol.

[164] Ikoma T, Shibata T, Shibata N, Mito T, Kubo E, Sasaki H (2022). Acute cataract by a high-intensity focused ultrasound procedure: a case report. BMC Ophthalmol.

[166] Abbott JG (1999). Rationale and derivation of MI and TI - a review. Ultrasound Med Biol.

[167] Curley MG (1993). Soft tissue temperature rise caused by scanned, diagnostic ultrasound. IEEE Trans Ultrason Ferroelectr Freq Control.

[168] Praveen MR, Vasavada AR, Shah SK, Shah CB, Patel UP, Dixit NV (2009). Lens thickness of Indian eyes: impact of isolated lens opacity, age, axial length, and influence on anterior chamber depth. Eye (Lond).

[169] De Korte CL, van der Steen AFW, Thijssen JM (1994). Acoustic velocity and attenuation of eye tissues at 20 MHz. Ultrasound Med Biol.

[170] Martin R (2018). Cornea and anterior eye assessment with slit lamp biomicroscopy, specular microscopy, confocal microscopy, and ultrasound biomicroscopy. Indian J Ophthalmol.

[171] Gromov P, Moreira J, Gromova I, Celis J (2006). Proteomic analysis of urinary fibrinogen degradation products in patients with urothelial carcinomas. Clin Proteomics.

[172] Gain P, Thuret G, Chiquet C, Dumollard JM, Mosnier JF, Burillon C (2002). Value of two mortality assessment techniques for organ cultured corneal endothelium: trypan blue versus TUNEL technique. Br J Ophthalmol.

[173] Bringmann A, Iandiev I, Pannicke T, Wurm A, Hollborn M, Wiedemann P (2009). Cellular signaling and factors involved in Muller cell gliosis: neuroprotective and detrimental effects. Prog Retin Eye Res.

[174] Leinonen H, Tanila H (2018). Vision in laboratory rodents-Tools to measure it and implications for behavioral research. Behav Brain Res.

[176] Yang JJ, Huang CH, Yang CH, Yang CM, Lin CW, Ho TC (2021). The clinical contribution of full-field electroretinography and 8-year experiences of application in a tertiary medical center. J Pers Med.

[177] Qian X, Lu G, Thomas BB, Li R, Chen X, Shung KK (2022). Noninvasive ultrasound retinal stimulation for vision restoration at high spatiotemporal resolution. BME Front.

[178] Srikantha N, Mourad F, Suhling K, Elsaid N, Levitt J, Chung PH (2012). Influence of molecular shape, conformability, net surface charge, and tissue interaction on transscleral macromolecular diffusion. Exp Eye Res.

[179] Ambati J, Canakis CS, Miller JW, Gragoudas ES, Edwards A, Weissgold DJ (2000). Diffusion of high molecular weight compounds through sclera. Invest Ophthalmol Vis Sci.

[180] Kaczmarek JC, Kowalski PS, Anderson DG (2017). Advances in the delivery of RNA therapeutics: from concept to clinical reality. Genome Med.

[181] Shahryari A, Burtscher I, Nazari Z, Lickert H (2021). Engineering gene therapy: advances and barriers. Adv Ther.

[182] Paunovska K, Loughrey D, Dahlman JE (2022). Drug delivery systems for RNA therapeutics. Nat Rev Genet.

[183] Kothari R, Bokariya P, Singh S, Singh R (2016). A comprehensive review on methodologies employed for visual evoked potentials. Scientifica (Cairo).

[184] Idrees S, Sridhar J, Kuriyan AE (2019). Proliferative vitreoretinopathy: a review. Int Ophthalmol Clin.

[185] Wong ES, Choy RW, Zhang Y, Chu WK, Chen LJ, Pang CP (2022). Global retinoblastoma survival and globe preservation: a systematic review and meta-analysis of associations with socioeconomic and health-care factors. Lancet Glob Health.

[186] Conti A, Kamimura HAS, Novell A, Duggento A, Toschi N (2020). Magnetic resonance methods for focused ultrasound-induced blood-brain barrier opening. Front Phys.

[187] O'Reilly MA, Hynynen K (2012). Blood-brain barrier: real-time feedback-controlled focused ultrasound disruption by using an acoustic emissions-based controller. Radiology.

[188] Macleod MR, Lawson McLean A, Kyriakopoulou A, Serghiou S, de Wilde A, Sherratt N (2015). Risk of bias in reports of *in vivo* research: a focus for improvement. PLoS Biol.

[189] Harloff-Helleberg S, Nielsen LH, Nielsen HM (2017). Animal models for evaluation of oral delivery of biopharmaceuticals. J Control Release.

[190] Todo H (2017). Transdermal permeation of drugs in various animal species. Pharmaceutics.

[191] Netter FH, Machado CAG, Neumann PE, Tubbs RS, Brueckner JK, Gdowski MJ (2022). Netter atlas of human anatomy: classic regional approach. 8th ed. Philadelphia, PA: Elsevier.

[192] Thijssen JM, Mol HJ, Timmer MR (1985). Acoustic parameters of ocular tissues. Ultrasound Med Biol.

[193] Rohrbach D, Ito K, Lloyd HO, Silverman RH, Yoshida K, Yamaguchi T (2017). Material properties of human ocular tissue at 7-micron resolution. Ultrason Imaging.

[194] Rohrbach D, Jakob A, Lloyd HO, Tretbar SH, Silverman RH, Mamou J (2017). A novel quantitative 500-MHz acoustic microscopy system for ophthalmologic tissues. IEEE Trans Biomed Eng.

[195] Duck FA (1990). Physical properties of tissues: a comprehensive reference book. London: Academic Press Limited.

[196] Kotopoulis S, Popa M, Mayoral Safont M, Murvold E, Haugse R, Langer A (2022). SonoVue vs. Sonazoid vs. Optison: which bubble Is best for low-intensity sonoporation of pancreatic ductal adenocarcinoma?. Pharmaceutics.

[197] Tu J, Swalwell JE, Giraud D, Cui W, Chen W, Matula TJ (2011). Microbubble sizing and shell characterization using flow cytometry. IEEE Trans Ultrason Ferroelectr Freq Control.

[198] Hassan MA, Feril LB Jr, Suzuki K, Kudo N, Tachibana K, Kondo T (2009). Evaluation and comparison of three novel microbubbles: enhancement of ultrasound-induced cell death and free radicals production. Ultrason Sonochem.

[199] Chatterjee D, Sarkar K (2003). A Newtonian rheological model for the interface of microbubble contrast agents. Ultrasound Med Biol.

