# Supplementary material for: A systematic review of ultrasound-mediated drug delivery to the eye and critical insights to facilitate a timely path to the clinic
Source: Theranostics. 2023 Jun 19;13(11):3582–638. doi: 10.7150/thno.82884 (PMC10334839; doi:10.7150/thno.82884)
Supplement: Supplementary file 1 — Supplementary tables S1, S3, S4. [file thnov13p3582s1.pdf]

Table S1. Finalized search strategy for included databases.

**Database**	**Search strategy**	**Results**
PubMed	((((("Eye"[MeSH Terms])) OR (("Eye Diseases"[MeSH Terms]))) AND ((("ex vivo")) OR (("in vivo")))) AND (((("dosage form design")) OR (("drug delivery"))) OR (("Therapeutics"[MeSH Terms]) OR ("Drug-Related Side Effects and Adverse Reactions"[MeSH Terms])))) AND ((((("Ultrasonography"[MeSH Terms]) OR (ultrasound)) OR ("focused ultrasound")) OR ("MRI-guided ultrasound")) OR ("MR-guided focused ultrasound"))	249
Embase	(eye* OR 'eye disease' OR 'retinal disease')/br AND (('in vivo' OR 'ex vivo'):ti,ab,kw) AND ((therapy OR therapeutics OR 'drug dosage form' OR 'drug delivery method' OR 'adverse drug reactions' OR 'instillation')/br) AND ((ultrasonography OR ultrasound OR 'MRI-guided ultrasound' OR 'MR-guided focused ultrasound')/exp)	293
SCOPUS	(TITLE-ABS-KEY(("eye disease*") OR ("eye*") OR ("retina*") OR ("optic nerve") OR ("age-related macular degeneration") OR ("optic nerve disease*") OR ("amd") OR ("posterior eye*")) AND TITLE-ABS-KEY(("in vivo") OR ("ex vivo")) AND TITLE-ABS-KEY(("therap*") OR ("dosage form design") OR ("drug delivery") OR ("adverse drug reaction")) AND TITLE-ABS-KEY((ultrasound) OR (ultrasonography)))	161
CINAHL	TX (eye* OR "eye diseases" OR retina OR "optic nerve" OR "age-related macular degeneration" OR "optic nerve disease" OR amd OR "posterior eye disease") AND TX ("in vivo" or "ex vivo") AND AB ("therap*" OR "dosage form design" OR "drug delivery" OR "adverse drug reaction") AND TX (ultrasound or ultrasonography)	134

Example search strategy development process:

History and Search Details

Download

Search	Actions	Details	Query	Results
#20	•••	>	Search: ((((("Eye"[MeSH Terms])) OR (("Eye Diseases"[MeSH Terms]))) AND ((("ex vivo")) OR (("in vivo")))) AND (((("dosage form design")) OR (("drug delivery"))) OR (("Therapeutics"[MeSH Terms]) OR ("Drug-Related Side Effects and Adverse Reactions"[MeSH Terms])))) AND ((((("Ultrasonography"[MeSH Terms]) OR (ultrasound)) OR ("focused ultrasound")) OR ("MRI-guided ultrasound")) OR ("MR-guided focused ultrasound"))	249
#19	•••	>	Search: (((("Ultrasonography"[MeSH Terms]) OR (ultrasound)) OR ("focused ultrasound")) OR ("MRI-guided ultrasound")) OR ("MR-guided focused ultrasound")	1,857,123
#18	•••	>	Search: "MR-guided focused ultrasound"	294
#17	•••	>	Search: "MRI-guided ultrasound"	1
#16	•••	>	Search: "focused ultrasound"	7,595
#15	•••	>	Search: ultrasound	1,857,123
#14	•••	>	Search: "Ultrasonography"[MeSH Terms]	475,276
#13	•••	>	Search: ((("dosage form design")) OR (("drug delivery"))) OR (("Therapeutics"[MeSH Terms]) OR ("Drug-Related Side Effects and Adverse Reactions"[MeSH Terms]))	5,131,573
#12	•••	>	Search: ("Therapeutics"[MeSH Terms]) OR ("Drug-Related Side Effects and Adverse Reactions"[MeSH Terms])	5,089,154
#11	•••	>	Search: (("dosage form design")) OR (("drug delivery"))	140,908
#10	•••	>	Search: "Drug-Related Side Effects and Adverse Reactions"[MeSH Terms]	127,297
#9	•••	>	Search: "Therapeutics"[MeSH Terms]	4,995,359
#8	•••	>	Search: ("drug delivery")	140,764
#7	•••	>	Search: ("dosage form design")	199
#6	•••	>	Search: (("ex vivo")) OR (("in vivo"))	1,067,318
#5	•••	>	Search: ("ex vivo")	86,745
#4	•••	>	Search: ("in vivo")	1,010,400
#3	•••	>	Search: (("Eye"[MeSH Terms])) OR (("Eye Diseases"[MeSH Terms]))	794,046
#2	•••	>	Search: ("Eye"[MeSH Terms])	378,837
#1	•••	>	Search: ("Eye Diseases"[MeSH Terms])	615,128

Showing 1 to 20 of 20 entries

Table S3: Arrive 2.0 questionnaire for systematic review.

#	ARRIVE 2.0 item	Operational question	Answer		
			Yes	No	N/A
**Required set**					
1	1a. Study design	Are the treatment and control groups adequately described and comparable?			
2	1b. Study design	Are the individual experimental units clearly defined for accurate analysis, without the risk of pseudo-replication of results			
3	2a. Sample size	Are the exact number of experimental units allocated to each group (Tx or control) specified? Are the total number of animals used specified?			
4	2b. Sample size	Is it explained how the sample size was decided? Were power calculations used to determine sample size? If not, is there justification for the lack thereof?			
5	3a. Inclusion and exclusion criteria	Were any criteria for including or excluding animals during the experiment, or data points during the analysis specified? Were they established *a priori*, is there justification for the lack thereof?			
6	3b. Inclusion and exclusion criteria	For each experimental group, is it reported whether any animals, experimental units, or data points were not included in the analysis? Is it stated if there were no exclusions?			
7	3c. Inclusion and exclusion criteria	Is the exact n value included in analysis in each group reported?			
8	4a. Randomisation	Was randomisation used to allocate experimental units to control and treatment groups?			
9	4b. Randomisation	Was a strategy used to minimise the potential for confounders? E.g. order of treatments, and measurements, animal cage/location			
10	5. Blinding	Is it reported who was aware of the group allocation at the different stages of the experiment (during the allocation, the conduct of the experiment, the outcome assessment, and the data analysis)			
11	6a. Outcome measures	Are all the outcome measures explicitly described?			
12	6b. Outcome measures	In studies using inferential statistics (t test, ANOVA), was the primary outcome measure specified?			
13	7a. Statistical methods	Are details of the statistical methods used for each analysis included?			
14	7b. Statistical methods	Were there methods used to assess whether the data met the underlying assumptions of the statistical approach? (e.g. tests for normality to ensure the parametric tests' assumptions were valid)			
15	8a. Experimental animals	Were species-appropriate details of the animals given? (e.g. species, strain, sex, age and, if relevant, weight)			
16	8b. Experimental animals	Was further information on the provenance, health, and any previous procedures given to test animals?			
17	9a. Experimental procedures	Were enough details given for each experimental group, including controls, pertaining to the procedures, to allow others to replicate them? Including: what was done, how was it done and what was used?			
18	9b. Experimental procedures	Is it reported when and how often experimental procedures and measurements were given?			
19	9c. Experimental procedures	Is it stated if physiological acclimatisation after a stressful event, such as transport or treatments/handling were allowed before treatment or assessment? (if applicable)			
20	9d. Experimental procedures	Is it explained why experimental procedures were undertaken in the way they had been completed?			

21	10a. Results	Has a summary/descriptive statistic of the results for each experimental group been reported? Including a measure of variability where applicable?			
22	10b. Results	Has the effect size been reported with a confidence interval?			
**Recommended set**					
23	11. Abstract	Does the abstract provide an accurate summary of the research objectives, animal species, strain and sex, key methods, principal findings and study conclusions?			
24	12a. Background	Is there a sufficient scientific background to understand the rationale and context for the study? Is the experimental approach explained?			
25	12b. Background	Is it explained why the animal was used, or how the animal species and model used addresses the scientific objectives, and, where appropriate, the relevance to human biology?			
26	13. Objectives	Are the research question and objectives and, where appropriate, the specific hypothesis being tested clearly described?			
27	14. Ethical statement	Is the ethical review committee or equivalent which approved the use of animals in this study named? Has the use of animals been approved?			
28	15. Housing and husbandry	Are details of housing and husbandry conditions, including any environmental enrichment, provided?			
29	16a. Animal care and monitoring	Is it reported whether any interventions or steps were taken in the experimental protocols to reduce pain, suffering and distress?			
30	16b. Animal care and monitoring	Are expected or unexpected adverse events reported?			
31	16c. Animal care and monitoring	Are the humane endpoints established for the study? Is it reported what signs were monitored and the frequency of monitoring?			
32	17a. Interpretation/ scientific implications	Are the results interpreted whilst taking into account the study objectives, hypotheses, current theory and other relevant studies in the literature?			
33	17b. Interpretation/ scientific implications	Are the study limitations, including potential sources of bias, limitations of the animal model and imprecision with the results commented on?			
34	18. Generalisability/ translation	Is it commented on as to whether or how the findings of the study are likely to generalise to other species or experimental conditions, including any relevance to human biology?			
35	19. Protocol registration	Is it stated whether a protocol (including research questions, key design features and analysis plan) was prepared before the study, and if and where this protocol was registered?			
36	20. Data access	Is a statement provided describing if and where the study data are available?			
37	21a. Declaration of interests	Are any potential conflicts of interest, including financial and nonfinancial declared? If none exist, this should be stated			
38	21b. Declaration of interests	Are all funding sources (including grant identifier), and their role in the design, analysis and reporting of the study listed?			

Table S4. Determination of mean cow vitreal volume

<table>
<tr><td>Title</td><td>Measurement of cow eye vitreous volume</td></tr>
<tr><td>Date of experiment</td><td>08.12.2022 (DD.MM.YYYY)</td></tr>
<tr><td>Objective</td><td>Determine the average vitreal volume of cow eyes to include in the systematic review</td></tr>
<tr><td>Rationale</td><td>Currently literature is lacking in information related to the typical volume of the bovine eye vitreous</td></tr>
<tr><td>Experimental outline</td><td>1. Collect eyes in DMEM
2. Remove aqueous humour
3. Remove vitreous humour, centrifuge, and measure volume</td></tr>
<tr><td>Materials</td><td>1. Cow eyes
2. Pipettes and pipette tips
3. Tuberculin syringes
4. Centrifuge
5. 20ml Measuring cylinder
6. DMEM + P/S/L-Glut
7. Butterfly imaging ultrasound transducer</td></tr>
<tr><td>Method</td><td>1. Day 1
  1. Prepare DMEM in biosecurity cabinet
    a. Add 5 mL 100X Pen/Strep/L-Glut to each bottle of 500 mL DMEM
2. Day 2
  1. Collect cow eyes from Teys Australia Pty LTD
    a. Collect in DMEM, note which eyes relate to which cows
    b. Collect cold, in ice, note time of death and time of collection
  2. Remove majority of aqueous humour from anterior segment using tuberculin syringes
  3. Cut through cornea and remove remaining aqueous humour
  4. Remove lens
  5. Remove vitreous humour into 20ml falcon tube
  6. Measure weight of vitreous
  7. Determine vitreous volume using by applying density of 1.005g/cm^3</td></tr>
<tr><td>Animal details</td><td>
<table>
<tr><th>Mob</th><th>Body ID</th><th>Dentition</th><th>Gender</th><th>Body Weight (kg)</th><th>Bruise</th><th>Time of slaughter</th><th>Time of collection</th><th>Cow ID</th></tr>
<tr><td>407</td><td>312</td><td>0</td><td>Male</td><td>288.2</td><td>0</td><td>08:44</td><td>09:15</td><td>1</td></tr>
<tr><td>407</td><td>315</td><td>0</td><td>Male</td><td>292.2</td><td>0</td><td>08:46</td><td>09:16</td><td>3</td></tr>
<tr><td>407</td><td>316</td><td>0</td><td>Male</td><td>290.6</td><td>0</td><td>08:46</td><td>09:16</td><td>5</td></tr>
<tr><td>407</td><td>317</td><td>0</td><td>Male</td><td>271.6</td><td>0</td><td>08:47</td><td>09:17</td><td>7</td></tr>
<tr><td>407</td><td>318</td><td>0</td><td>Male</td><td>292.2</td><td>0</td><td>08:47</td><td>09:17</td><td>9</td></tr>
<tr><td>407</td><td>320</td><td>2</td><td>Male</td><td>292.8</td><td>0</td><td>08:48</td><td>09:18</td><td>11</td></tr>
<tr><td>407</td><td>321</td><td>0</td><td>Male</td><td>272.2</td><td>0</td><td>08:49</td><td>09:19</td><td>13</td></tr>
<tr><td>407</td><td>322</td><td>2</td><td>Male</td><td>295.2</td><td>0</td><td>08:50</td><td>09:19</td><td>15</td></tr>
<tr><td>407</td><td>323</td><td>0</td><td>Male</td><td>295</td><td>0</td><td>08:50</td><td>09:20</td><td>17</td></tr>
<tr><td>407</td><td>324</td><td>0</td><td>Male</td><td>275.2</td><td>0</td><td>08:51</td><td>09:20</td><td>19</td></tr>
</table>
Note, cow age is appraised according to dentition, where 0 = ≤ 18 months, and 2 = 18-30 months</td></tr>
<tr><td>Results</td><td>
<table>
<tr><th colspan="3">Weight measurements (g)</th></tr>
<tr><td>Eye ID</td><td>Left eye</td><td>Right eye</td></tr>
<tr><td>1</td><td>15.055</td><td>14.742</td></tr>
<tr><td>2</td><td>16.198</td><td>15.68</td></tr>
<tr><td>3</td><td>14.96</td><td>15.126</td></tr>
<tr><td>4</td><td>14.483</td><td>14.374</td></tr>
<tr><td>5</td><td>14.487</td><td>14.882</td></tr>
<tr><td>6</td><td>13.974</td><td>14.452</td></tr>
</table>
</td></tr>
</table>

7	15.257	15.31
8	13.997	13.581
9	15.2	14.6
10	13.504	13.72
Mean weight	14.712	14.647
**Bovine vitreous density = 1.005 ± 0.012 g/cm^3**		
**Cow vitreous volume (cm^3) calculated from density & weight**		
Cow	Left eye	Right eye
1	14.98	14.67
2	16.12	15.60
3	14.89	15.05
4	14.41	14.30
5	14.41	14.81
6	13.90	14.38
7	15.18	15.23
8	13.93	13.51
9	15.12	14.53
10	13.44	13.65
**Mean Volume**	**14.64**	**14.57**
**Mean Volume (all eyes)**	**14.61**	
**Standard deviation**	**±0.70**	

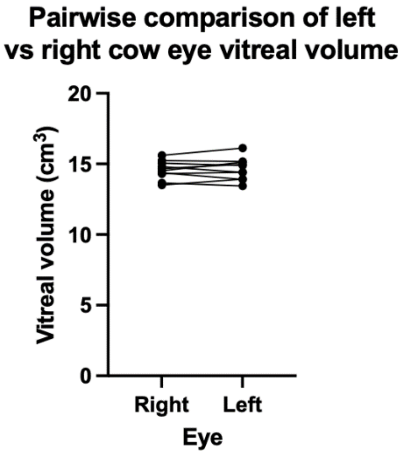

	Left	Right
Number of values	10	10
Minimum	13.44	13.51
Maximum	16.12	15.60
Range	2.681	2.089
Mean	14.64	14.57
Std. Deviation	0.7807	0.6559
Std. Error of Mean	0.2469	0.2074

Paired t test	
P value	0.6070
P value summary	ns
Significantly different (P < 0.05)?	No
One- or two-tailed P value?	Two-tailed
t, df	t=0.533, df=9
Number of pairs	10

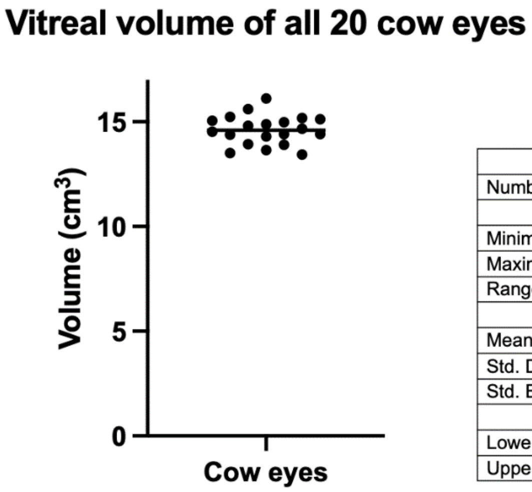

	Cow eyes
Number of values	20
Minimum	13.437
Maximum	16.117
Range	2.6806
Mean	14.606
Std. Deviation	0.70257
Std. Error of Mean	0.15710
Lower 95% CI of mean	14.277
Upper 95% CI of mean	14.935
